# On the application of 3d metals for C–H activation toward bioactive compounds: The key step for the synthesis of silver bullets

**DOI:** 10.3762/bjoc.17.126

**Published:** 2021-07-30

**Authors:** Renato L Carvalho, Amanda S de Miranda, Mateus P Nunes, Roberto S Gomes, Guilherme A M Jardim, Eufrânio N da Silva Júnior

**Affiliations:** 1 Institute of Exact Sciences, Department of Chemistry, Federal University of Minas Gerais - UFMG, CEP 31270-901, Belo Horizonte, MG, Brazil; 2 Department of Pharmaceutical Sciences, North Dakota State University, Fargo, ND, United States,; 3 Centre for Excellence for Research in Sustainable Chemistry (CERSusChem), Department of Chemistry, Federal University of São Carlos – UFSCar, CEP 13565-905, São Carlos, SP, Brazil

**Keywords:** bioactive compounds, C–H activation, 3d metals, drugs, medicinal chemistry

## Abstract

Several valuable biologically active molecules can be obtained through C–H activation processes. However, the use of expensive and not readily accessible catalysts complicates the process of pharmacological application of these compounds. A plausible way to overcome this issue is developing and using cheaper, more accessible, and equally effective catalysts. First-row transition (3d) metals have shown to be important catalysts in this matter. This review summarizes the use of 3d metal catalysts in C–H activation processes to obtain potentially (or proved) biologically active compounds.

## Introduction

The discovery of new biologically active substances represents not only an advance in the chemistry field but also offers innovative chances for pharmacological and biomedical sciences. Every year, several molecules are discovered and studied against different types of diseases, such as cancer [1–2], malaria [3–4], Chagas disease [5–6], HIV [7–8], depression [9–10], amnesia [11], Alzheimer [12], and maybe even in a more recent scenario, COVID-19 [13]. Even though many compounds are found to present activity against these diseases, only a few of them become approved, due to their toxicity or other issues related to their applicability. Therefore, synthetic methodologies that facilitate the successful production of potential biologically active molecules have a relevant role in the organic synthesis research field.

**Scheme 1:**
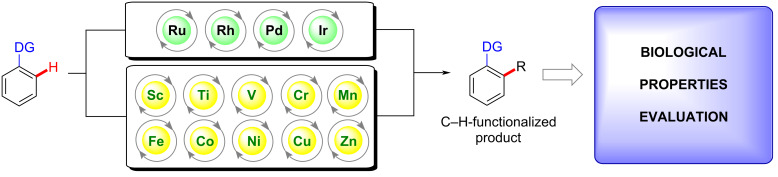
Schematic overview of transition metals studied in C–H activation processes.

One of the key synthetic methodologies is the C–H bond activation process that enables a straightforward access to several important and innovative compounds [14–18]. In the last few years, metals such as ruthenium [19–21], rhodium [22–24], palladium [25–27], and iridium [28–30] have been widely applied as catalysts for this matter, including in the synthesis of bioactive substances. Although catalysts based on these metals, are known to be efficient in C–H bond activation reactions affording the products in good yields and mild conditions, they are also known to be usually expensive. This fact may negatively affect the industrial application of synthetic procedures relying on such catalysts. The substitution by cheaper and more accessible metals such as any members of the first row of transition metals (Scheme 1) could overcome this drawback. The application of these metals as catalysts for C–H activation processes deserves a better exploration.

This review compilates the application of 3d metals as catalysts for C–H activation processes to obtain biologically active compounds or building blocks applied in the synthesis of molecules with known biological effects.

## Review

### Scandium-catalyzed C–H activation

**Scheme 2:**
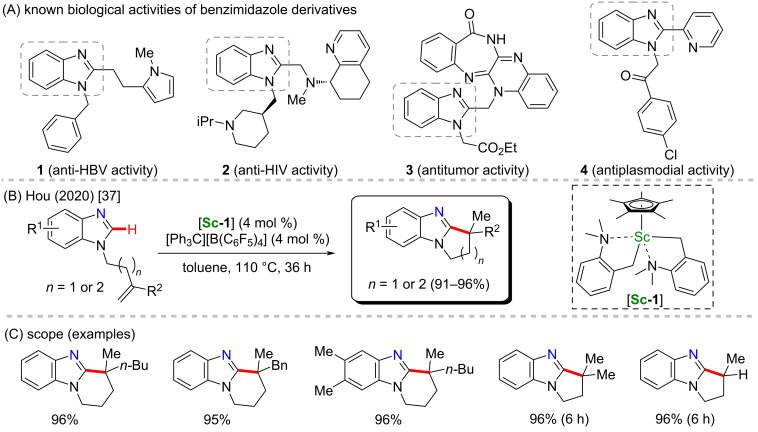
(A) Known biological activities related to benzimidazole-based compounds; (B and C) an example of a scandium-catalyzed C–H cyclization on benzimidazoles.

Scandium is the first metal of the 3d metals row. It is relatively cheap compared to heavier transition metals, and it is commonly used on catalytic procedures, such as catalyzed polymerization [31–32] and C–C coupling reactions [33–34]. It is also an increasing metal option to develop C–H activation methods, since it can be used as the metallic center of innovative and elaborated complexes [35–36]. Scandium-based catalysts have not been directly applied to the synthesis of known biologically active compounds via C–H activation reactions. Therefore, as challenging as it seems to be, there is still a demand for applying this relevant methodology to obtain new compounds with known pharmacological properties. However, several molecules based on structural scaffolds related to important biological activities have been successfully achieved via scandium-catalyzed C–H activation [37–41]. Hou’s group presented various studies on this theme [42–43], such as a notable work recently published in which they promoted a scandium-catalyzed intramolecular cyclization on benzimidazole substrates, via a C–H activation at the C-2 position (Scheme 2B). In this process, a scandium(III)/Cp* catalyst containing two units of an *o*-*N*,*N*-(dimethylamino)benzyl ligand [**Sc-1**] was applied, and several examples of cyclic benzimidazole compounds were obtained in excellent yields (Scheme 2C) [37]. Benzimidazole compounds bearing substituents in their C-2 position are present in several bioactive molecules. They are also known to present valuable biological activities, such as anti-HBV (**1**) [44], anti-HIV (**2**) [45], antitumor (**3**) [46] and even antiplasmodial activities (**4**) [47] (Scheme 2A). Although these studied bioactive compounds do not directly represent the structural moieties obtained in Hou’s group, this fact still gathers some critical value to the large variety of products obtained in the above-cited work. Incoming research works may lead to the observation of relevant activities and the applicability of this class of molecules.

**Scheme 3:**
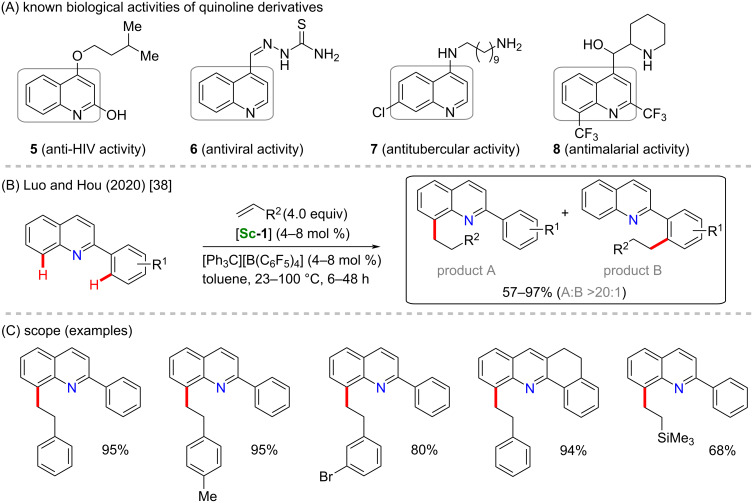
(A) Known biological activities related to quinoline-based compounds; (B and C) an example of a scandium-catalyzed C–H alkylation of 2-phenylquinoline derivatives.

Lou, Hou and co-workers also used the same catalyst to perform a regioselective scandium-catalyzed alkylation of 2-phenylquinoline derivatives (Scheme 3B) [38]. It is important to highlight the biological importance of quinoline derivatives, since several quinolines are known to present valuable biological activities, such as anti-HIV (**5**) [48], antiviral in general (**6**) [49], antituberculotic (**7**), and antimalarial (**8**) [50] (Scheme 3A). Using 2-phenylquinoline derivatives as substrates includes some intrinsic challenges, since there are two sites in the molecule where the C–H activation can take place. The authors were able to selectively obtain the C-8-substituted product (product A) in a proportion higher than 20:1 over the product resulting from C’-2 activation (product B) in good to excellent yields (Scheme 3C).

**Scheme 4:**
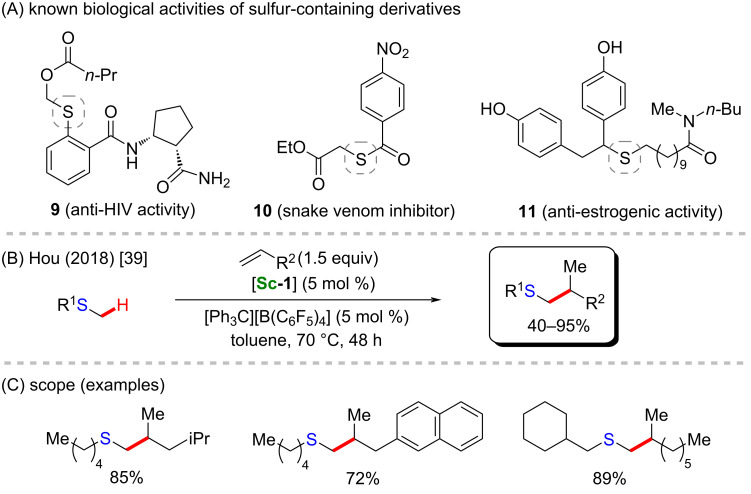
(A) Known biological activities related to sulfur-containing compounds; (B and C) an example of a scandium-catalyzed C(sp^3^)–H alkylation of methyl thioether derivatives.

Another biologically active structural motif that can be activated by this catalyst are thioethers, as was well described by Hou and co-workers in 2018 [39]. In this work, the presence of [**Sc-1**] and several alkenes resulted in the successful scandium-C(sp^3^)–H alkylation of methyl thioethers (Scheme 4B), by which different activated internal thioethers were obtained in good yields (Scheme 4C). The transformation facilitates direct access to several sulfur-containing pharmacological compounds that present valuable biological activities, such as anti-HIV (**9**) [51], inhibition of snake venom enzymes (**10**) [52], or even anti-estrogenic effects (**11**) [53] (Scheme 4A).

**Scheme 5:**
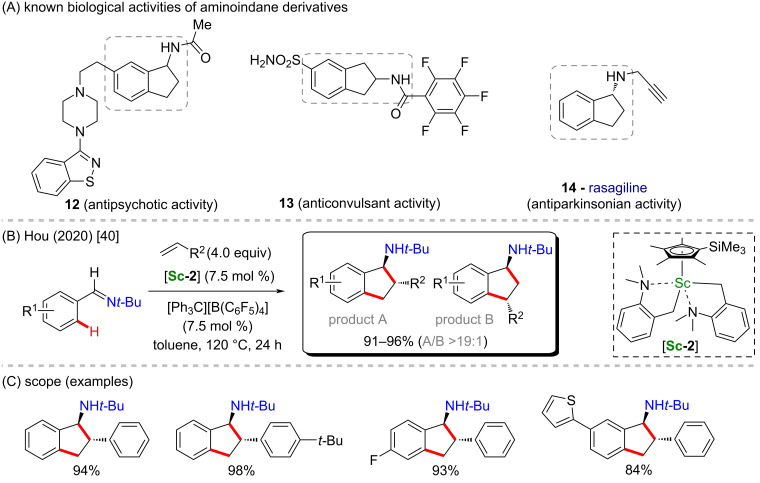
(A) Known biological activities related to aminoindane derivatives; (B and C) an example of a scandium-catalyzed C–H cyclization on aminoindane derivatives.

Recently, Hou and co-workers also explored the utility of a similar catalyst, [**Sc-2**], which bears a more electron-rich cyclopentadienyl ligand, in a scandium-catalyzed C–H [3 + 2] cyclization (Scheme 5B) [40]. In this transformation, several aminoindane derivatives were obtained from benzylimines in the presence of the catalyst [**Sc-2**], alkenes and [Ph_3_C][B(C_6_F_5_)_4_]. The desired aminoindane derivatives were obtained with good regio- and enantioselectivity, (product A/product B was observed in a ratio higher than 19:1, Scheme 5C). It is worth mentioning that aminoindanes are scaffolds also present in biologically active molecules that may present, for example, antipsychotic (**12**) [54], anticonvulsant (**13**) [55], and antiparkinsonian activities (**14**) [56–57] (Scheme 5A).

**Scheme 6:**
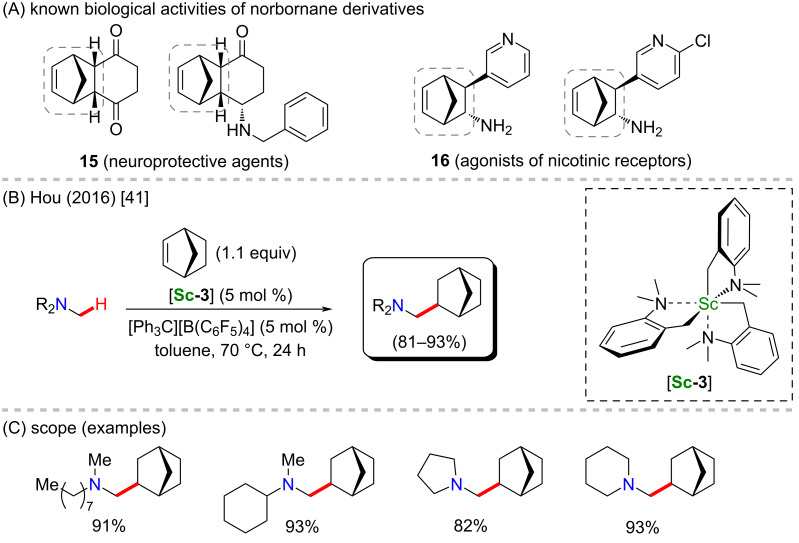
(A) Known biological activities related to norbornane derivatives; (B and C) an example of a scandium-catalyzed C–H hydroaminoalkylation of tertiary amines.

A few years earlier, Hou and co-workers reported the very first metal-catalyzed C–H hydroaminoalkylation of tertiary amines using norbornene as the coupling partner [41]. For this method, the scandium catalyst that presented the best performance was a homoleptic trialkylscandium, [**Sc-3**] (Scheme 6B), instead of the usual cyclopentadienyl-containing dialkylscandium catalysts described so far in this review. Several substituted norbornene derivatives were obtained in good to excellent yields (Scheme 6C). These bicyclic structures may present unique biological activities including neuroprotective properties (**15**), as it was recently reported by Joubert and co-workers [58], as well as acting as agonists of nicotinic receptors (**16**) [59], representing an alternative option for the treatment of cigarette addiction (Scheme 6A).

The use of scandium as the metallic motif in catalysts applied in C–H activation methodologies leads to the formation of important structures not yet been studied for their biological activities. Therefore, there is more to be achieved and studied, considering what scandium-based catalysts can still offer.

### Titanium-catalyzed C–H activation

**Scheme 7:**
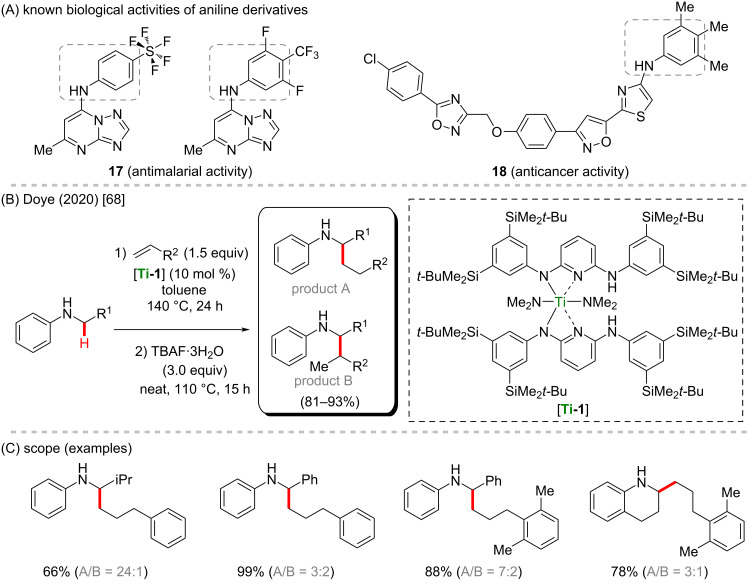
(A) Known biological activities related to aniline derivatives; (B and C) an example of a titanium-catalyzed C–H hydroaminoalkylation of *N*-alkylanilines.

Titanium is another well-known and considerably cheap 3d metal which is underexplored in the C–H activation field [60], especially with regard to the synthesis of biologically active compounds. Therefore, further studies on the applicability of this specific metal are highly desirable. Titanium is well known to be used as titanium dioxide, a powerful photocatalyst present in inks [61–62] and sunscreens [63–64]. As a catalyst, it can be used, for example, in polymerization methods to synthesize polypropylene [65]. With regard to C–H activation reactions, Doye’s group has dedicated its research to the development of significant titanium-catalyzed amine-directed C–H activation [66–67]. Recently, a good example was reported, in which C(sp^3^)–H hydroaminoalkylation of *N*-alkylaniline derivatives was achieved [68]. For this process, the authors used a bulky titanium catalyst, by which a desirable regioselectivity could be achieved (Scheme 7B and C). The obtained *N*-substituted anilines resemble some important compounds already known for their biological activities, such as antimalarial (**17**) [69] and anticancer (**18**) [70–71] properties (Scheme 7A).

**Scheme 8:**
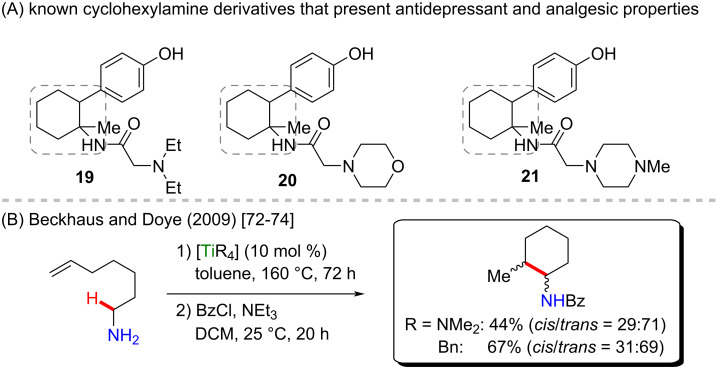
(A) Known biological activities related to cyclohexylamine derivatives; (B) an example of an intramolecular titanium-catalyzed C–H hydroaminoalkylation of alkenes.

Beckhaus and Doye reported cyclization processes mediated by titanium catalysis, that led to pyridinone derivatives or external cycloamines via an intramolecular titanium-catalyzed C–H hydroaminoalkylation [72–74]. In these works, two titanium catalysts were studied, tetrakis(dimethylamino)titanium [Ti(NMe_2_)_4_] and tetrabenzyltitanium [TiBn_4_], and it was observed that a better stereoselectivity was achieved by using the first catalyst while a better yield was obtained when the second catalyst was applied (Scheme 8B). Although the authors did not explore any possible biological activity of the obtained products, some compounds bearing an *N*-substituted cyclohexylamine moiety are known to present antidepressant and analgesic activities (e.g., compounds **19**, **20**, and **21**, Scheme 8A) [75]. Further studies could reveal other notable biological activities, thus justifying the need to develop new and accessible titanium catalysts.

As it can be observed from the previous cited works, titanium is by far one of the least explored metal in the field. Since it is a widely accessible and considerably cheap metal, it would be highly suitable for the development of methodologies for the synthesis of biologically active molecules. Based on the examples mentioned above, there is a specific preference for studies of amine derivatives. However, different functional groups that may go well along with titanium catalysis need still to be explored.

### Vanadium-catalyzed C–H activation

Vanadium is the twentieth most abundant element and the sixth most abundant transition metal in Earth’s crust. Rarely encountered in its metallic form, vanadium exists in oxidation states ranging from +5 to −3, including the four adjacent states +2 to +5 in aqueous solutions, and usually presents 4, 5 or 6 coordination numbers. The V(II) and V(V) species are reducing and oxidizing agents, respectively, whereas V(IV) is often encountered, mainly in the form of dioxovanadium ion VO^2+^ center [76].

Vanadium-based compounds have been reported to mediate the oxidation of alkanes to alcohols and ketones [76]. The reactions are usually mediated by V(V) and V(IV)-oxo-peroxo complexes, which are produced in situ from vanadium-oxo and dioxo precatalysts in the presence of oxidants, such as H_2_O_2_, O_2_, and *tert*-butyl hydroperoxide (TBHP) [76–82]. Inorganic acids and chelating and non-chelating carboxylic acids have been used as additives in these reactions and are suggested to act as ligands, assist proton transfer and promote the formation of oligovanadates by decreasing the pH value of the solution.

The mechanisms of some vanadium-mediated oxidation reactions of alkanes have been studied, most of them providing evidence for the involvement of radical species and a few suggesting non-radical pathways in the presence of a Lewis acid or oligovanadate complexes in solution [77,82–90]. Because most of the reactions are not likely to occur through either a direct metal-mediated C–H activation involving carbon–metal bond or a mechanism involving the usual metal-mediated coupling pathways comprising oxidative addition, transmetalation and reductive elimination steps, they are beyond the scope of this review and will not be extensively covered herein.

**Scheme 9:**
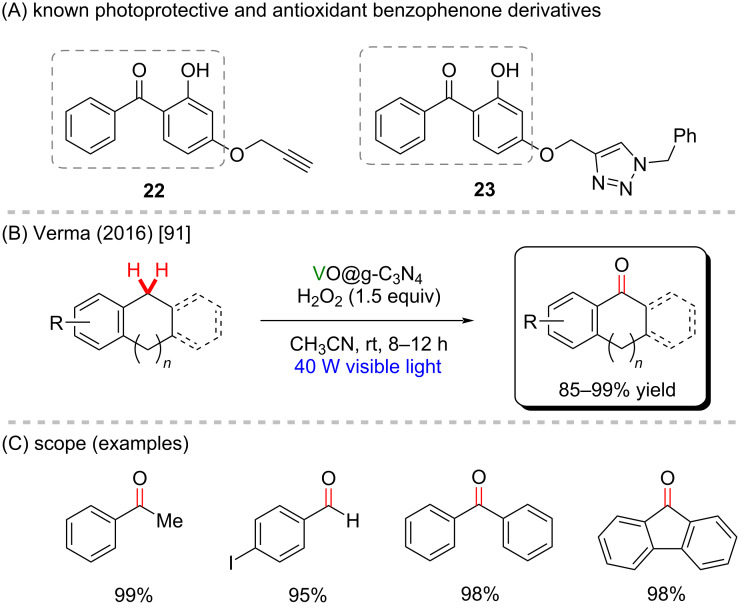
(A) Known biologically active benzophenone derivatives; (B and C) photocatalytic oxidation of benzylic C–H bonds mediated by supported vanadium.

Homogeneous and heterogeneous vanadium-based catalysts have been employed to obtain alcohols and carbonyl compounds through oxidation, including VOSO_4_, Na(VO_3_), VO(acac)_2_, VOX_3_, among others. Obtaining ketones and aldehydes from hydrocarbon compounds through vanadium-mediated activation of C(sp^3^)–H bonds in a benzylic position has been selectively feasible without activating a C(sp^2^)–H bond in the arene moiety. Verma and co-workers [91] have reported the use of VO(acac)_2_ immobilized over graphitic carbon nitride (VO@gC_3_N_4_) under visible light irradiation to perform a photocatalytic C–H activation of arene methides and derivatives. Using H_2_O_2_ as an oxidant agent, ketones and aldehydes were obtained from hydrocarbons with high yields (85–99%, Scheme 9B and 9C). Although the authors did not explore the biological effects of the obtained products, this class of molecules resembles the basic structure of several important biologically active molecules (**22** and **23**) (Scheme 9A) [92].

Interestingly, the same conditions could be used for benzene hydroxylation to obtain phenol but were ineffective with benzene rings bearing either electron-donating or electron-withdrawing substituents. Notably, the catalyst could be reused five times to oxidize ethylbenzene without significant loss of activity and metal leaching. The authors have suggested a mechanism for the reaction involving radical species bearing a benzylic carbon–vanadium bond.

**Scheme 10:**
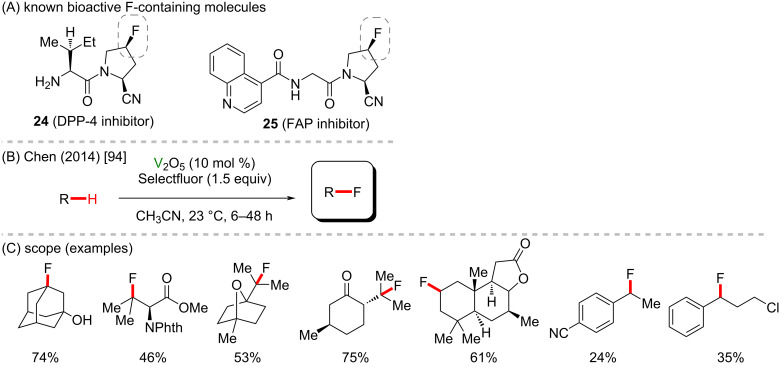
(A) Known bioactive fluorine-containing compounds; (B and C) vanadium-mediated C(sp^3^)–H fluorination.

Fluorine presents unique features and may lead to essential changes in the structural and physicochemical properties of a compound, thus affecting its pharmacodynamic and pharmacokinetic profile. Consequently, fluorination methods are particularly useful in the synthesis of bioactive substances, including marketed drugs (**24** and **25**) (Scheme 10A) [93]. In addition to oxygen insertion, vanadium use has also been reported for the direct C(sp^3^)–H fluorination. Chen and co-workers [94] described a fluorination method employing Selectfluor as fluorine source and the commercially available V_2_O_3_ to give fluorine-containing compounds under mild conditions and with moderate to good yields (Scheme 10B,C). The reaction showed to be chemoselective, maintaining good yields with compounds bearing varied functional groups, whereas low yields were observed for benzylic fluorination. Preliminary mechanistic studies suggested the C–H abstraction to be the rate-determining step and the high oxygen sensitivity of the reaction suggested it goes through a radical pathway.

Similar to the oxidation of alkanes to give alcohols and carbonyl compounds, vanadium complexes have been reported to mediate the hydroxylation of arenes, including the obtaining of phenol from benzene. However, most mechanistic studies provided evidence for radical pathways involving vanadium-peroxo species [76], with a few exceptions [95].

**Scheme 11:**
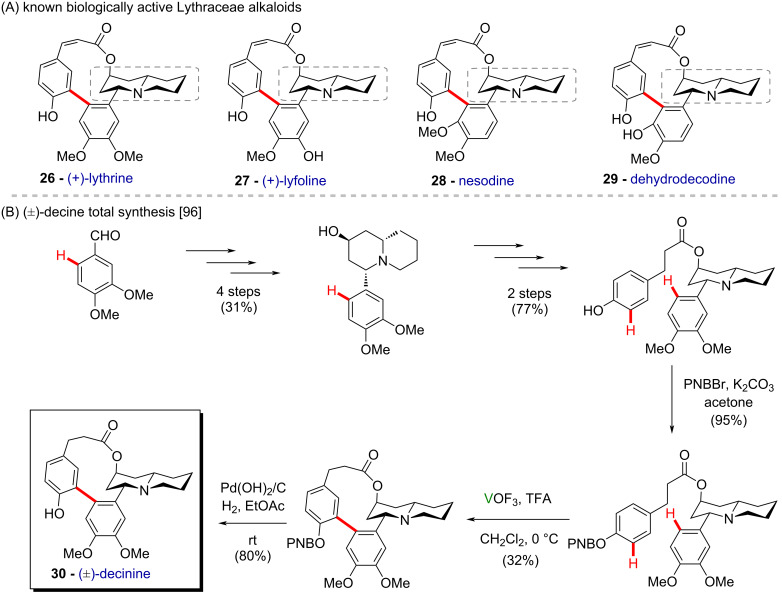
(A) Known biologically active Lythraceae alkaloids; (B) synthesis of (±)-decinine (**30**).

Vanadium-based catalysts have been employed in carbon–carbon bond formation reactions, such as arene couplings, thereby proving especially useful in the synthesis of bioactive compounds, including natural compounds and biaryl chiral auxiliaries. Also, the oxidative coupling of phenolic substrates has been reported to be mediated by vanadium complexes such as VCl_4_, VOCl_3_, and VOF_3_, among others. For instance, an intramolecular coupling of phenolic moieties using VOF_3_ has been reported as a final step in the synthesis of the bioactive natural macrolactone (±)-decinine (**30**) (Scheme 11B) [96]. These compounds (**26**–**29**) derive from the class of Lythraceae alkaloids (Scheme 11A), extracted from *Heimia salicifolia*, and present valuable pharmacological properties, such as antisyphilitic, sudoritic, antipyretic, laxative, and diuretic activities [97]. Trifluoroacetic acid was used as an additive, being suggested to avoid oxidation of the amine moiety by the formation of the corresponding ammonium salt.

**Scheme 12:**
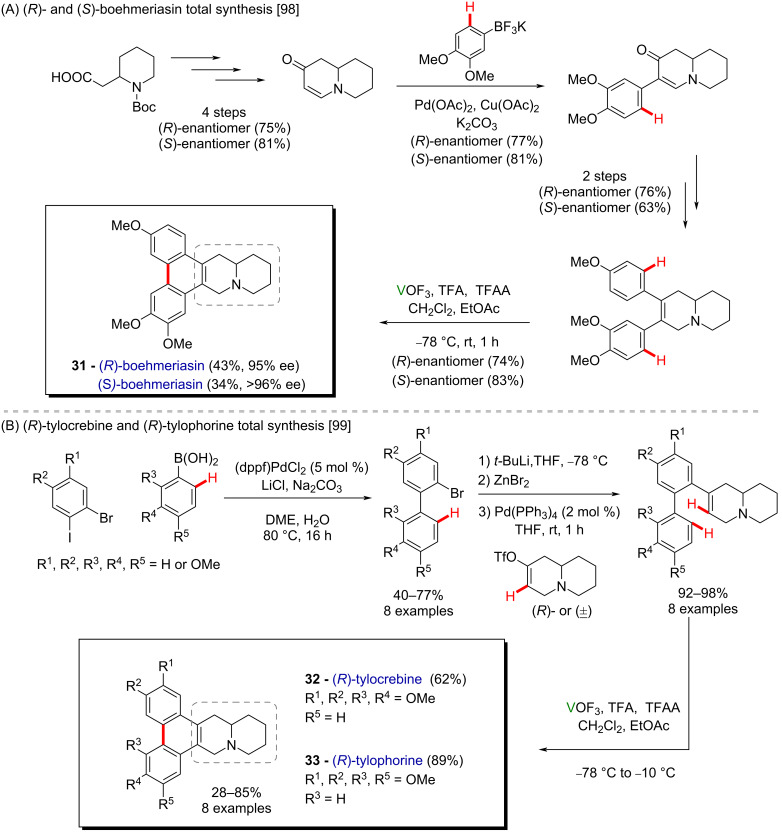
(A) Synthesis of (*R*)- and (*S*)-boehmeriasin (**31**); (B) synthesis of phenanthroindolizidines by vanadium-mediated aryl–alkene coupling.

Similar conditions have been reported for an alternative synthesis of both enantiomers of the antitumor phenanthroindolizidine alkaloid boehmeriasin A (**31**) (Scheme 12A) [98], and phenanthroindolizidines through an intramolecular oxidative aryl–alkene coupling (Scheme 12B) [99], which is a far less common transformation in organic synthesis. This approach was employed to synthesize eight phenanthroindolizidines, including (*R*)-tylocrebrine (**32**) and (*R*)-tylophorine (**33**), which were found to display antiproliferative activity in the nanomolar range against human colorectal carcinoma, human breast carcinoma and drug-resistant human ovarian adenocarcinoma cell lines.

**Scheme 13:**
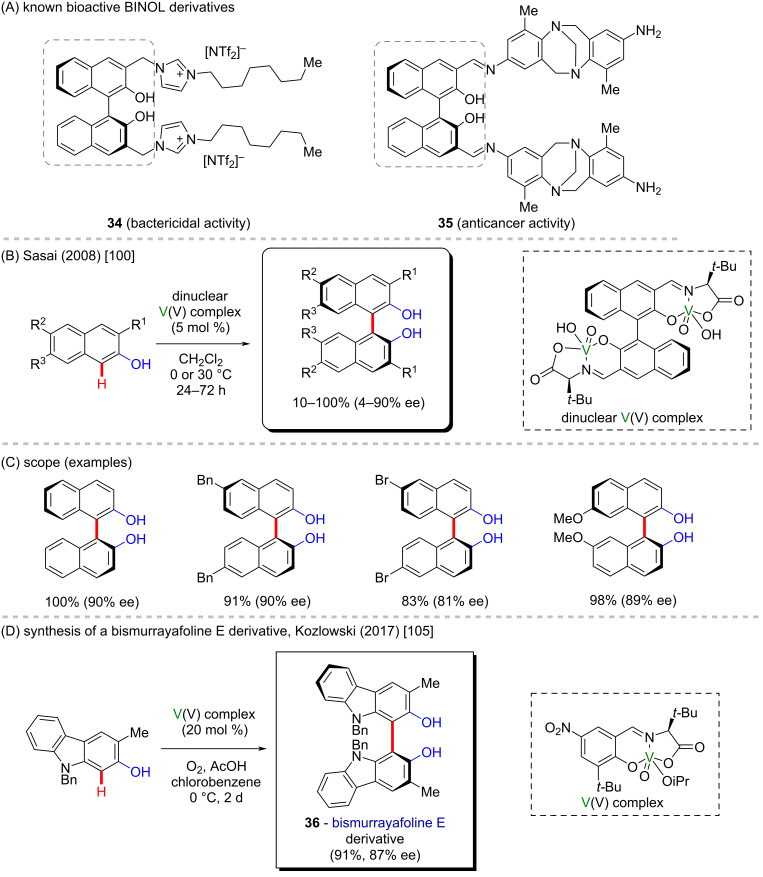
(A) Known bioactive BINOL derivatives; (B and C) vanadium-mediated oxidative coupling of 2-naphthols; (D) synthesis of a bismurrayafoline E derivative (**36**).

Methods for the oxidative homocoupling of phenolic compounds to produce the corresponding biaryl products with high enantiopurity using vanadium chelated with chiral ligands, such as tridentate asymmetric imine ligands, have been reported. For instance, (*S*)-binol derivatives could be successfully prepared from 2-naphthols using a dimeric vanadium complex (Scheme 13B and C) [100]. Binols have been reported to present bactericidal (**34**) [101] and anticancer activities (**35**) [102] (Scheme 13A). In this work, the use of a dinuclear catalyst was found to strikingly increase the reaction rate, presumably by reducing entropic costs associated with bringing two molecules of substrate together. In addition, the high enantiopurity was ascribed to a chiral environment that presents three elements of asymmetry. Other examples of vanadium-mediated oxidative homocouplings of phenolic substrates include regioselective and asymmetric homocoupling of phenols and 2-hydroxycarbazoles [103–104].

Notably, Kozlowsky and co-workers were the first who reported a method for the vanadium-based asymmetric coupling of phenols and 2-hydroxycarbazoles [105] that allowed the synthesis of a wide range of chiral biphenols and bicarbazoles. The use of a vanadium complex with less electron-rich ligands along with the addition of Brønsted and Lewis acids, such as acetic acid and LiCl, was suggested to account for the suitability of the method for the coupling of oxidatively resistant substrates. The usefulness of this method was demonstrated in the preparation of the bicarbazole moiety present in the natural product bismurrayafoline E (**36**) [105], an alkaloid found in the leaves of *Murraya koeniggi* (Scheme 13D) [106].

**Scheme 14:**
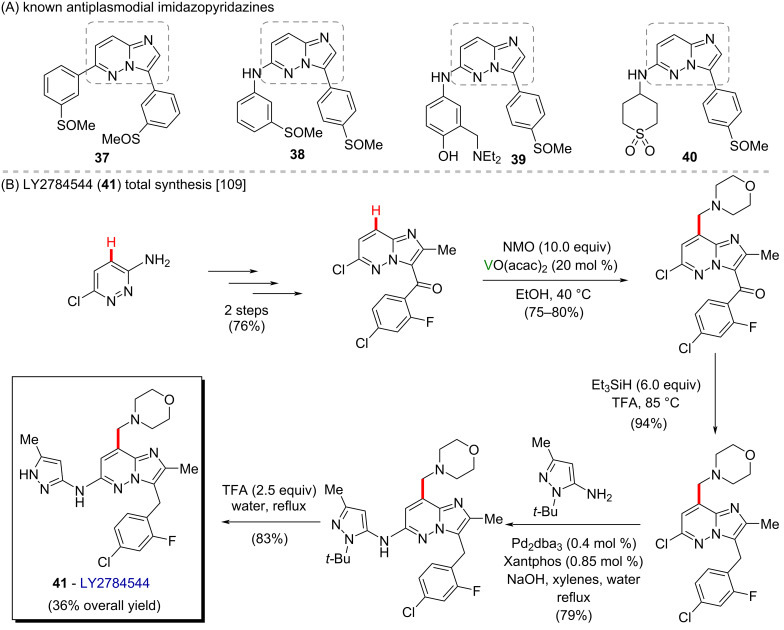
(A) Known antiplasmodial imidazopyridazines; (B) practical synthesis of **41**.

The oxidative formation of carbon–carbon bonds mediated by vanadium has been reported as a method for the aminomethylation of arenes and heteroarenes. The so far described methods include V_2_O_5_ and VO(acac)_2_ used for the alkylation of 2-naphthol and nitrogen-containing heteroaromatic moieties containing *N*-methylmorpholine-*N*-oxide, tetrahydroisoquinolines, and *N*,*N*-dimethylacetamide [107–113]. The mechanisms involving oxidation of the amine mediated by vanadium to give iminium ions, followed by a nucleophilic attack of the heteroaromatic ring, have been suggested for most reactions. Evidence comes from the observed regioselectivity and the tolerance of the reactions to radical scavengers, which are in accordance with the occurrence of a heteroaromatic electrophilic substitution and a non-radical pathway. An aminomethylation of the heteroaromatic ring with *N*-methylmorpholine-*N*-oxide catalyzed by VO(aca)_2_ reported by Mitchell and co-workers [109], however, was found to undergo with a regioselective outcome incompatible to an electrophilic aromatic substitution reaction. The substrate failed to give the same product when subjected to alkylation with the isolated putative iminium ion intermediate. The authors then suggested the reaction took place through a radical mechanism instead. This vanadium-mediated aminoalkylation reaction was found to be a helpful strategy in the synthesis of compound LY2784544 (**41**), a potent inhibitor of Janus kinase 2 that is under clinical trials for the management of myeloproliferative disorders (Scheme 14B) [109–110]. Beyond that, this class of molecules (imidazopyridazines) are known to present a potent antiplasmodial activity (**37**–**40**) (Scheme 14A) [114]. The use of vanadium-mediated aminoalkylation led to the introduction of a morpholinomethyl moiety into the heteroaromatic ring in a single step instead of the 5 steps required in the previously used route, thus significantly shortening the synthetic route, and increasing the overall yield. The resulting eight-step synthesis could be scaled to produce more than 100 kg of compound **41** [109].

Based on what was presented so far, vanadium catalysis is mainly applied as one of the steps involved in a total synthesis that usually leads to the formation of a valuable biologically active substance. This fact clarifies the high importance of further studies and evaluations in this field. With this information in mind and the fact that vanadium catalysis is also considerably accessible, it is possible to expect that more complex and currently high-cost drugs may be obtained in a cheaper way leading to an easier access of currently expensive treatments to the general population.

### Chromium-catalyzed C–H activation

Chromium is a relatively abundant transition metal that has been used for oxidative reactions, including cross-coupling and carbon–carbon bond formation involving organochromium species generated from alkyl halides [115–116]. Whereas its toxicity has hindered the use of Cr(VI) in organic synthesis, the less toxic Cr(III) and Cr(II) salts have been exploited as plausible catalysts in organic synthesis [117–118]. A good example is a redox-neutral reaction for the allylation of aldehydes promoted by a dual catalytic system comprising CrCl_3_ and an iridium-based photocatalyst that was recently developed by Schwarz and co-workers [119]. Similar conditions were further employed to synthesize monoprotected 1,2-homoallylic diols from aldehydes and silyl and alkyl enol ethers as the allylic counterpart [120].

**Scheme 15:**
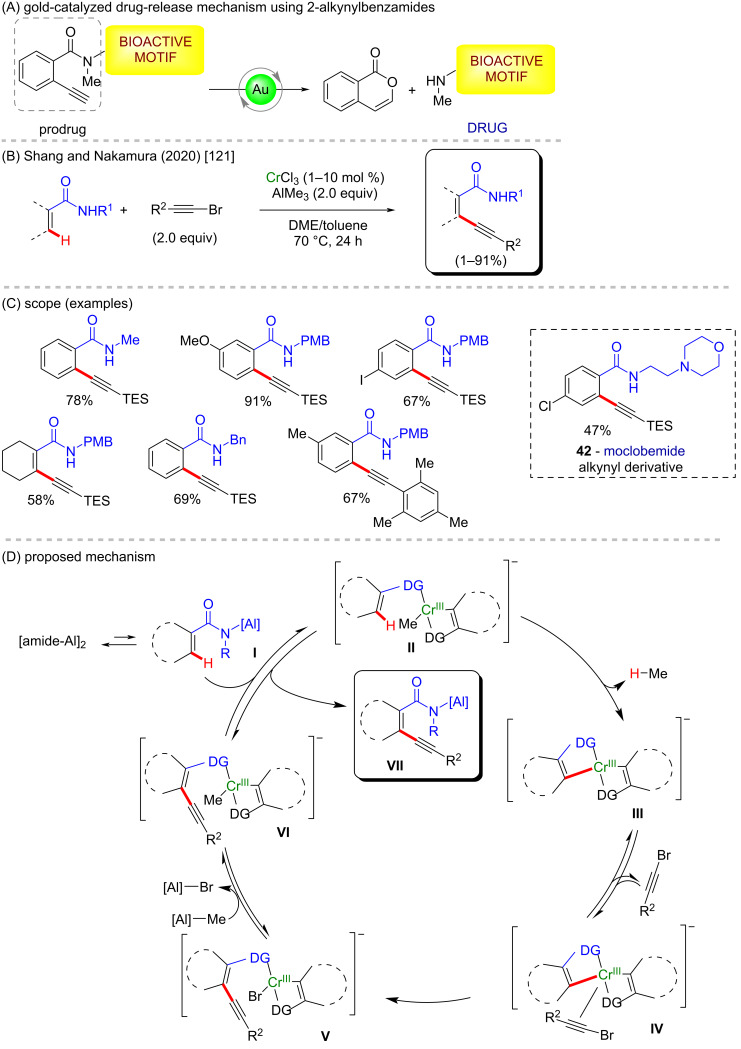
(A) Gold-catalyzed drug-release mechanism using 2-alkynylbenzamides; (B and C) chromium-mediated alkynylation of carboxamides; (D) proposed mechanism.

In the field of C–H activation studies, not many examples have been described in the literature so far, especially towards the synthesis of biologically active compounds. However, an interesting example of a C(sp^2^)–H activation reaction promoted by Cr(III) salts and AlMe_3_ as a base for the regioselective *ortho* functionalization of aromatic secondary amides has been recently reported [121]. The reaction is performed with 1–2 mol % of CrCl_3_ or Cr(aca)_3_, a stoichiometric amount of AlMe_3_ and bromoalkynes (Scheme 15B), allyl bromide or 1,4-dihydro-1,4-epoxynaphthalene as electrophiles. Several 2-alkynylbenzamide products were achieved in good yields, including a derivate of moclobemide (**42**), a drug used to treat depression and anxiety (Scheme 15C) [122]. It is worth mentioning here that this class of molecules can be used as effective anticancer drug carriers, which via a gold catalysis can be triggered to release the drug on specific sites of the target cell in a controlled manner (Scheme 15A) [123]. A proposed catalytic cycle based on kinetic isotopic effect and kinetics data is illustrated in Scheme 15D. At the beginning of the reaction, intermediate **III** can be formed from **I** and the Cr(III) salt to start the cycle, thereby providing intermediate **VI**. The latter then undergoes a ligand exchange with **I** to give the product and the key intermediate **II**. It is noteworthy that the secondary amide works both as the substrate and the ligand for the metal center, so that no additional ligand is required. The method presents a broad scope regarding the amide substrate. It gives moderate to excellent yields for heteroaromatic and aromatic carboxamides bearing electron-donating and electron-withdrawing substituents, whereas substrates with *ortho*-substituents were found to be unreactive, probably due to steric hindrance.

**Scheme 16:**
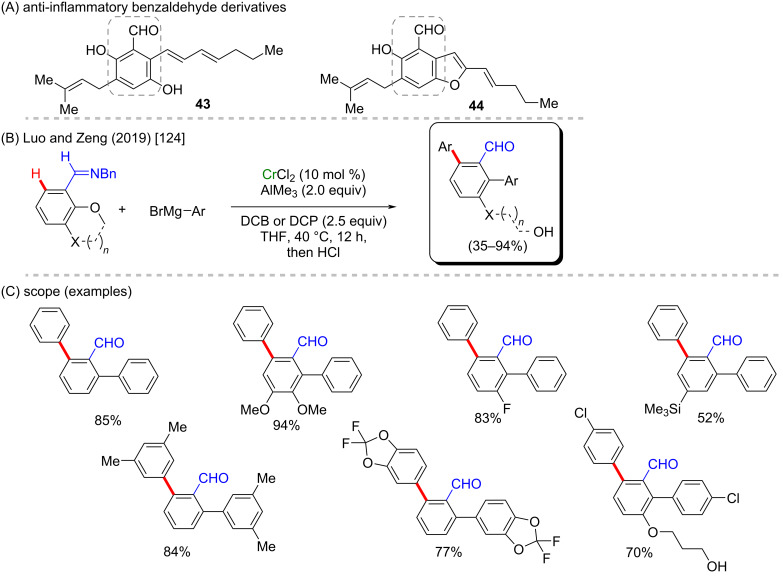
(A) Examples of anti-inflammatory benzaldehyde derivatives; (B and C) chromium-mediated difunctionalization of arenes.

The one-pot difunctionalization of arenes involving a sequential C–O cleavage and C(sp^2^)–H activation mediated by chromium was recently reported by Luo and Zeng [124]. The reaction allows an *ortho*-directed diarylation of *o*-methoxybenzaldehyde imine derivatives with phenyl Grignard reagents as coupling partners. As the catalyst CrCl_2_ is used and either 2,3-dichlorobutane (DCB) or 1,2-dichloropropane (DCP) are used as oxidant to give 2,5-diarylbenzaldehyde after imine hydrolysis (Scheme 16B). Although benzaldehyde is a basic structure, it is present in compounds that have a significant anti-inflammatory activity (**43** and **44**) (Scheme 16A) [125]. The introduction of two distinct aryl moieties into arenes was also shown to be feasible through the sequential functionalization with two different Grignard reagents. For this purpose, the reaction was kept at room temperature to avoid the difunctionalization, and the oxidant was added only in the second step. Following this route, six examples were obtained with moderate yields (56–70%, Scheme 16C).

Although the use of chromium catalysis is still considered challenging due to the high toxicity of some chromium species, based on the above cited examples, by using this metal valuable biologically active molecules can be safely obtained. The correct, judicious, and optimized application of chromium-based catalysts can lead to the easier access of several drugs. Further studies in this area could help expanding the currently available methods in organic synthesis.

### Manganese-catalyzed C–H activation

**Scheme 17:**
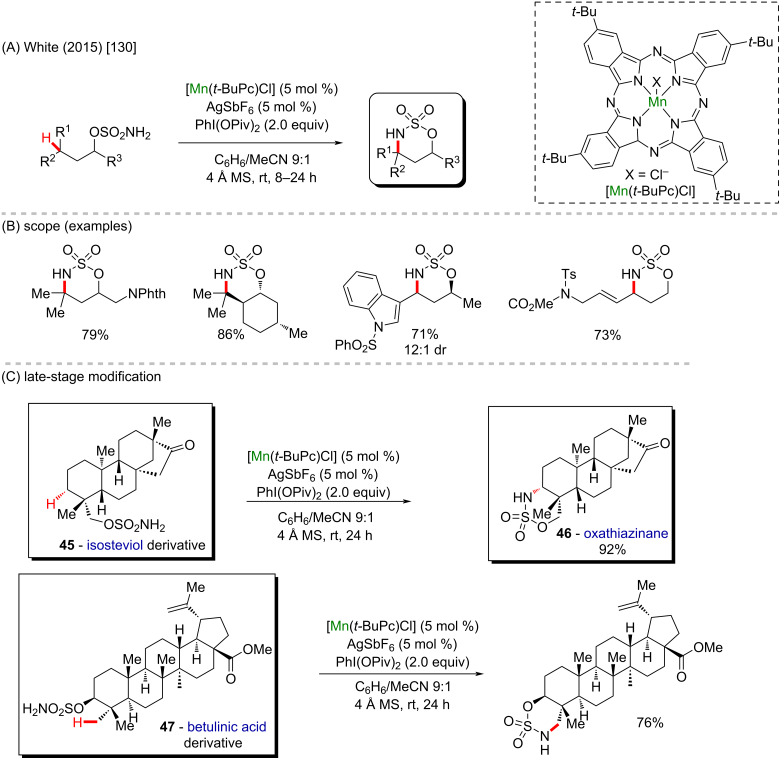
(A and B) Manganese-catalyzed chemoselective intramolecular C(sp^3^)–H amination; (C) late-stage modification of an isosteviol derivative (**45**) and a betulinic acid derivative (**47**).

Manganese is the twelfth most abundant element in the Earth’s crust and the third most abundant transition metal after iron and titanium [126]. The valence electron configuration of elemental manganese is 3d^5^4s^2^ with a high redox potential due to the high number of available oxidation states (−3 to +7), allowing the formation of compounds with a coordination number of up to 7 [127]. These properties associated with a low toxicity and low cost make manganese a metal with great potential in organometallic chemistry and catalysis [128]. The first example of a stoichiometric manganese-mediated C–H activation, reported by Stone, Bruce, and co-workers (1970) [129], was an *ortho*-metalation in azobenzenes. In recent years, with the expansion of the C–H activation field, manganese catalysts have been applied in some very sophisticated protocols including several examples reported by the White group. In 2015, White and co-workers reported a new catalytic method using manganese *tert*-butylphthalocyanine [Mn(*t*-BuPc)Cl] for the chemoselective intramolecular amination of various C(sp^3^)–H bond types like benzylic, allylic, 3°, 2°, and 1° aliphatic, using unsubstituted linear sulfamate esters (Scheme 17A and B) [130]. This method is also compatible with substrates containing adjacent substituents like protected amines and tolerates the presence of electron-withdrawing groups as well as α-substituted alkynes. The authors also succesfully applied the method toward a C-substituted indol, which is a particularly useful heteroaromatic motif in medicinal chemistry, being encountered in many bioactive compounds. The corresponding product was obtained with good yield and high diastereoisomeric ratio. Following these results, the late-stage diversification using the [Mn(*t*-BuPc)Cl] catalyst was applied to more complex natural compounds such as the isosteviol derivative **45** and the betulinic acid derivative **47**, which underwent conversion in good and high yields and with high chemoselectivity. The functionalized isosteviol derivate furnished a useful and versatile oxathiazinane (**46**). The betulinic acid-derived sulfamate ester preferentially underwent amination at the γ primary C–H bond of the equatorial C23 methyl group with high site- and diastereoselectivity to furnish the oxathiazinane derivative in 76% yield (Scheme 17C).

**Scheme 18:**
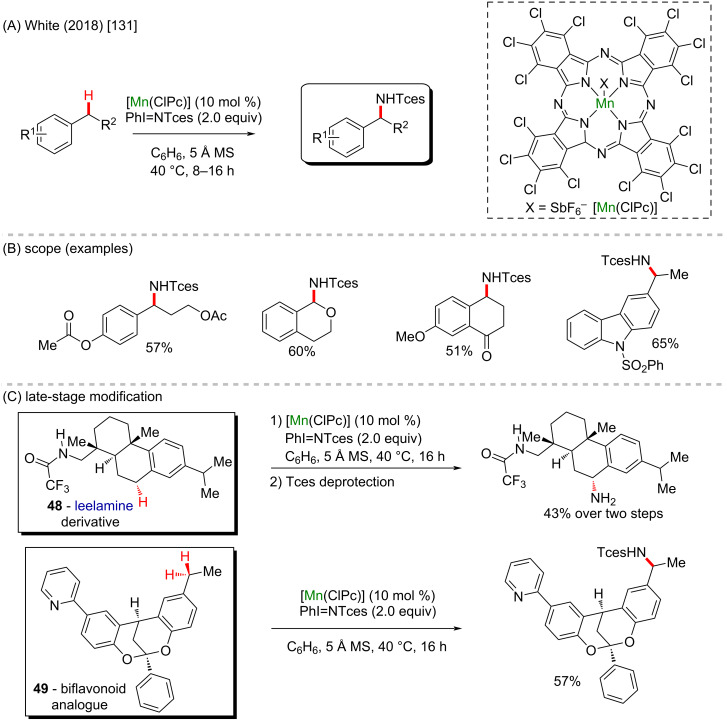
(A and B) Manganese-catalyzed C(sp^3^)–H amination; (C) late-stage modification of a leelamine derivative (**48**) and a biflavonoid analogue (**49**).

The White group also reported a series of manganese-catalyzed intermolecular benzylic C(sp^3^)–H amination reactions using 2,2,2-trichloroethyl sulfamate (TcesNH_2_) and PhI(OPiv)_2_ as the oxidant (Scheme 18A and 18B) [131]. The manganese perchlorophthalocyanine [Mn^III^(ClPc)Cl] catalyst enabled a highly site-selective and wide functional-group tolerant reaction. To test this protocol versatility over biologically relevant scaffolds, a COCF_3_-leelamine analogue **48** with two sterically differentiated benzylic sites was selectively aminated at the secondary benzylic site. After Tces deprotection, the free amine derivative was produced in 43% overall yield. The 2,8-dioxabicyclo[3.3.1]nonane skeleton is contained in biflavonoids and was shown to exhibit many medicinal activities like antiviral, anti-inflammatory and antitumor properties. The bicyclic compound **49** was aminated at the benzylic position in 57% yield (1:1 dr, Scheme 18C).

**Scheme 19:**
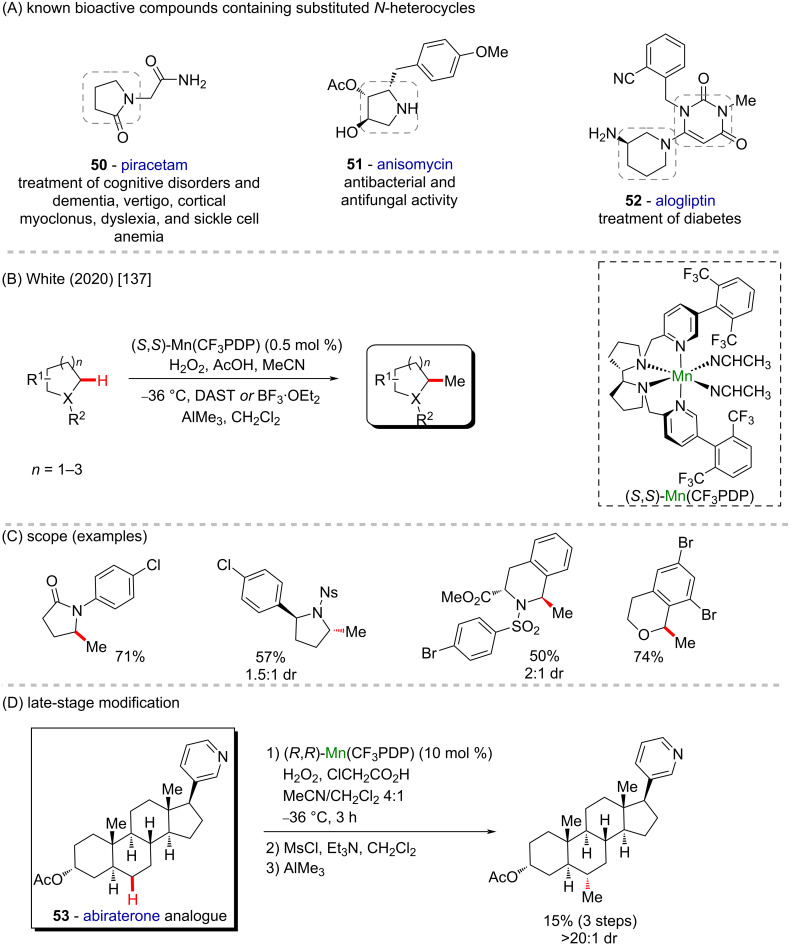
(A) Known bioactive compounds containing substituted *N*-heterocycles; (B and C) manganese-catalyzed oxidative C(sp^3^)–H methylation; (D) late-stage modification of an abiraterone analogue **53**.

**Scheme 20:**
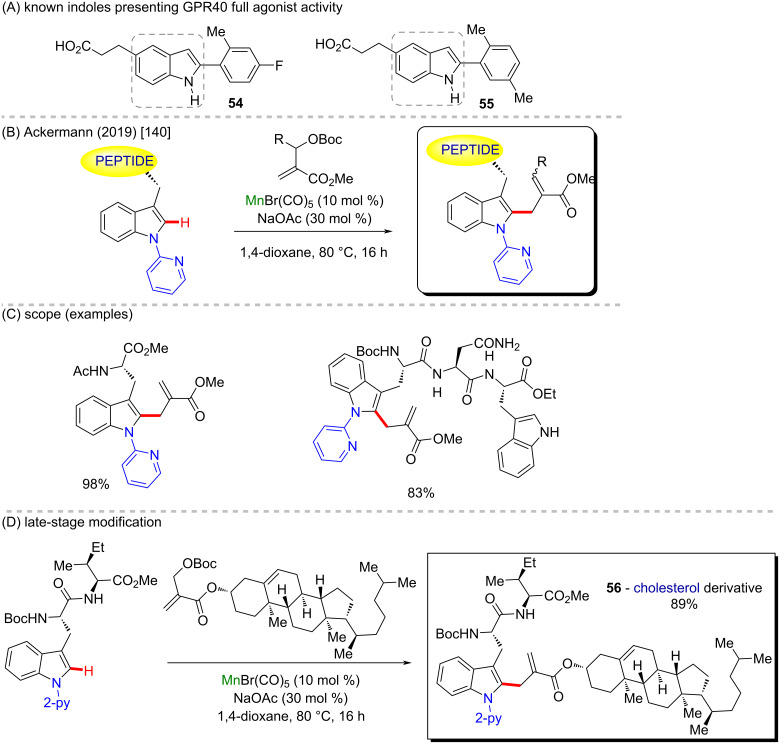
(A) Known indoles that present GPR40 full agonist activity; (B and C) manganese-catalyzed C–H alkylation; (D) late-stage modification furnishing **56**, using a cholesterol derivative as coupling partner.

Nitrogen-containing heterocycles are present in several valuable bioactive compounds, such as piracetam (**50**) [132], anisomycin (**51**) [133], and alogliptin (**52**) [134] (Scheme 20A). Sometimes better biological results are observed after simple structural modifications, and the introduction of methyl groups has an attractive effect on the properties of medicinal compounds [135]. However, the selective late-stage methylation has a limited scope due to the lack of suitable methodologies [136]. The insertion of methyl groups at positions adjacent to heteroatoms often has a further effect improvement, however, it is even more challenging. In front of this, White and co-workers (2020) adopted a strategy consisting of an initial hydroxylation of the C(sp^3^)–H bonds adjacent to N- or O-heteroatoms followed by a methylation step (Scheme 19B and C) [137]. The hydroxylation of positions next to heteroatoms leads to hemiaminals and hemiacetals which typically promotes an overoxidation to the corresponding carbonyl compound. The sterically hindered catalyst Mn(CF_3_PDP)(MeCN)_2_(SbF_6_)_2_ (where CF_3_PDP is 1,1′-bis((5-(2,6-bis(trifluoromethyl)phenyl)pyridin-2-yl)methyl)-2,2′-bipyrrolidine) controls the site- and chemoselectivity in the hydroxylating step of the methylene C(sp^3^)–H bond while milder oxidation conditions help to increase the chemoselectivity. The methylation step is accomplished using a modestly nucleophilic organoaluminium reagent (AlMe_3_) to activate the hemiaminal/hemiacetal avoiding undesirable elimination to the enamine, or attack at other electrophilic sites in complex substrates. The presence of a fluorine source like diethylaminosulfur trifluoride (DAST) or the Lewis acid boron trifluoride diethyl etherate (BF_3_·OEt_2_) result in the formation of reactive iminium or oxonium species in the methylation step. Abiraterone acetate (**53**) is a drug used in cancer treatment [138]. The Mn-catalyzed methylation of an abiraterone analogue was achieved by replacing the fluorination step by mesylation in 15% of overall yield and only a single diastereoisomer was observed (Scheme 19D). In carbocyclic substrates the displacement of a C–F bond or ionization with a Lewis acid is difficult, but mesylates are stable and suitable for AlMe_3_ activation.

As already mentioned, indoles are an important class of molecules with potential antidiabetic properties since they can act as GPR40 full agonists (**54** and **55**, Scheme 20A) [139]. Ackermann and co-workers presented a manganese(I)-catalyzed C–H allylation, installing α,β-unsaturated esters in peptide analogues bearing indole motifs (Scheme 22B) [140]. Starting with tryptophan, the enantioselective allylation reaction afforded the product (98% yield) (Scheme 20C). More complex structures like dipeptides and substrates containing multiple Lewis-basic functionalities also presented good yields and chemoselectivities with this protocol. The robust allylation reaction was tested in even more complex structures, including steroid-containing substrates, and its high efficiency makes it an excellent methodology for late-stage modifications (Scheme 20D).

**Scheme 21:**
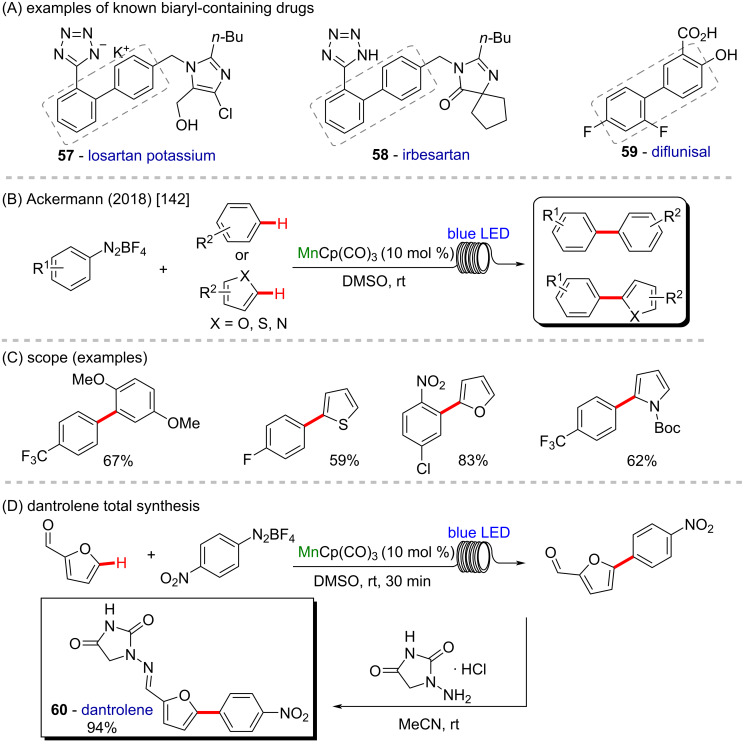
(A) Examples of known biaryl-containing drugs; (B and C) manganese-catalyzed C–H arylation through continuous visible light photo-flow reaction; (D) dantrolene (**60**) total synthesis.

Biaryl structures are present in several important drugs currently commercially available (**57**, **58**, and **59**, Scheme 21A) [141] and synthetic methods to achieve the aryl–aryl connection are crucial. In 2018, Ackermann and co-workers described a novel room temperature C–H arylation by using a continuous visible light photo-flow technique, allied with a manganese photocatalyst CpMn(CO)_3_ [142]. The new flow protocol enabled the synthesis of several arene- and heterocyclic-based compounds in excellent yields and short reaction times (Scheme 21B and C). The robustness of the manganese-catalyzed photo-flow reaction was demonstrated by a gram-scale preparation of the key intermediate in the synthesis of the pharmaceutical compound dantrolene (**60**) in high yields (Scheme 21D).

**Scheme 22:**
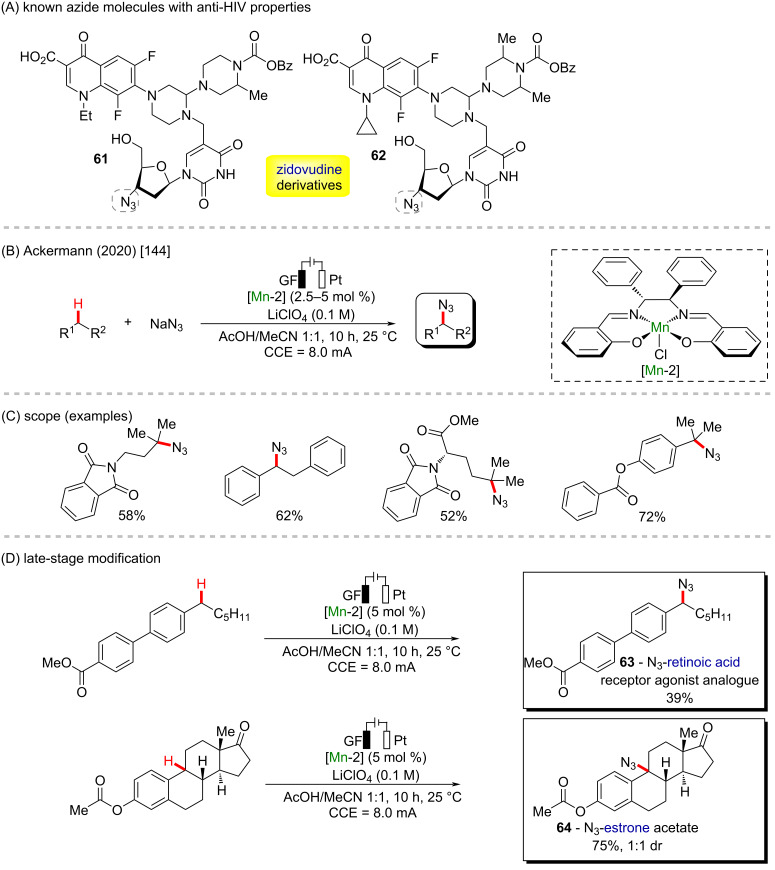
(A) Known zidovudine derivatives with potent anti-HIV properties; (B and C) manganese-catalyzed C–H organic electrosynthesis; (D) late-stage modification.

The azide group is another powerful organic function present for example in zidovudine, a worldwide known anti-HIV drug, but also in its derivatives (**61** and **62**) (Scheme 22A), which can present 10-times higher activities against HIV replication [143]. In 2020 the same group reported the use of a manganese catalyst in the azidation of inert C(sp^3^)–H bonds using organic electrosynthesis in a straightforward procedure, enabling the azidation of a series of primary, secondary and tertiary alkyl moieties (Scheme 22B and C) [144]. In general, the new methodology proved to be resource-economic and straightforward, operating under mild conditions without the need of directing groups, using traceless electrons as sole redox reagents, presenting high scope and chemoselectivity. The robustness of the reaction was proved by the late-stage modification of pharmaceutically relevant compounds by promoting the azidation of a retinoic acid receptor agonist analogue **63** and an estrone acetate derivative **64** (Scheme 22D).

**Scheme 23:**
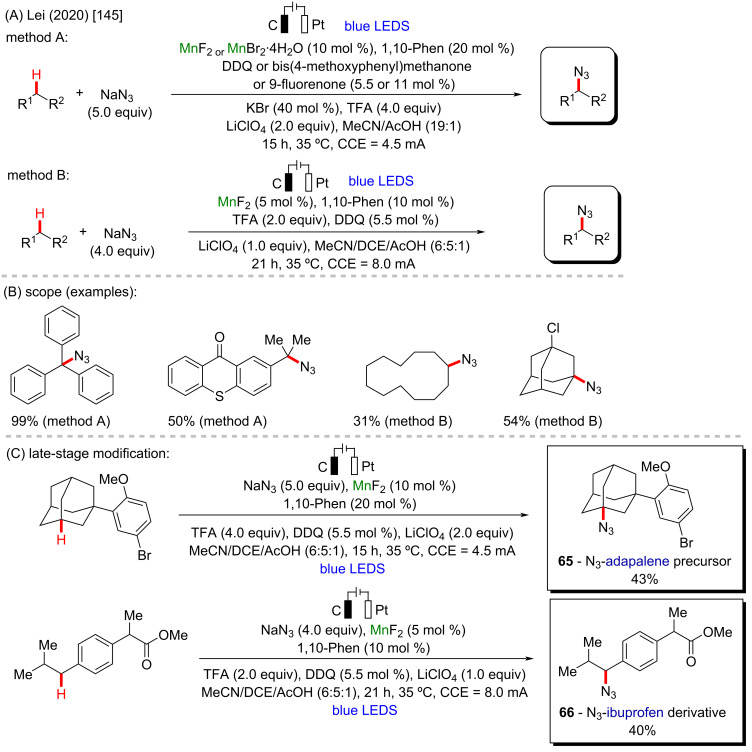
(A and B) Manganese-catalyzed C–H organic photo-electrosynthesis; (C) late-stage modification.

A seminal work involving manganese-catalyzed C–H organic electrosynthesis and photoredox catalysis was reported in the same year by Lei and co-workers, also regarding the azidation of alkyl scaffolds (Scheme 23A and B) [145]. The authors successfully applied a manganese salt in catalytic amounts, allied with the use of an electrical current in combination with blue LED lights and an organic photocatalyst (DDQ), affording azidated alkyl moieties in excellent yields and chemoselectivity through alkyl C–H bonds. The photo-electro methodology allowed the late-stage functionalization of valuable bioactive molecules, and the structures of a adapalene precursor (**65**) and ibuprofen derivative (**66**) were successfully azidated in moderate yields (Scheme 23C).

**Scheme 24:**
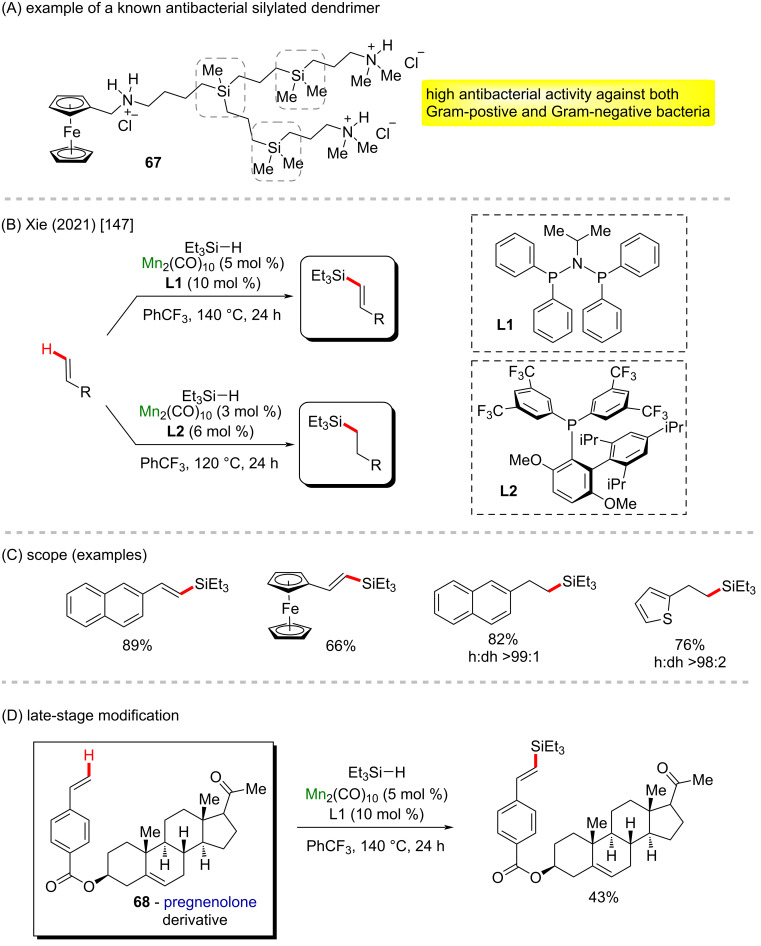
(A) Example of a known antibacterial silylated dendrimer; (B and C) manganese-catalyzed C–H silylation; (D) late-stage modification of the pregnenolone derivative **68**.

Some silylated compounds like **67** have proven to be efficient antibacterial compounds (Scheme 24A) [146]. In 2021, a divergent silylation of alkenes via a manganese-catalyzed C–H activation was reported by Xie et al. by using a ligand-tuned metalloradical reactivity strategy (Scheme 24B and C) [147]. Using Mn_2_(CO)_10_ as a catalyst precursor, the authors described the dehydrosilylation and hydrosilylation of alkenes to afford silylated alkanes and alkenes in excellent yields and stereoselectivity, depending on the phosphine-based ligand employed. The reaction proved to work through a redox-neutral path, being considered an atom-economical process, exhibiting a broad substrate scope and excellent functional group tolerance. It enabled the late-stage diversification of a complex molecule like a pregnenolone derivative (**68**, Scheme 24D).

As it can be seen, using either more complex or more simple manganese catalysis, it is possible to obtain a large variety of functionalized compounds, from arylated compounds to new azide substances. This is an important characteristic in the synthesis and evaluation of biologically active molecules, since using different methods, a large variety of potential compounds can be obtained and studied. Larger scopes increase the chance of finding plausible new drugs, with potent activities.

### Iron-catalyzed C–H activation

By mass, iron is the most abundant metal on Earth. Therefore, it has been and still is widely used in many fields, from ancient but still applicable appliance as feedstock to construct basic steel tools [148], to the most recent nanotechnology field [149]. Iron presents powerful catalyst properties [150–152], including applications in C–H activation reactions [153–155].

**Scheme 25:**
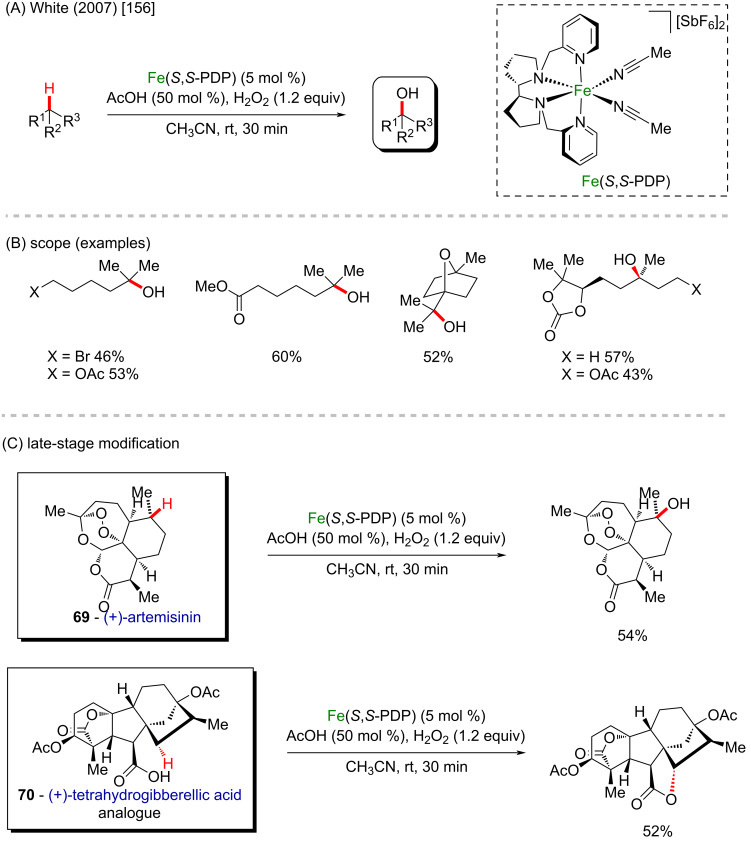
(A and B) Fe-based small molecule catalyst applied for selective aliphatic C–H oxidations; (C) late-stage modification of (+)-artemisinin (**69**) and a (+)-tetrahydrogibberellic acid analogue **70**.

In 2007, White and Chen reported a seminal work regarding predictably selective aliphatic C–H oxidations by using an iron-based small molecule catalyst and hydrogen peroxide as oxidizing agent (Scheme 25A and B) [156]. This pioneering methodology changed the way how complex molecules and pharmaceuticals are synthesized, by using the steric and electronic properties of the substrates to achieve selectivity, without the need for directing groups, presenting a broad scope and satisfactory yields. The late-stage modification of complex molecules like (+)-artemisinin (**69**) and a (+)-tetrahydrogibberellic acid analogue **70** could be rapidly achieved in moderate yields (Scheme 25C).

**Scheme 26:**
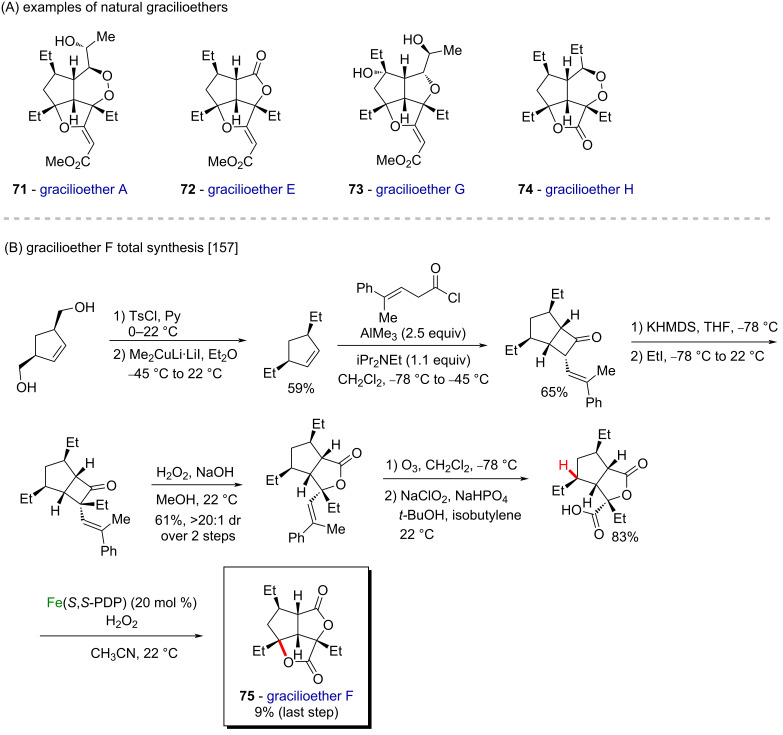
(A) Examples of naturally occurring gracilioethers; (B) the first total synthesis of gracilioether F (**75**) utilizing a C–H oxidation in the final step.

In 2014, Brown and Rasik employed White’s oxidation method as the final step in the first total synthesis of gracilioether F (**75**) [157], a natural polyketide with an unusual tricyclic core and five contiguous stereocenters, part of the family of gracilioethers **71–74** (Scheme 26A) extracted from the marine sponge *Plakinastrella mamillaris* [158]. The synthesis started with a cyclopentene derivative, obtained after two steps from a diol via tosylation/displacement strategy with Me_2_CuLi·LiI. Then, after a Lewis acid-promoted cycloaddition, the alkylation of the α-carbon atom followed by regioselective Baeyer–Villiger oxidation provided the target lactone in 61% yield over two steps. The final steps involved a one-pot ozonolysis with quenching under Pinnick oxidation conditions to afford the carboxylic acid derivative in 83% yield, followed by White’s selective C–H oxidation (Scheme 26B).

**Scheme 27:**
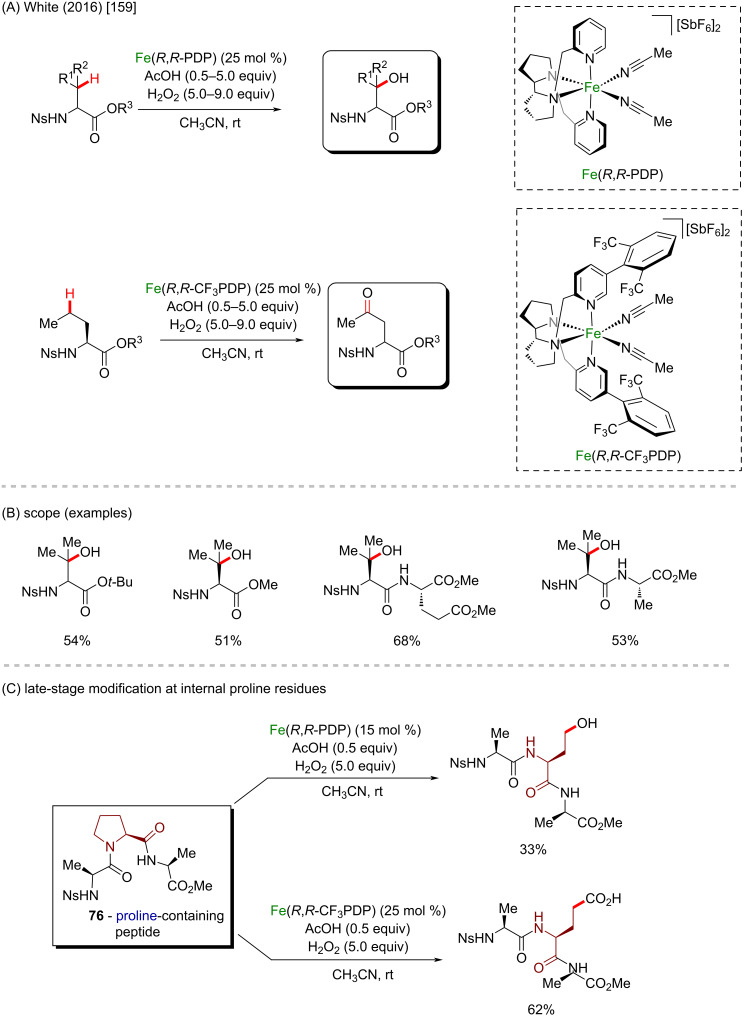
(A and B) Selective aliphatic C–H oxidation of amino acids; (C) late-stage modification of proline-containing tripeptide **76**.

White’s selective C–H oxidation was also applied in the late-stage modification of amino acids and peptides in 2016 (Scheme 27A and B) [159]. The methodology facilitated the targeted C–H oxidative modifications in amino acids and peptides concomitant with the preservation of the α-center chirality in good yields and broad scope regarding the number of amino acids and peptide scaffolds compatible with the transformation. The late-stage oxidation of proline **76** to 5-hydroxyproline furnished interesting intermediates, giving access to relevant motifs in peptide chemistry (Scheme 27C).

**Scheme 28:**
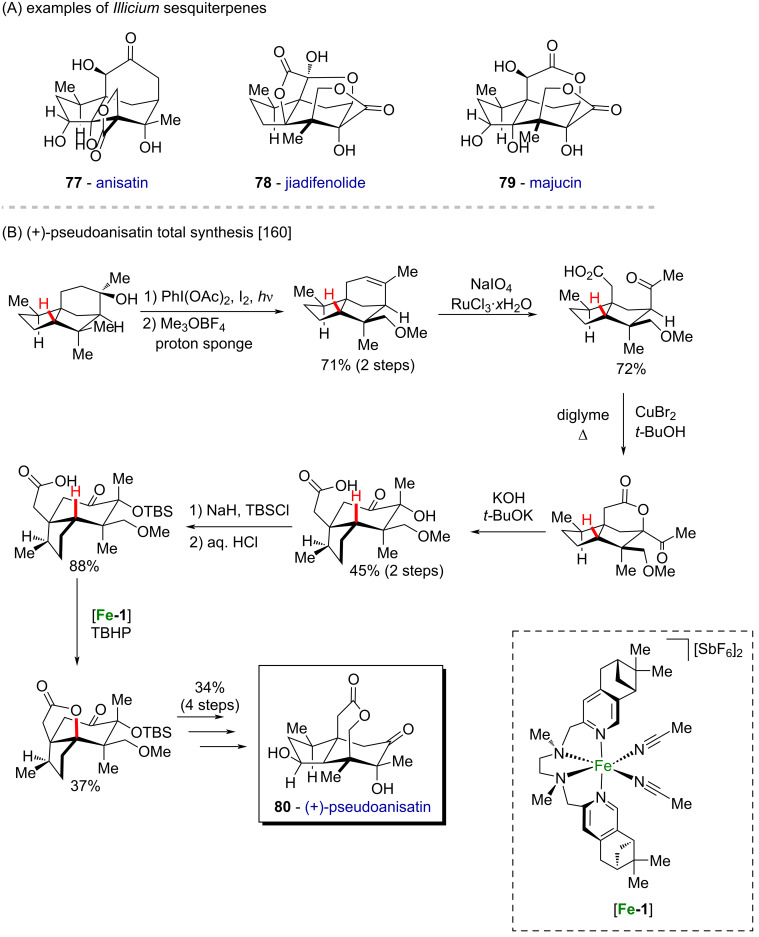
(A) Examples of *Illicium* sesquiterpenes; (B) first chemical synthesis of (+)-pseudoanisatin (**80**) in 12 steps.

Sesquiterpenes are known to present complex polycyclic structures with several chiral centers, and nowadays, several sesquiterpenes have been isolated from plants of the *Illicium* genus (**77**–**79**, Scheme 28A) [160]. (+)-Pseudoanisatin (**80**) is one example, and its first chemical synthesis was achieved in 12 steps by Maimone and collaborators in 2016 utilizing a straightforward site-selective C(sp^3^)−H bond functionalization strategy (Scheme 28B) [160]. Starting from the abundant feedstock chemical cedrol, oxidation of the *gem*-dimethyl group was achieved on a gram scale, with the formation of a strained tetrahydrofuran ring. The latter was methylated and eliminated via the action of Meerwein’s salt (Me_3_OBF_4_) and a mild base (proton sponge) to afford a methoxy cedrene derivative. Next, oxidative cleavage of the double bond using NaIO_4_/RuCl_3_·*x*H_2_O enabled a ring opening, followed by lactonization promoted by CuBr_2_ via an intramolecular acyloxylation. The 5,5-fused ring system was then converted to a 5,6-fused *seco*-prezizaane scaffold through an α-ketol rearrangement promoted by a strong base and after secondary alcohol protection with TBSCl, a Fe-catalyzed C–H activation reaction promoted a second lactonization to afford (+)-pseudoanisatin (Scheme 28B).

**Scheme 29:**
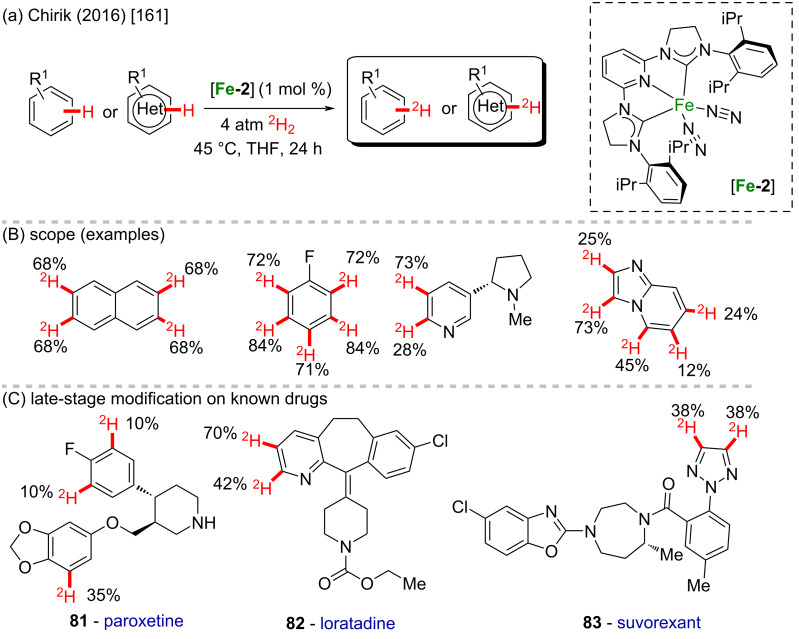
(A and B) Fe-catalyzed deuteration; (C) late-stage modification of pharmaceuticals.

In 2016, Chirik and co-workers described the deuteration of several pharmaceuticals via an Fe-catalyzed C–H activation protocol (Scheme 29A and B) [161]. The site selectivity of the bulky iron catalyst was orthogonal to conventional iridium catalysts used in deuterium labelling experiments, allowing the functionalization of complementary positions in several molecules of medicinal importance. Using molecular deuterium gas, the deuterium exchange occurred at different positions in small molecules in different proportions and satisfactory yields. Late-stage site-selective deuteration of pharmaceuticals like paroxetine (**81**), loratadine (**82**), and suvorexant (**83**) was achieved in moderate yields (Scheme 29C).

**Scheme 30:**
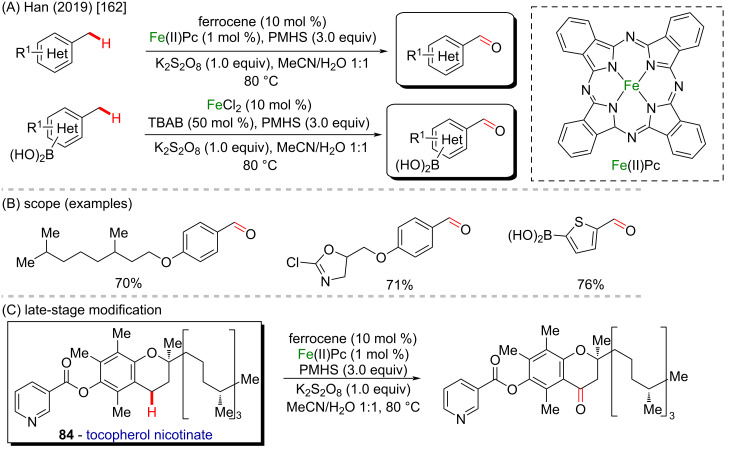
(A and B) Biomimetic Fe-catalyzed aerobic oxidation of methylarenes to benzaldehydes (PMHS, polymethylhydrosiloxane); (C) late-stage modification of tocopherol nicotinate (**84**).

In 2019, Han and collaborators reported a biomimetic Fe-catalyzed aerobic oxidation of methylarenes to benzaldehydes by using inexpensive and nontoxic reactants (Scheme 30A and B) [162]. The method was inspired by the biocatalytic action of the cytochrome P-450 cycle, which is driven by a reductase or bioreductant, and presented high versatility in incorporating both aldehyde and ketone functionalities into unprotected arylboronic acids. The reaction consists of using a porphyrin-based iron catalyst, and several scaffolds were successfully oxidized in good to excellent yields. The methodology also enabled the late-stage oxidation of complex molecules bearing benzylic C–H bonds like tocopherol nicotinate (**84**), which has never been demonstrated for any other catalytic oxidations of alkylaromatics (Scheme 30C).

**Scheme 31:**
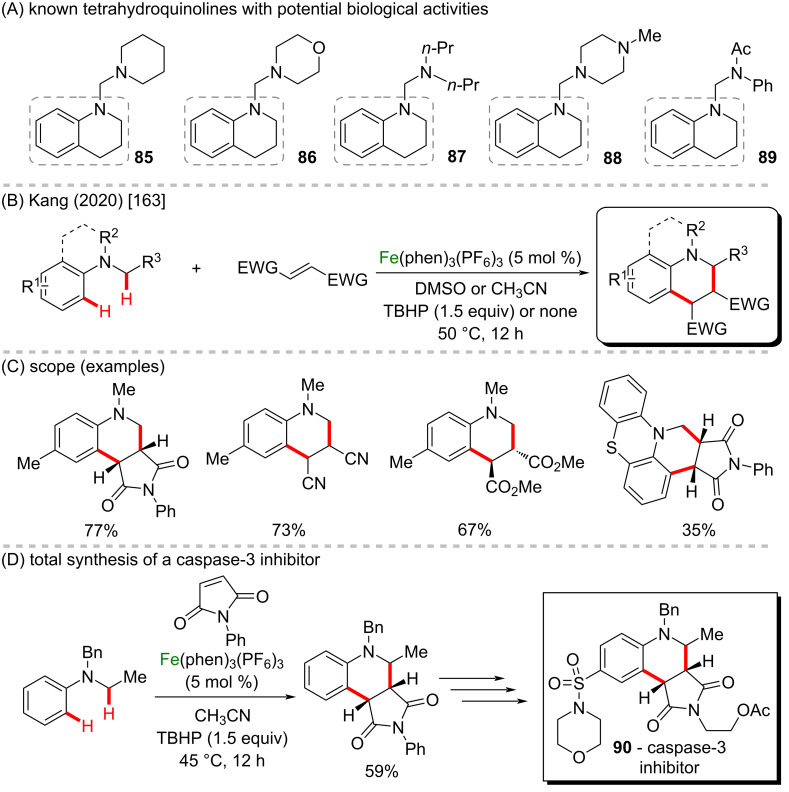
(A) Known tetrahydroquinolines with potential biological activities; (B and C) redox-selective Fe catalysis; (D) total synthesis of a caspase-3 inhibitor **90**.

A straightforward method for an α-amino C−H bond functionalization was described by Kang et al. in 2020 to promote the synthesis of tetrahydroquinolines from tertiary anilines (Scheme 31B and C) [163]. Tetrahydroquinolines are a class of compounds already known to present varied biological effects (**85**–**89**, Scheme 31A), such as antioxidant, α-amylase inhibitor, anticancer, and anti-inflammatory activities [164]. The reaction was promoted by non heme-Fe catalyst, behaving similarly to the bioinspired iron catalyst in a redox-selective way. The combination of Fe(phen)_3_(PF_6_)_3_ and tertiary anilines afforded α-aminoalkyl radicals that could be coupled with a wide range of electrophilic partners to afford the products in moderate to good yields. The new reaction was also used in the first step of the total synthesis of a caspase-3 inhibitor (**90**), and mechanistic investigations showed that O_2_ behaves as a terminal oxidant to form α-aminoalkyl radicals, whereas the formation of an Fe-peroxo species in the catalytic cycle was confirmed using a combination of EPR and ESI mass spectrometry experiments (Scheme 31D).

**Scheme 32:**
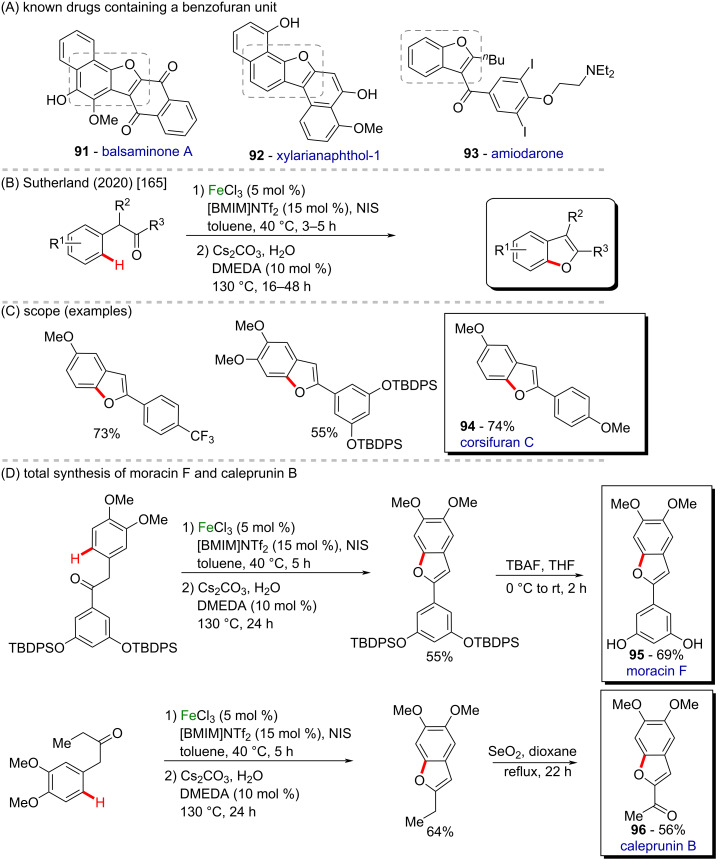
(A) Known drugs containing a benzofuran unit; (B and C) Fe/Cu-catalyzed tandem *O*-arylation to access benzo[*b*]furan derivatives; (D) total synthesis of moracin F (**95**) and caleprunin B (**96**).

One-pot processes for the synthesis of benzo[*b*]furans from aryl- or alkylketones using nonprecious Fe and Cu catalysis have been described by Sutherland and co-workers in 2020 (Scheme 32B and C) [165]. Benzofurans are important scaffolds present in several bioactive compounds, such as balsaminone A (**91**, antipruritic activity), xylarianaphthol-1 (**92**, anticancer activity), and amiodarone (**93**, antiarrhythmic activity) (Scheme 32A) [166]. The method consists of a tandem, regioselective Fe(III)-catalyzed C–H halogenation, followed by an Fe or Cu-catalyzed *O*-arylation to access the benzo[*b*]furan derivatives in high yields. Several natural products and pharmacologically active targets bearing the furan ring were synthesized in moderate to good yields. Overall, the new protocol presented a broad scope and excellent functional group tolerance, allowing the synthesis of valuable molecules like corsifuran C (**94**), moracin F (**95**), and caleprunin B (**96**) (Scheme 32D).

**Scheme 33:**
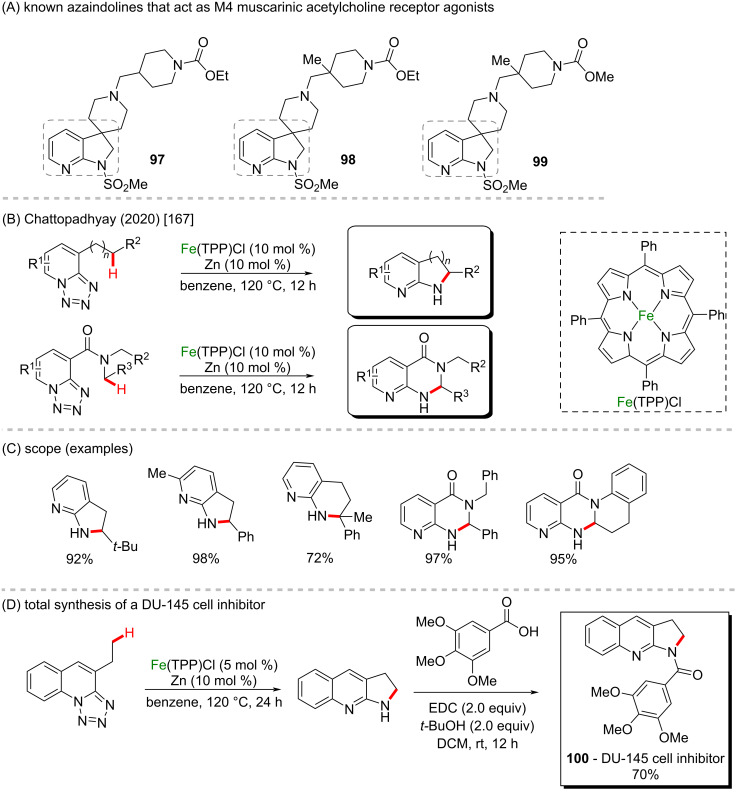
(A) Known azaindolines that act as M4 muscarinic acetylcholine receptor agonists; (B and C) intramolecular denitrogenative C(sp^3^)–H amination of 1,2,3,4-tetrazoles; (D) total synthesis of the DU-145 cell inhibitor **100**.

In 2020, Chattopadhyay and collaborators reported a new concept involving the intramolecular denitrogenative C(sp^3^)–H amination of 1,2,3,4-tetrazoles bearing unactivated primary, secondary, and tertiary C–H bonds via an Fe-catalyzed C–H activation (Scheme 33B and 33C) [167]. The new C(sp^3^)–H amination protocol presented high levels of selectivity, reactivity, and functional group tolerance, providing a large number of complex nitrogen heterocycles like azaindolines, pyrrolo-quinolines and -quinolones in excellent yields. Amongst the synthesized scaffolds, especially azaindolines are known to be present in M4 muscarinic acetylcholine receptor agonists (**97**, **98**, and **99**, Scheme 33A) [168]. Asymmetric variations of the porphyrin-based iron catalysts also provided the desired product with 100% conversion and enantiomeric ratios up to 73:27 in some cases. The straightforward Fe catalysis was used as a key step in the synthesis of a DU-145 cell inhibitor (**100**), that was obtained in 70% yield over two steps (Scheme 33D).

**Scheme 34:**
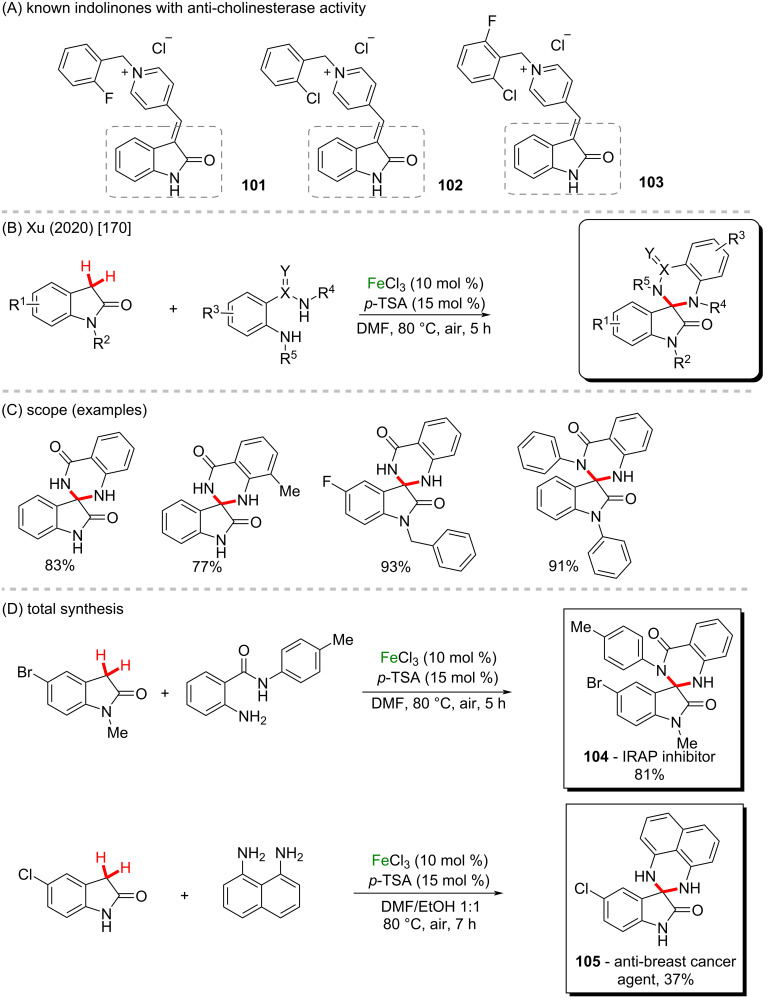
(A) Known indolinones with anticholinesterase activity; (B and C) oxidative C(sp^3^)–H cross coupling protocol; (D) total synthesis of representative bioactive compounds.

Indolones are known to present potent anticholinesterase activity (**101**, **102**, and **103**) (Scheme 34A). Therefore, the use of this class of molecules as substrates in organic functionalization methods is of high importance in the field of Alzheimer’s disease studies [169]. In 2020, Xu and co-workers described an unprecedented dual C–H functionalization of indolin-2-ones and benzofuran-2-ones via an oxidative C(sp^3^)–H cross-coupling protocol catalyzed by inexpensive FeCl_3_ and ligand-free conditions (Scheme 34B and 34C) [170]. The new method presented broad scope in the construction of tetrasubstituted carbon centers from methylenes to access a wide range of spiro *N*-heterocyclic oxindoles in excellent yields, including diamine, benzamide, and spirothiazole scaffolds. The high potential of the reaction was demonstrated by the synthesis of bioactive compounds like an IRAP inhibitor (**104**) and an antibreast cancer agent (**105**) in good yields (Scheme 34D).

Iron is by far the cheapest and most abundant metal to be used from the 3d series, and these facts are reflected by the amount and quality of studies that has been done so far. From the simplest iron salts to the most enantiospecific complex forms, iron catalysis is playing an important role for the development of additional accessible C–H activation methods. From these perspectives it can be assumed that in the near future iron will be one of the most studied metal applied in C–H functionalization methodologies.

### Cobalt-catalyzed C–H activation

**Scheme 35:**
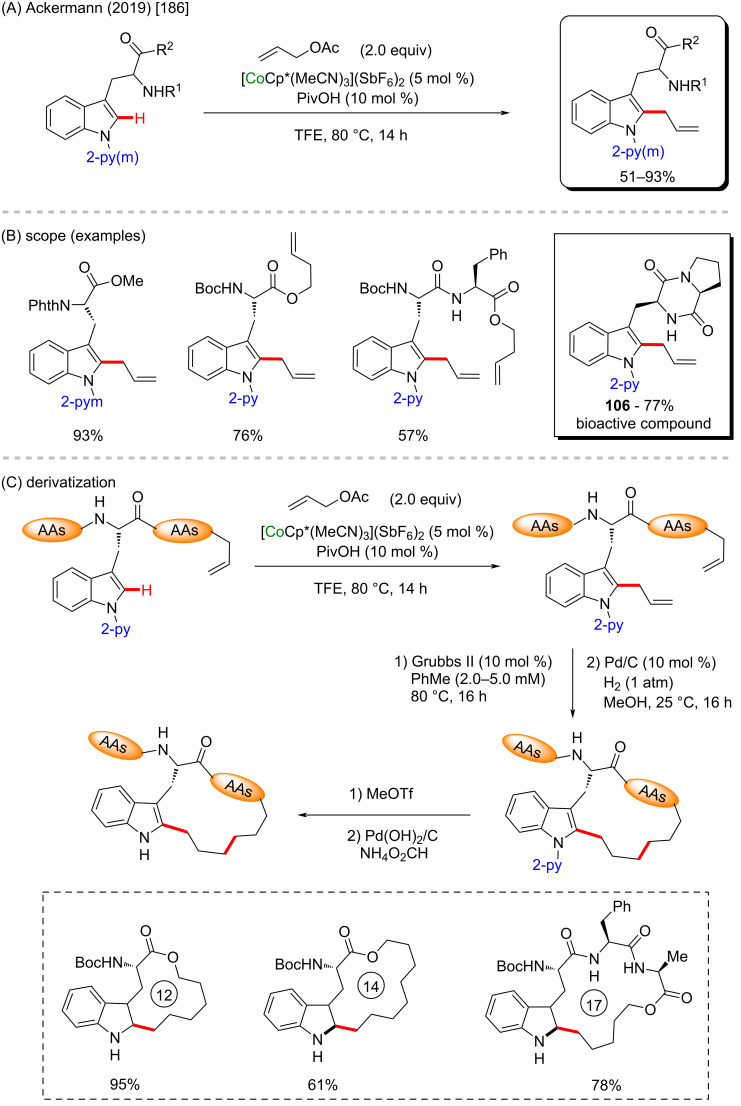
(A and B) Cobalt-catalyzed C–H alkenylation of C-3-peptide-containing indoles; (C) derivatization by Grubbs-II-catalyzed metathesis to macrocyclic peptides.

Cobalt is a cheap metal that presents powerful colors in its ionic forms and therefore, it has been used as a basic element in ink since ancient periods [171–172]. Cobalt alloys are used in blades [173] and batteries [174]. Due to its accessibility, it is well explored in catalysis for many fields, such as cycloaddition reactions [175], polymerization [176], and C–C cross-coupling methods [177]. Several biologically active compounds have been obtained through cobalt catalysis [178–180] including its application in C–H activation reactions [181–185]. The synthesis of biologically important compounds using C–H activation techniques has been described by Ackermann and co-workers in 2019 [186]. In this specific work, a cobalt-Cp* catalyzed C–H alkenylation was performed at the C-2 position of indole derivatives bearing peptide units in the C-3 position (Scheme 35A). This process led to several activated products in good yields, including one whose basic structure is already known to present important biological activities (**106**) [187] (Scheme 35B). Still in this work, several products were submitted to a subsequent metathesis mediated by Grubbs-II catalyst and a further palladium-catalyzed C=C reduction and DG-removal process, from which the desired macrocyclic peptides were obtained in good to excellent yields (Scheme 35C).

**Scheme 36:**
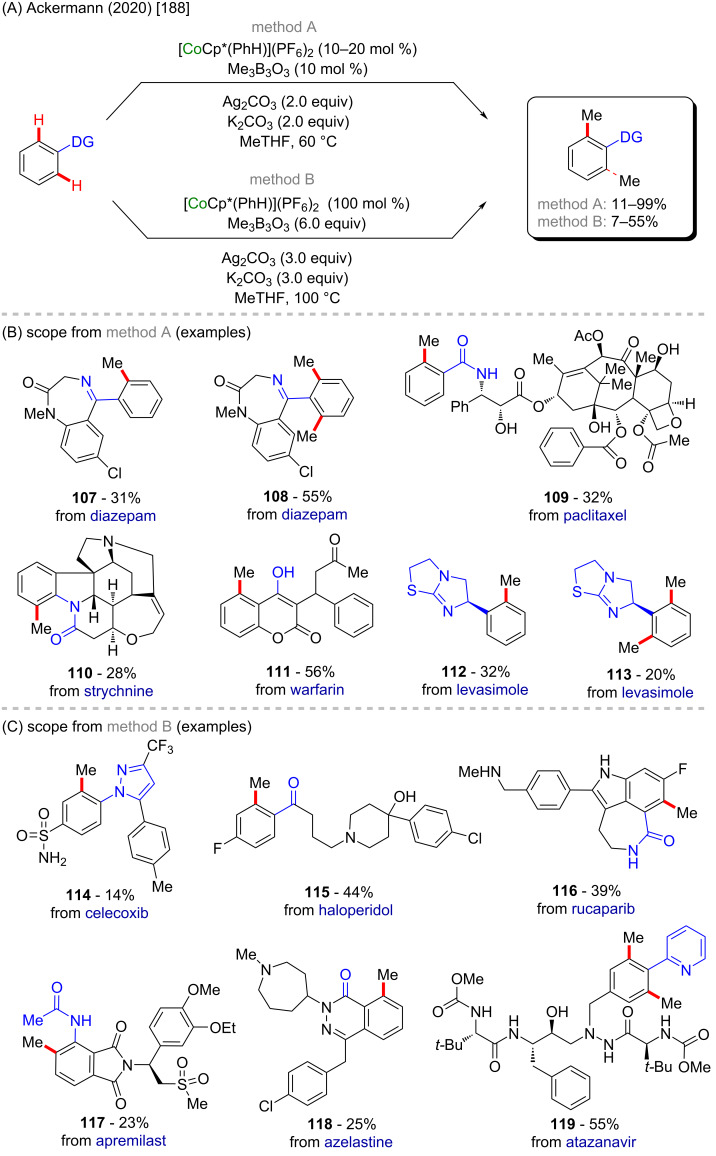
(A) Cobalt-Cp*-catalyzed C–H methylation of known drugs; (B and C) scope of the *o*-methylated derivatives.

Recently, again Ackermann and co-workers [188] described a cobalt-Cp*-catalyzed C–H methylation of several well-known pharmaceuticals, such as diazepam (a commercially available anxiolytic drug [189]), paclitaxel (a commercially available anticancer drug [190]), celecoxib (a commercially available analgesic drug [191]), and rucaparib (a commercially available anticancer drug [192]). They achieved the C–H methylation by following two methods (method A or method B, Scheme 36A) and obtained the methylated analogues **107**–**119** in moderate to very high yields (Scheme 36B and C).

**Scheme 37:**
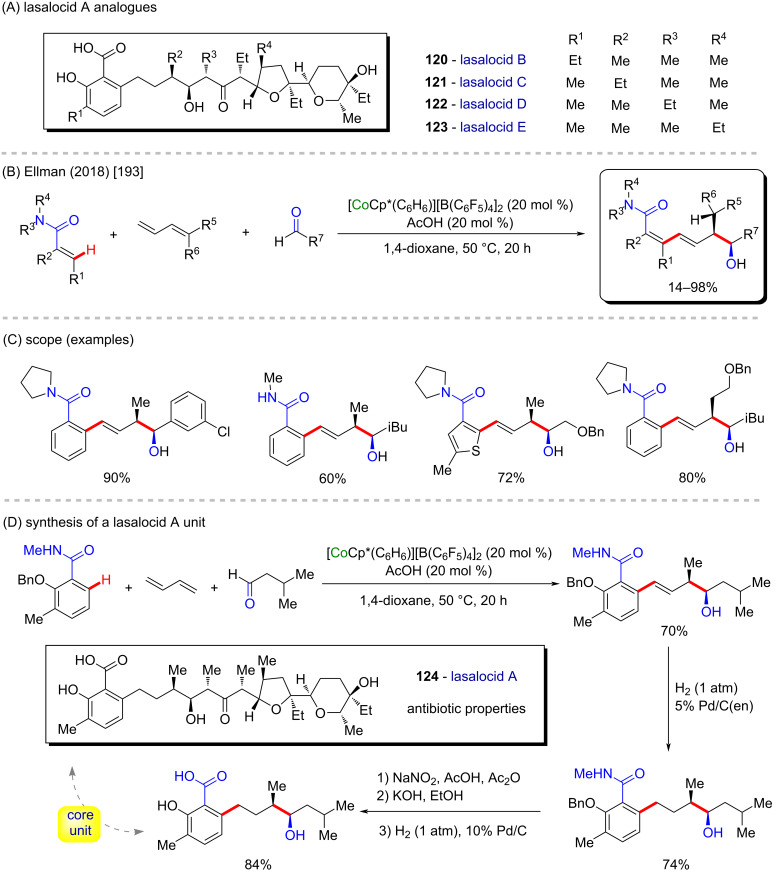
(A) Known lasalocid A analogues; (B and C) three-component cobalt-catalyzed C–H bond addition; (D) lasalocid A core unit synthesis.

Ellman and co-workers described a powerful and interesting three-component reaction in which a cobalt-Cp*-catalyzed C–H bond addition afforded complex scaffolds in good yields (Scheme 37B and C) [193]. The authors explained the stereoselectivity by means of a mechanism involving a hydride migration suffering influence of steric effects related not only to the catalyst ligands but also to the diene R^5^ and R^6^ substituents. With this innovative method in hands, the authors explored the synthetic applicability in the preparation of a core unit from lasalocid A (**124**), a known antibiotic drug [194] that, along with its four analogues **120**–**123** (Scheme 37A), were extracted from *Streptomyces lasaliensis* cultures [195]. The synthesis succeeded in five steps, each one of them in good yields, including the cobalt-catalyzed C–H bond addition (Scheme 37D).

**Scheme 38:**
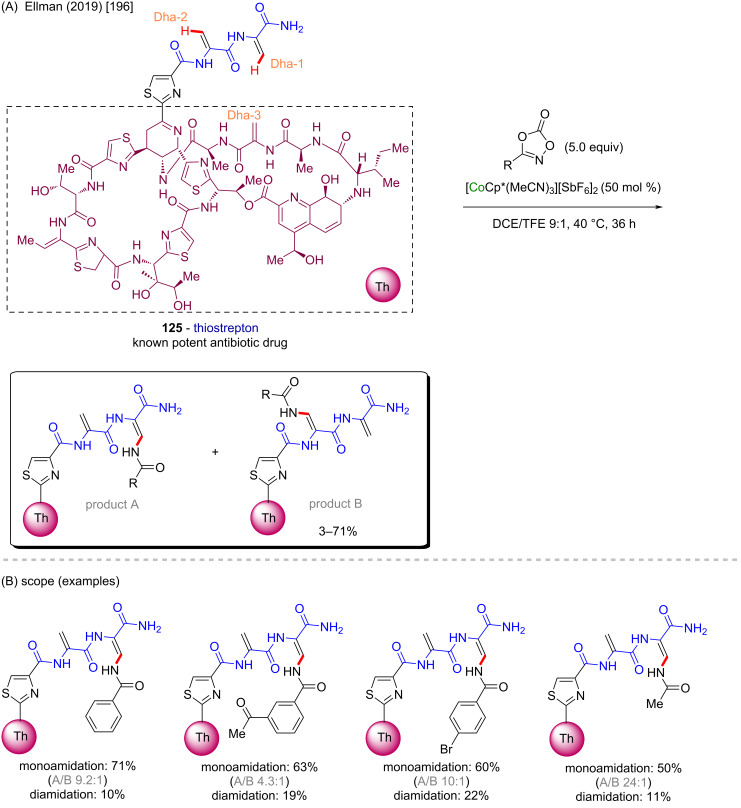
(A and B) Cobalt-catalyzed C(sp^2^)–H amidation of thiostrepton.

One year later, the same group described a unique cobalt-Cp* catalyzed C(sp^2^)–H amidation technique [196] applied to thiostrepton (**125**), a known biosynthetic antibiotic drug that presents a highly complex chemical structure [197] (Scheme 38A). The activation exclusively occurs at one of the hydrogen atoms of the dehydroalanine (Dha) portions Dha1 and Dha2, towards the formation of the *Z*-stereoisomer (Scheme 38A). No reaction was observed at the Dha-3 part and, according to the substitution pattern from the coupling partner, a higher preference for the substitution at the Dha-1 (product A) over the Dha-2 (product B) site was also observed (Scheme 38B), characterizing an excellent and valuable regio- and diastereoselectivity.

**Scheme 39:**
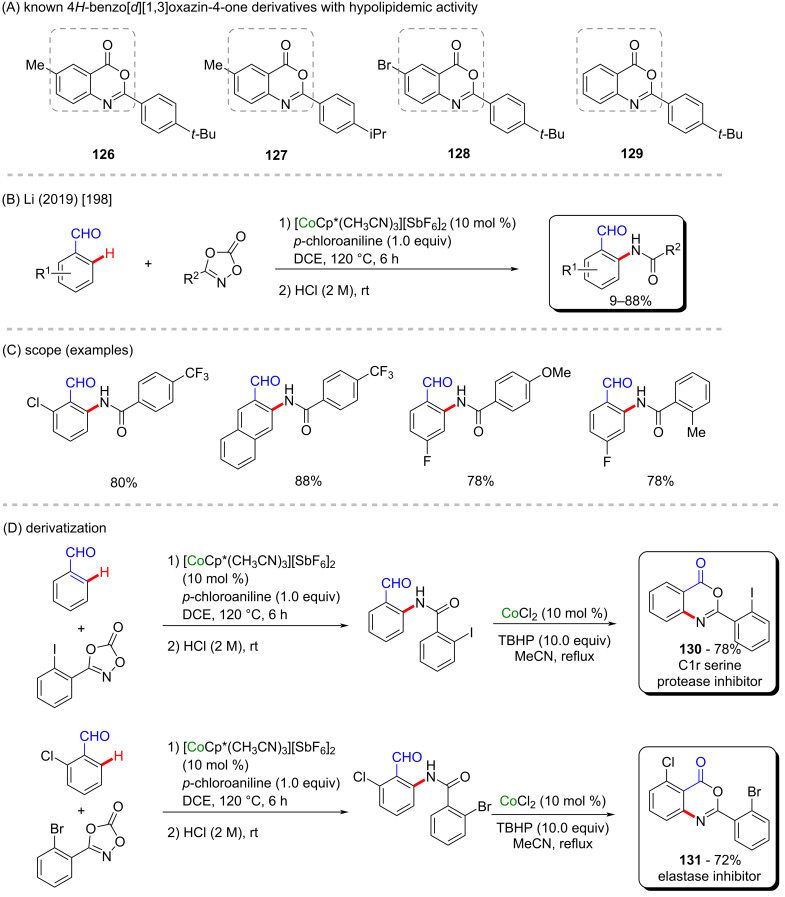
(A) Known 4*H*-benzo[*d*][1,3]oxazin-4-one derivatives with hypolipidemic activity; (B and C) cobalt-catalyzed *ortho*-directed C–H amidation of benzaldehyde derivatives; (D) application of the synthesis to important enzyme inhibitors.

In the same year, Wu, Li and co-workers described a successful cobalt-Cp*-catalyzed C–H amidation of benzaldehyde derivatives (Scheme 39B), in which the aldehyde portion works as the directing group [198]. After an acid workup using diluted hydrochloric acid, the desired *ortho*-amidated products were obtained in good yields (Scheme 39C). The authors used the same methodology to synthesize two 4*H*-benzo[*d*][1,3]oxazin-4-one derivatives that act as inhibitors of two enzymes (compounds **130** and **131** in Scheme 39D). The first one is the enzyme C1r serine protease, involved in both inflammation and renal scarring [199], and the second one is the enzyme elastase, responsible for consuming elastine, leading to aging processes [200]. Beyond that, 4*H*-benzo[*d*][1,3]oxazin-4-one derivatives **126**–**129** have been studied as potential hypolipidemic drugs (Scheme 39A) [201].

**Scheme 40:**
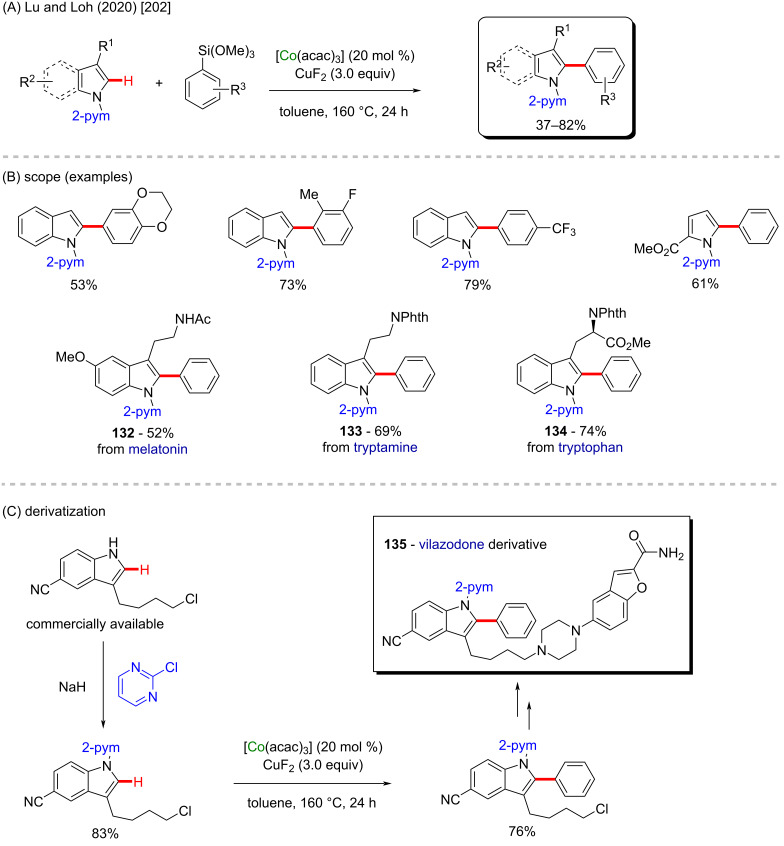
(A and B) Cobalt-catalyzed C–H arylation of pyrrole derivatives; (C) application for the synthesis of a vilazodone derivative.

Beyond the above-cited cobalt-Cp* examples, other classes of cobalt catalysts can also be used to affect C–H activation in different substrates. A good example is the use of chelated cobalt(III) catalysts, such as [Co(acac)_3_], as recently mentioned by Lu, Loh and co-workers [202]. In this work, the catalyst was used to mediate a C–H arylation at the C-2 position of *N*-(2-pyrimidyl)pyrrole derivatives (Scheme 40A). Several activated products were obtained in moderate to good yields (Scheme 40B). The authors extrapolated this methodology, using it to synthesize a derivative of vilazodone (**135**, Scheme 40C), a known antidepressant drug commercialized under the name viibryd which also bears potential activity against Parkinson’s disease [203].

**Scheme 41:**
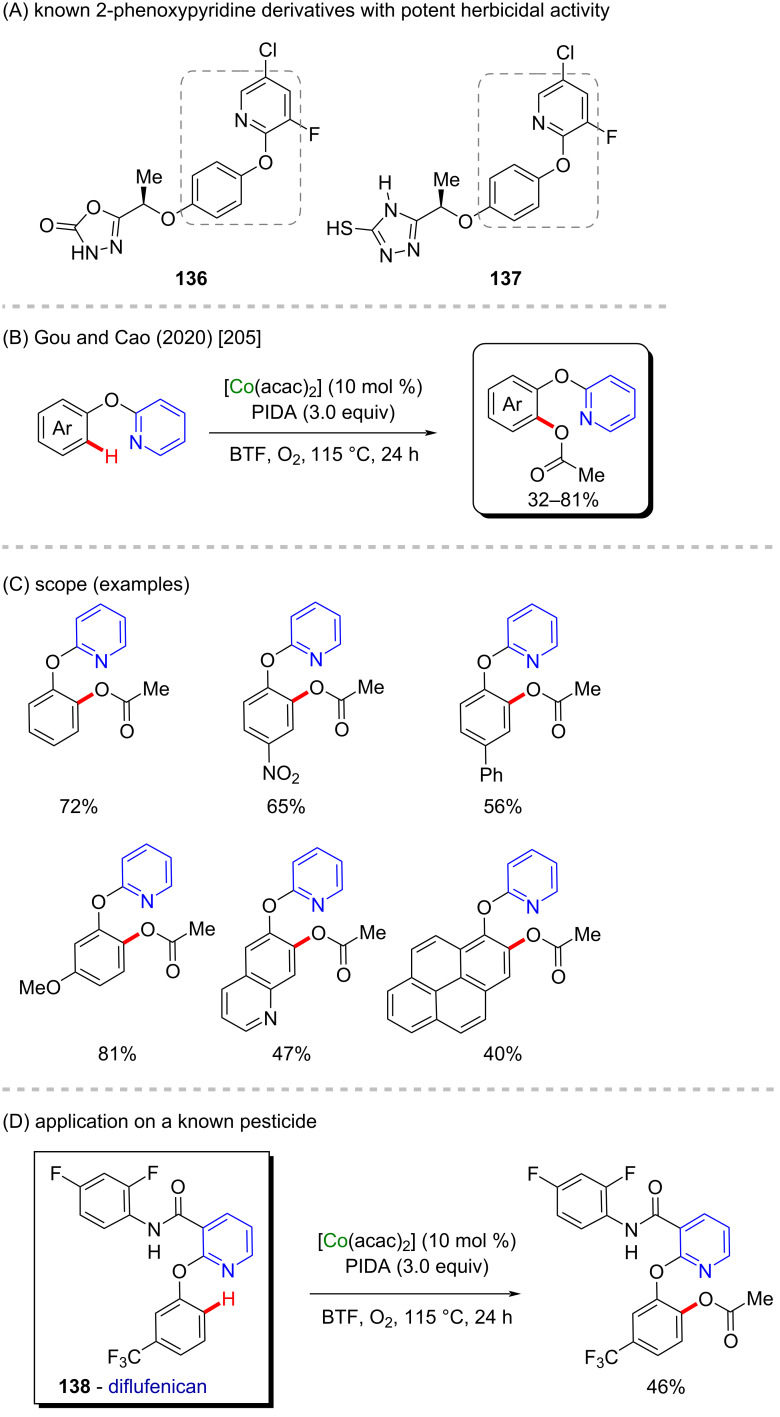
(A) Known 2-phenoxypyridine derivatives with potent herbicidal activity; (B and C) cobalt-catalyzed C–H acetoxylation of 2-(aryloxy)pyridine derivatives; (D) application to the functionalization of diflufenican (**138**).

In previous studies it has been observed that 2-phenoxypyridines belong to a class of compounds that presents potent herbicidal properties (**136**, **137**, Scheme 41A) [204]. Chelated cobalt(II) catalysts, such as [Co(acac)_2_], can also be used in C–H activation methods to modify 2-phenoxypyridines, from which other biological activities can be discovered. Although they present a lower oxidation state, they are also powerful catalysts, as described by Gou, Cao, and co-workers for a C–H acetoxylation of phenol derivatives (Scheme 43B) [205]. In the presence of phenyliodine(III) diacetate (PIDA) the reaction leads to several *ortho*-directed acetoxylated products in moderate to good yields (Scheme 41C). The authors used the same method in a late-stage functionalization of the *ortho*-position of diflufenican (**138**, Scheme 41D), a known commercial pesticide [206].

**Scheme 42:**
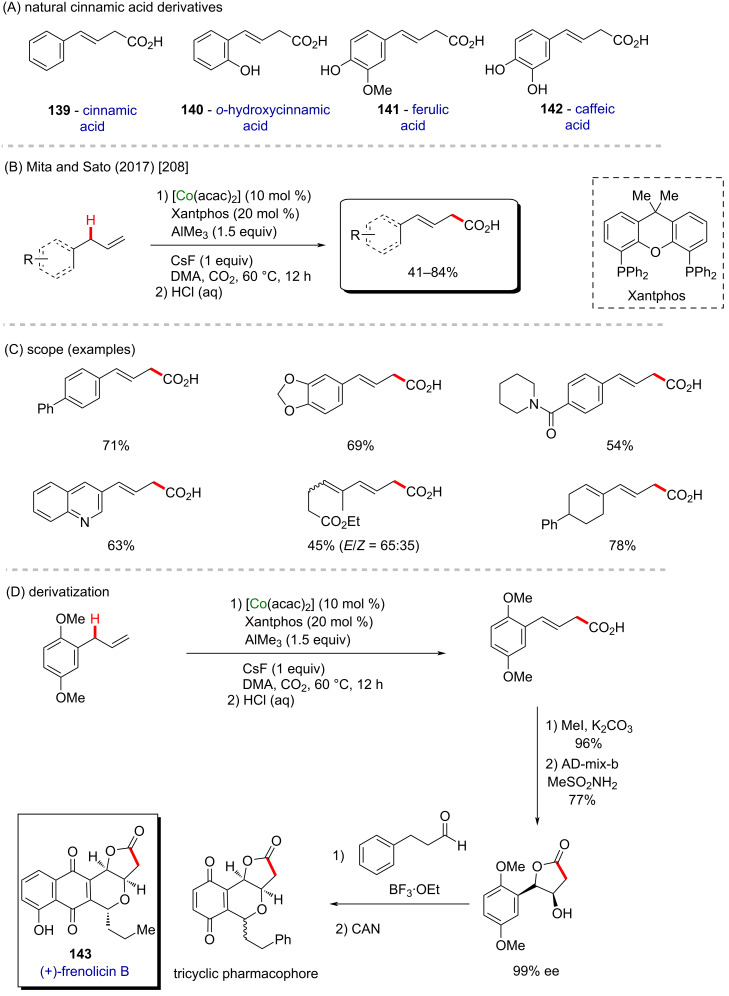
(A) Natural cinnamic acid derivatives; (B and C) cobalt-catalyzed C–H carboxylation of terminal alkenes; (D) application of the method to the synthesis of a (+)-frenolicin B derivative (**143**).

Cinnamic acid and its derivatives **139**–**142** are commonly found in food consumed daily, and are known for their antidiabetic properties (Scheme 42A) [207]. In 2017, Mita, Sato, and co-workers published a C(sp^3^)–H carboxylation using the same cobalt(acac)_2_ catalyst mentioned in the previous example for the synthesis of cinnamic acid derivatives and analogues [208]. The authors used CO_2_ as a carbonyl source in the presence of a Lewis acid (AlMe_3_), and a bulky ligand (Xantphos), followed by acid treatment (Scheme 42B), to promote the tautomerization that leads to the formation of the desired terminal carboxylic acids in good yields (Scheme 42C). The chemo- and regioselective method was applied, along with other cyclization/oxidation methods for the preparation of a tricyclic quinoidal compound, resembling the structure of (+)-frenolicin B (**143**, Scheme 42D), a known natural product extracted from the ferment supernatant residue from *Streptomyces sp.* that presents fungicidal activity [209].

**Scheme 43:**
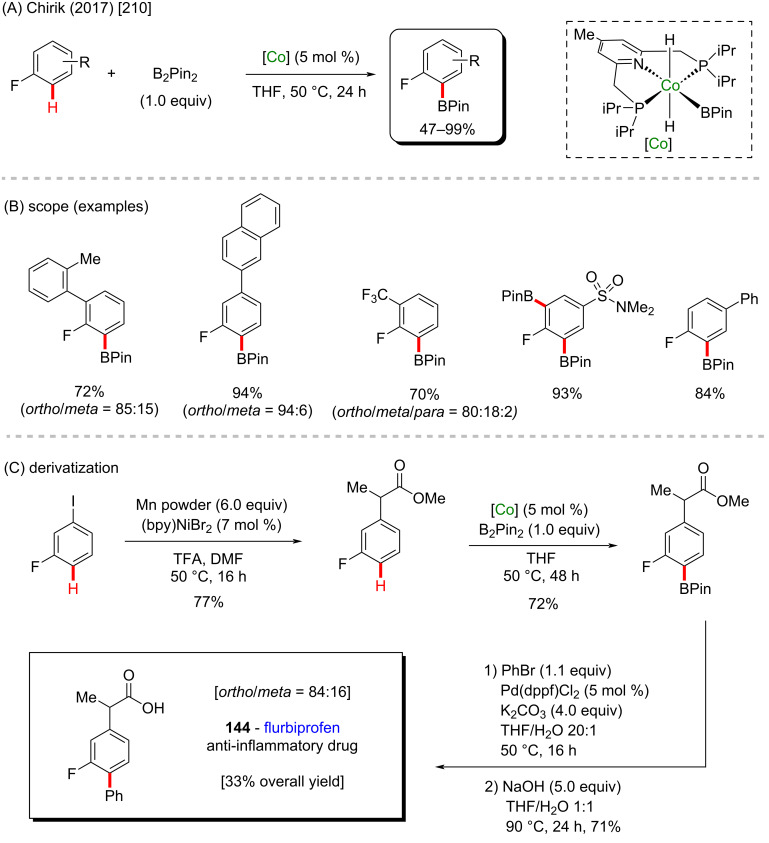
(A and B) Cobalt-catalyzed C–H borylation; (C) application to the synthesis of flurbiprofen.

A cobalt(III)-catalyzed C–H borylation was described by Chirik and co-workers in 2017 where they used a unique chelated cobalt catalyst (Scheme 43A) [210]. In this case, the electronic intrinsic effects of fluorinated arenes were responsible for the innate *ortho*-direction of this reaction, from which several borylated compounds were achieved in excellent yields. However, some of them were obtained as an *ortho/meta* mixture, with higher selectivity towards the *ortho*-isomers (Scheme 43B). Using the same method, the authors also described the successful synthesis of flurbiprofen (**144**), a potent anti-inflammatory drug [211], that was obtained in a four-step procedure, starting from the commercially available 3-iodofluorobenzene (Scheme 43C).

**Scheme 44:**
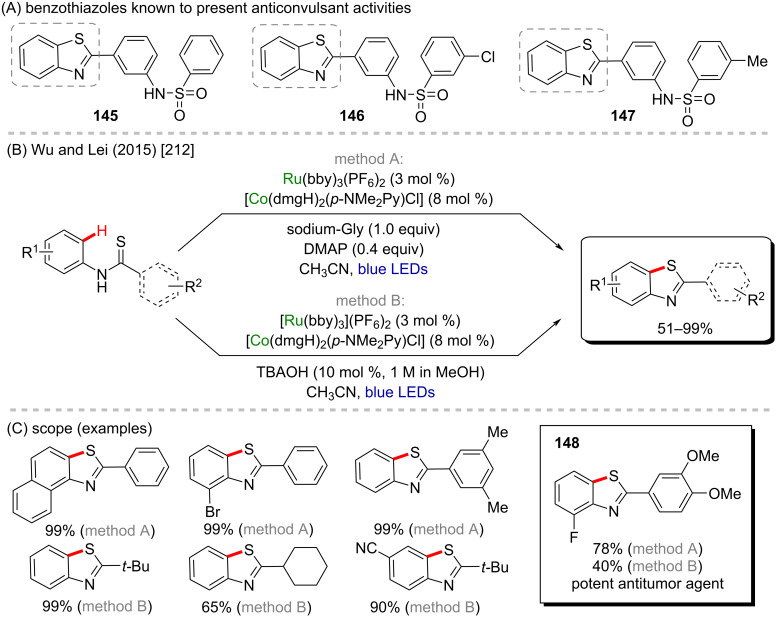
(A) Benzothiazoles known to present anticonvulsant activities; (B and C) cobalt/ruthenium-catalyzed cross-coupling reaction towards an intramolecular cyclization via the C–H thiolation of thioamides.

An interesting cobalt/ruthenium cross-coupling cyclization was described by Wu and Lei in 2015 [212]. In this work, the authors described an intramolecular photoredox cobalt-catalyzed C–H thiolation of thioamide substrates using two different methods (Scheme 44B). Using both methods, several benzothiazoles were obtained in excellent yields, most of them higher than 90% (Scheme 44C). Amongst the obtained products, one of them (**148**) is already known for its important antitumor activity [213]. Beyond that, benzothiazoles **145**–**147** present anticonvulsant activity (Scheme 44A) [214].

**Scheme 45:**
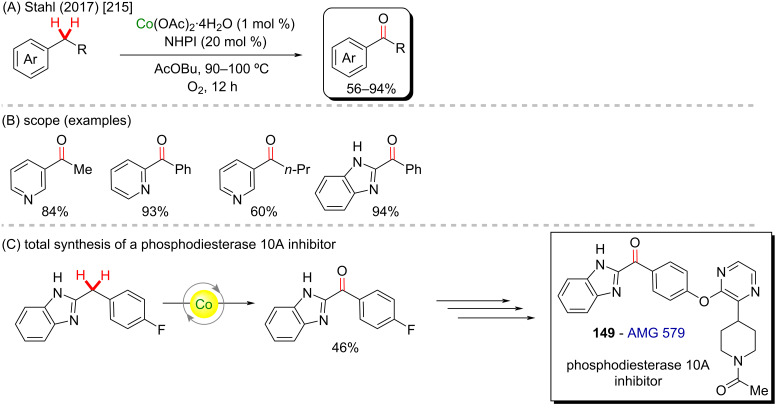
(A and B) Cobalt-catalyzed oxygenation of methylene groups towards ketone synthesis; (C) synthesis of the AMG 579 precursor **149**.

More simple cobalt(II) catalysts can also mediate valuable C–H activation processes, as exemplified by Stahl and co-workers in 2017 [215]. In this work, a cobalt-catalyzed aerobic oxidation leads to the formation of several ketones in good to excellent yields (Scheme 45A and B). One of the obtained products is a precursor of AMG 579 (**149**), an important phosphodiesterase 10A inhibitor (Scheme 45C) [216], being a potentially useful drug to treat schizophrenia.

**Scheme 46:**
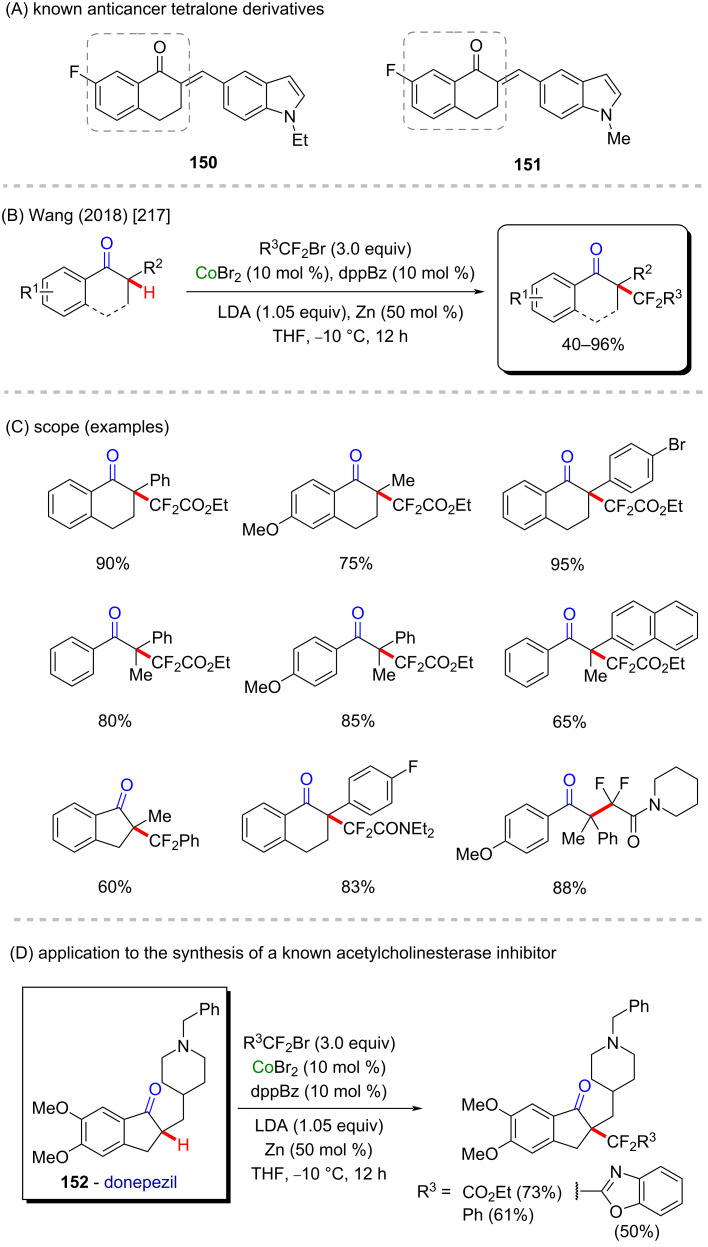
(A) Known anticancer tetralone derivatives; (B and C) cobalt-catalyzed C–H difluoroalkylation of aryl ketones; (D) application of the method using donepezil (**152**) as the substrate.

Wang and co-workers described a C–H difluoroalkylation method using only cobalt(II) bromide as catalyst (Scheme 46B) [217]. This fact represents a simple and highly accessible method for the synthesis of important compounds in good to excellent yields (Scheme 46C), mostly tetralones, an organic function present in anticancer substances (compounds **150** and **151**, Scheme 46A) [218]. The authors described a late-stage application of this new method using donepezil (**152**) as substrate (Scheme 46D), a well-known acetylcholinesterase inhibitor used to treat Alzheimer’s disease [219].

**Scheme 47:**
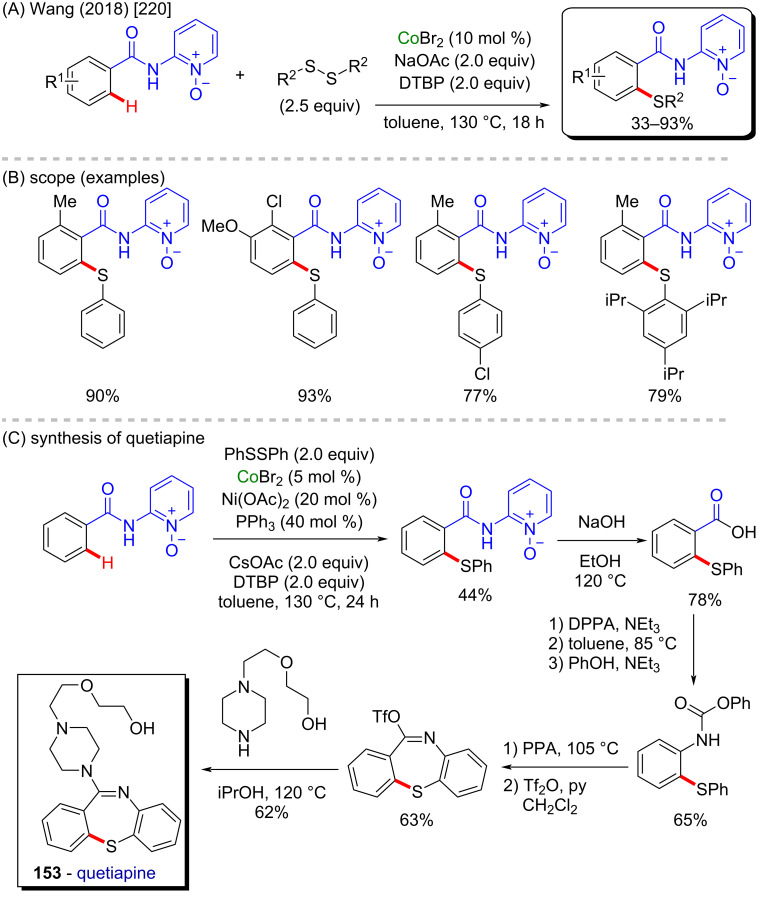
(A and B) Cobalt-catalyzed C–H thiolation; (C) application in the synthesis of quetiapine (**153**).

A successful *ortho*-directed cobalt-catalyzed C–H thiolation was described by Wang and co-workers in 2018 [220], also using solely cobalt(II) bromide as the cobalt source (Scheme 47A). Through this process, several *ortho*-activated products were obtained in good yields (Scheme 47B). The authors applied the process as one of the five steps towards the synthesis of quetiapine (**153**, Scheme 47C), a known antipsychotic agent [221].

**Scheme 48:**
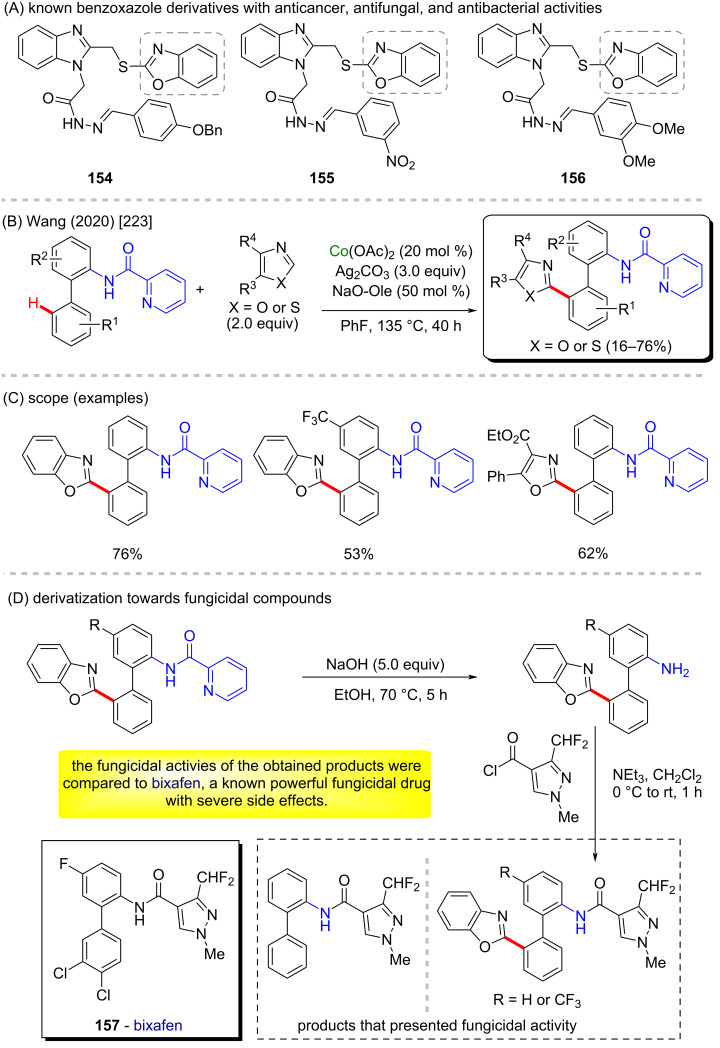
(A) Known benzoxazole derivatives with anticancer, antifungal, and antibacterial activities; (B and C) cobalt-catalyzed C–H/C–H cross-coupling; (D) synthesis of compounds with antifungal activities.

Benzoxazoles belong to a class of organic compounds known to present several biological activities, such as anticancer, antibacterial, and antifungal effects (compounds **154**, **155**, and **156**, Scheme 48A) [222]. Wang and co-workers described an important cobalt-catalyzed C–H/C–H cross-coupling reaction using cobalt(II) acetate and silver(I) carbonate (Scheme 48B) [223]. This method enabled the formation of 2‑(2-arylphenyl)azoles, mostly benzoxazoles, in moderate to good yields (Scheme 48C). The authors also modified the directing-group moiety of two products and one of the substrates and explored the fungicidal activity of the obtained final products (Scheme 48D) against four fungal species: *Fusarium graminearum*, *Physalospora piricola*, *Rhizactonia cerealis*, and *Bipolaris maydis*. A comparison of the observed activities to the activity of bixafen (**157**, a known fungicidal agent with severe side effects [224]) indicated that the new compounds presented valuable biological activity and may represent promising new antifungal drugs.

**Scheme 49:**
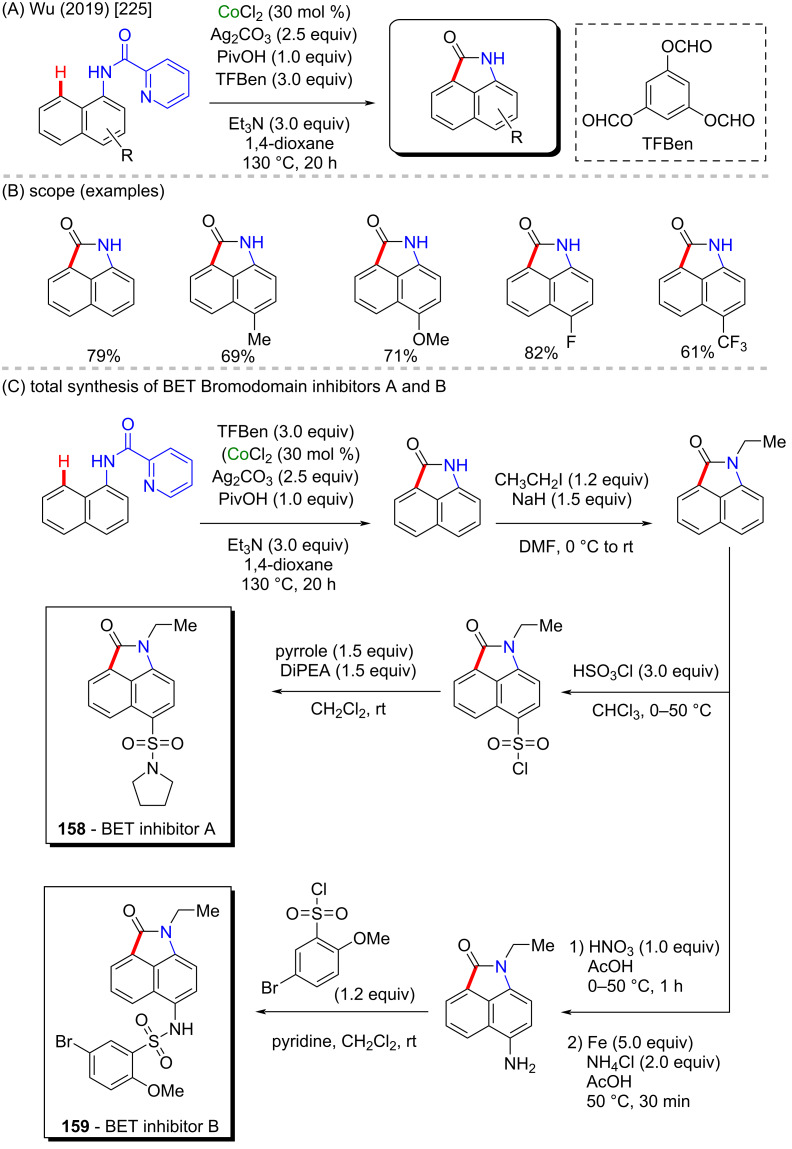
(A and B) Cobalt-catalyzed C–H carbonylation of naphthylamides; (C) BET inhibitors **158** and **159** total synthesis.

Wu and co-workers reported a cobalt-catalyzed C–H carbonylation of naphthylamides to afford a series of free N*H*-benzo[*cd*]indol-2-(1*H*)-ones in moderate to high yields (Scheme 49A and B). This carbonylative methodology uses the practical and helpful solid reagent benzene-1,3,5-triyl triformate (TBFen) as a CO source [225]. The further application of this efficient C–H functionalization protocol demonstrated by the authors consisted in the synthesis of biologically active compounds containing the benzo[*cd*]indol-2-(1*H*)-one core which were shown to act as inhibitors of the Bromodomain and Extra-Terminal (BET) family A and B (compounds **158** and **159**, Scheme 49C) [226]. The BET family of proteins plays an important role in gene transcription and epigenetics by binding acetylated lysines and has emerged as a driver of tumorigenesis in diverse human cancers. In this sense, the inhibition of BET proteins is an attractive target for cancer drug discovery [227–228].

**Scheme 50:**
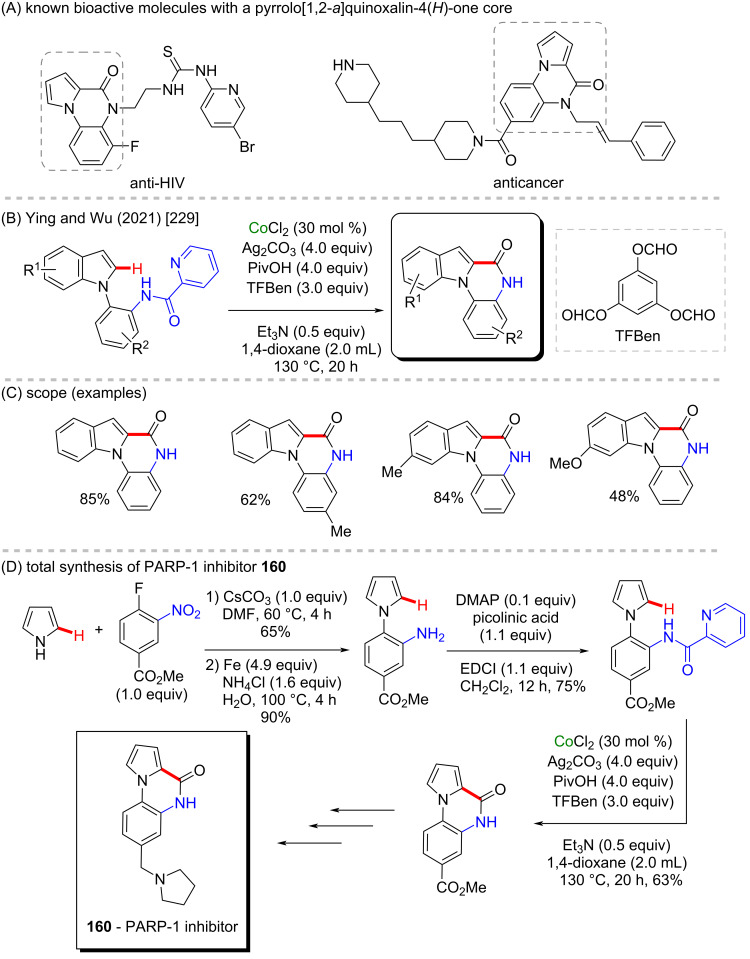
(A) Known bioactive pyrrolo[1,2-*a*]quinoxalin-4(5*H*)-one derivatives; (B and C) cobalt-catalyzed C–H carbonylation of *N*-arylindole derivatives; (D) PARP-1 inhibitor **160** total synthesis.

In a similar approach, Ying, Wu, and co-workers, reported the synthesis of the (*NH*)-indolo[1,2-*a*]quinoxaline-6(5*H*)-one core through a cobalt-catalyzed direct C–H carbonylative annulation of *N*-arylindoles using picolinamide as the directing group and employing TFBen as the CO source (Scheme 50B and C) [229]. This methodology was applied to a pyrrole derivative affording the pyrrolo[1,2-*a*]quinoxaline-(4*H*)-one skeleton, that after reductive amination afforded the poly(ADP-ribose)polymerase-1 (PARP-1) inhibitor **160** (Scheme 50D). PARP-1 is a nuclear enzyme that acts in some crucial cellular processes such as DNA repair and programmed cell death and is seen as a potential auxiliary in cancer treatment [230]. It is important to highlight here that this basic core is present in several known bioactive molecules [231] (Scheme 50A).

**Scheme 51:**
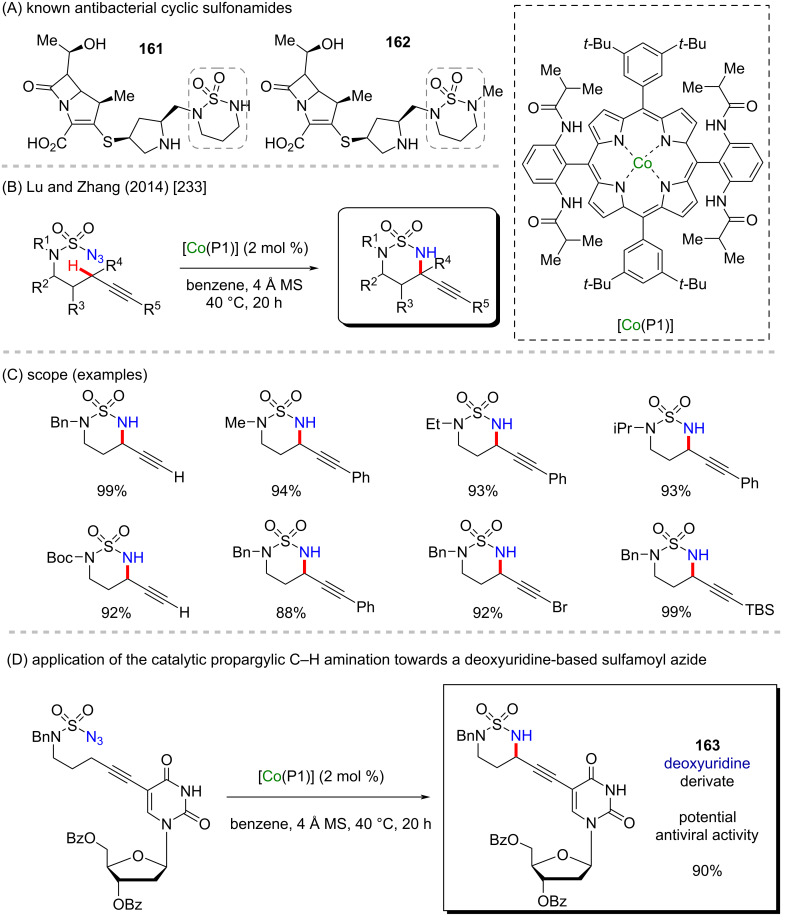
(A) Known antibacterial cyclic sulfonamides; (B and C) cobalt-catalyzed C–H amination of propargylic C–H bonds; (D) application of the method towards the synthesis of a deoxyuridine-based sulfamoyl azide.

Cyclic sulfonamides are encountered in compounds presenting potent antibacterial properties (compounds **161** and **162**, Scheme 51A) [232]. In 2014, Lu and Zhang reported a chemoselective intramolecular amination of propargylic C–H bonds of sulfamoyl azides using a cobalt–porphyrin complex ([Co(P1)]) as the catalyst (Scheme 51B). This cobalt–porphyrin complex exists as a stable metalloradical and was found to effectively activate azides as nitrene sources for the amination of the problematic propargylic C–H bonds [233]. Generally, the electrophilic nature of this bond makes the C≡C bonds preferred for amination over the propargylic bonds [234]. However, the reaction conditions allowed the practical access to numerous unsymmetric sulfamide derivatives (Scheme 51C). To prove the potential of the protocol in late-stage functionalizations, the authors synthesized the deoxyuridine derivative **163** (Scheme 51D). Deoxyuridine is similar to drugs like idoxuridine and trifluridine, which have been used as antiviral drugs [235].

**Scheme 52:**
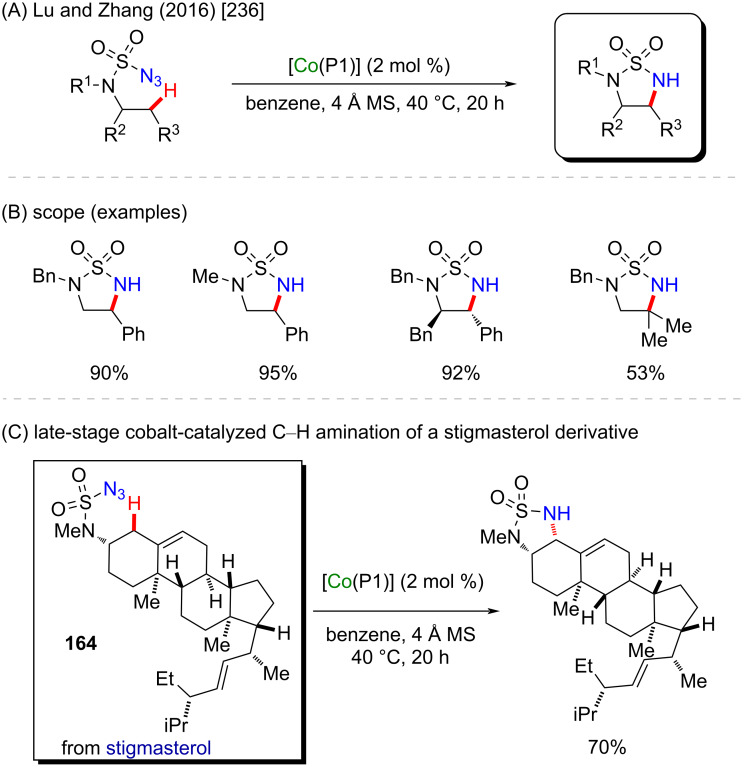
(A and B) Cobalt-catalyzed intramolecular 1,5-C(sp^3^)–H amination; (C) late-stage functionalization of stigmasterol derivative **164**.

In another interesting work, Lu and Zhang demonstrated the application of this protocol to construct new strained 5-membered cyclic sulfamides via a chemoselective intramolecular 1,5-C(sp^3^)–H amination. The cobalt(II)-based metalloradical (MRC) catalysis afforded the desired sulfamides in high yields and with nitrogen gas as the only byproduct (Scheme 52A and B) [236]. The application of this methodology in a late-stage functionalization of biologically active compounds was demonstrated by the [Co(P1)]-catalyzed 1,5-C–H amination of the allylic C–H bond of stigmasterol-based azide **164** in a chemo- and stereoselective fashion in 70% yield (Scheme 52C).

**Scheme 53:**
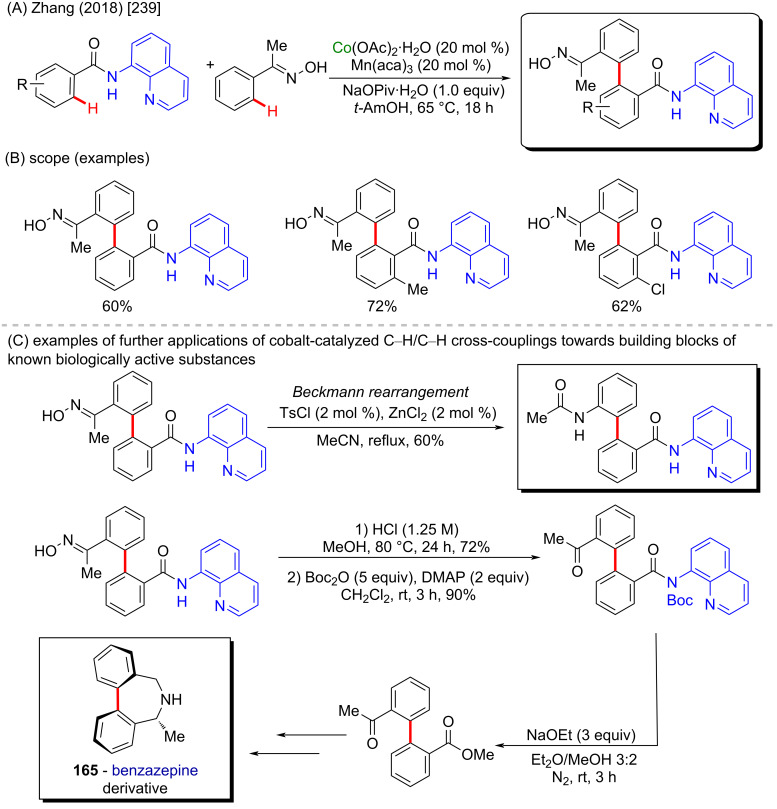
(A and B) Cobalt-catalyzed C–H/C–H cross-coupling between benzamides and oximes; (C) late-state synthesis of benzazepine derivative **165**.

Biaryl synthesis often is performed through well-established cross-coupling reactions. However, this type of reaction possesses some drawbacks like the necessity of prefunctionalized starting materials and the production of relatively expensive and stoichiometric amounts of toxic byproducts [237–238]. In 2018, Zhang and co-workers developed a method for biaryl synthesis involving the cobalt-assisted C–H/C–H cross-coupling between benzamides and oximes (Scheme 53A and B) [239]. Further transformations of the resulted biaryl compounds included a Beckmann rearrangement affording 2-amino-2′-carboxybiaryls, which are valuable synthetic precursors of some bioactive compounds [240]. Another example of a suitable post-modification of biaryl compounds was exemplified by the synthesis of benzazepine derivative **165**, as described by the authors (Scheme 53C). Benzazepines are present in some important pharmaceutical compounds, such as the antipsychotic drug clozapine [241–242].

**Scheme 54:**
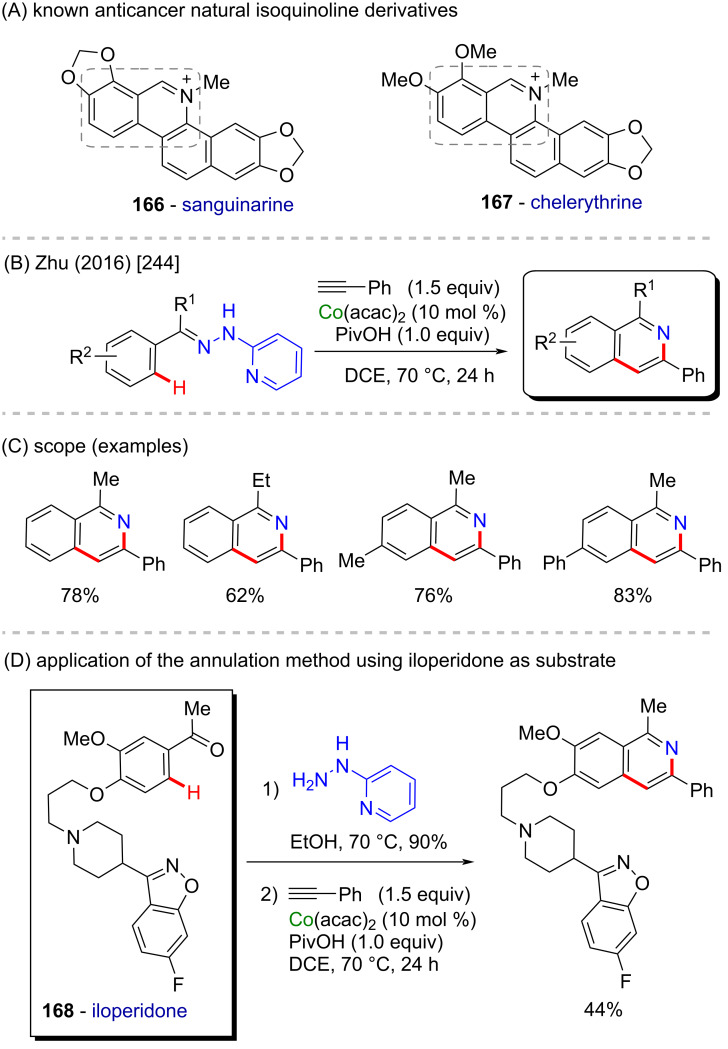
(A) Known anticancer natural isoquinoline derivatives; (B and C) cobalt-catalyzed C(sp^2^)–H annulation on 2-hydrazinylpyridine derivatives; (D) late-state annulation of iloperidone.

Several isoquinoline derivatives have been studied for their anticancer activity (compounds **166** and **167** in Scheme 54A) [243]. Zhu and co-workers reported a cobalt-catalyzed C(sp^2^)–H 2-hydrazinylpyridine-directed C–H annulation with alkynes providing new isoquinoline derivatives in 2016. The robust reaction performed in open air provides a broad scope of new isoquinolines (Scheme 54B and C) [244]. To demonstrate the applicability of this method, the authors applied of the protocol to iloperidone (**168**), a compound with known antipsychotic effects [245], as substrate for the cobalt-catalyzed annulation (Scheme 54D). The 2-hydrazinylpyridine auxiliary group was introduced in the ketone portion of iloperidone and the sequential C–H annulation afforded the corresponding isoquinoline derivative.

As demonstrated by the examples above, cobalt is another example of a well-established and well-explored metal applicable as a catalyst for C–H activation reactions. Cobalt-catalysis, allows to obtain a huge variety of compounds, including bioactive species, using both complex and simple catalysts. Therefore, this metal represents high potential for future developments in catalysis.

### Nickel-catalyzed C–H activation

**Scheme 55:**
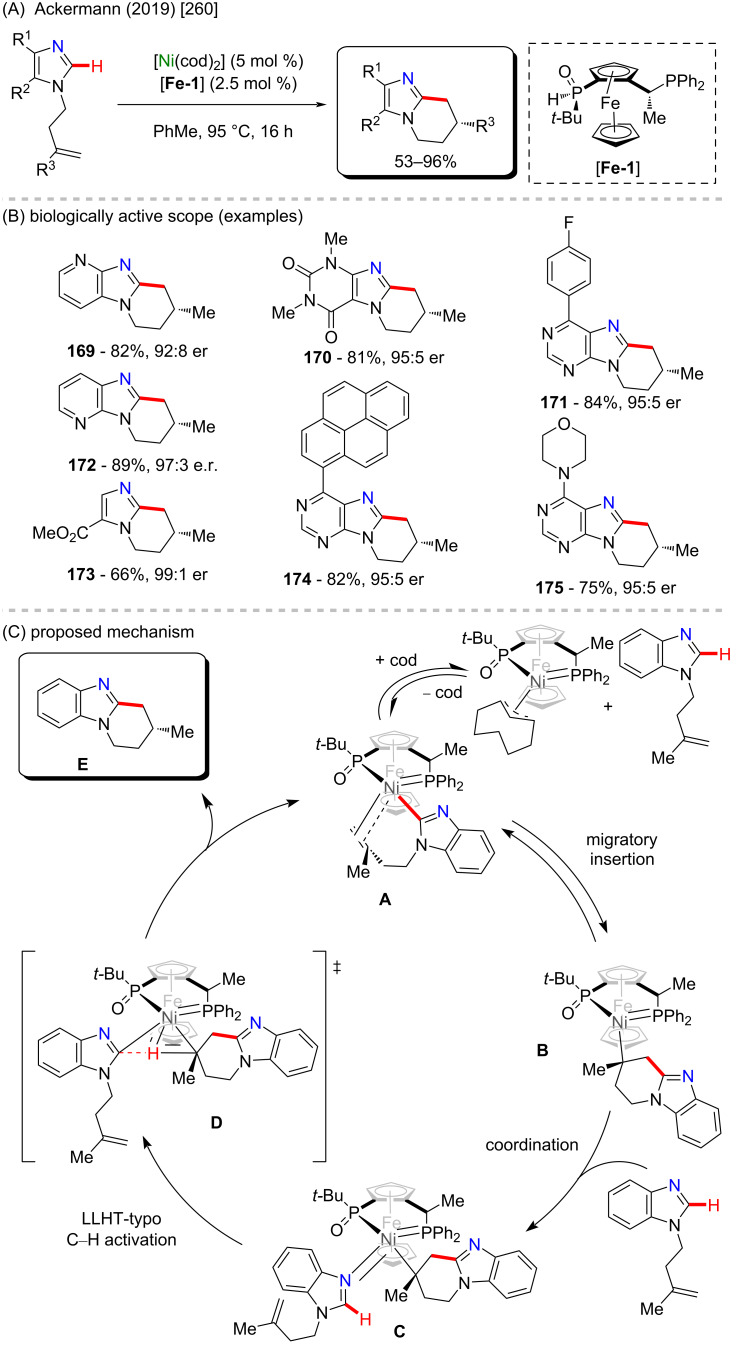
(A) Enantioselective intramolecular nickel-catalyzed C–H activation; (B) bioactive obtained motifs; (C) proposed mechanism.

Nickel is a versatile metal that is used in several products, from simple day-by-day coinage [246] to electrodes used for *Escherichia coli* detection in water [247]. It is one of the metals contained in stainless steel [248], a stable alloy used to construct several industrial products. It is also applied as a powerful catalyst in polymerization [249–250], C–C coupling [251], oxygen [252] or hydrogen evolution [253], or even cycloaddition reactions [254]. Its accessibility and high catalytic activity have been well-explored also in C–H activation processes [255–259]. Some of the discoveries led to the formation of important biologically active substances that play a crucial role in pharmaceutical studies, as exemplified by a work published by Ackermann and co-workers in 2019 [260]. In this work, the authors described an enantioselective intramolecular C–H activation of several imidazole derivatives in the presence of an effective chiral ferrocene ligand and nickel-cod as catalyst (Scheme 55A). Through this process, several 6-membered cyclic products were obtained in moderate to excellent yields and excellent enantiomeric ratios (er). Several known bioactive motifs were synthesized, such as theophylline and purine derivatives **169**–**175** (Scheme 55B) [261–263]. The authors also proposed a plausible mechanism (Scheme 55C). The chiral interaction between the ferrocene ligand, the substrate, and the nickel catalyst, form intermediate **A** that, after a migratory insertion, leads to the formation of the intermediate **B**, which already presents the desired chiral product coordinated to the nickel center. A subsequent coordination to another equivalent of the substrate leads to the formation of intermediate C. Through a C–H activation step, hydrogen is transferred from the second coordinated substrate moiety to the first coordinated substrate already present in the coordination sphere of the catalyst, which is represented by transition state **D**, that leads to the formation of the product **E** and the starting intermediate **A**.

**Scheme 56:**
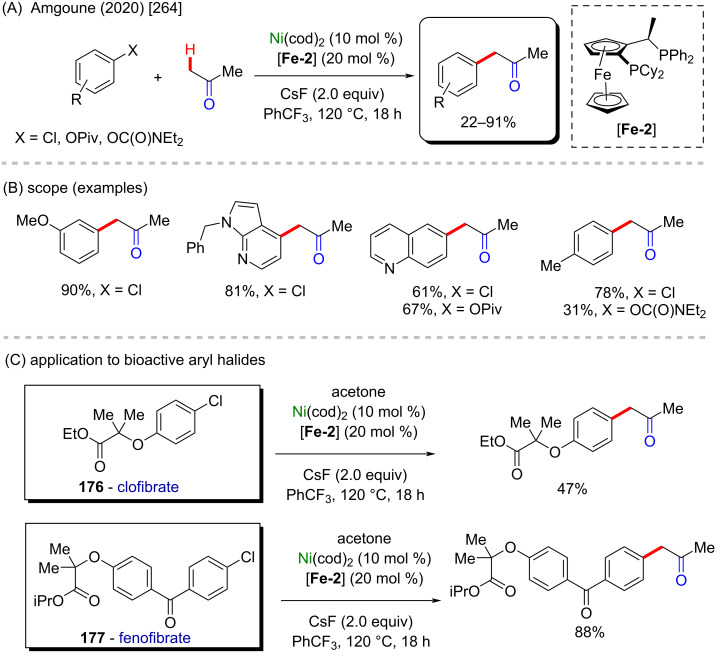
(A and B) Nickel-catalyzed α-C(sp^3^)–H arylation of ketones; (C) application of the method using known drugs as coupling partners.

The same catalyst used in the previous example (nickel-cod) was also explored very recently by Amgoune and co-workers in an α-C(sp^3^)–H arylation of ketones in the presence of another chiral ferrocene ligand [264]. The desired arylated products were successfully obtained in moderate to good yields (Scheme 56A and B). Amongst the obtained products, two were derived from the commercially available drugs clofibrate (**176**) [265] (trade name Atromid-S^®^) and fenofibrate (**177**) [266] (marketed under the name TriCor^®^), used to treat abnormal blood lipid levels (Scheme 56C).

**Scheme 57:**
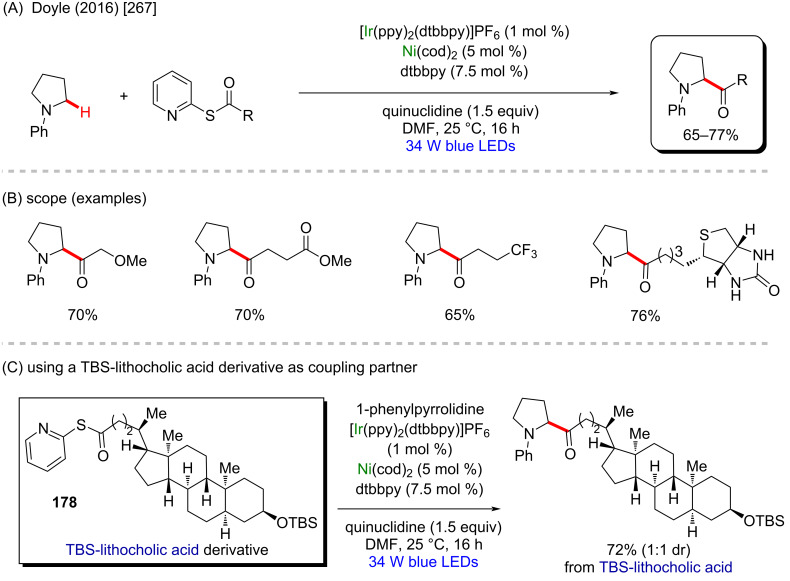
(A and B) Nickel-catalyzed C(sp^3^)–H acylation of pyrrolidine derivatives; (C) exploring the use of a steroidal residue as coupling partner.

In 2016, Doyle and co-workers described a notable nickel/photoredox-catalyzed C(sp^3^)–H acylation of several pyrrolidine derivatives [267]. They used thioesters as coupling-partners in the presence of [Ni(cod)_2_] and [Ir(ppy)_2_(dtbbpy)]PF_6_ (Scheme 57A and B). Valuable acylated products were obtained, including a complex one originating from TBS-lithocholic acid **178**, with a steroidal backbone (Scheme 57C).

**Scheme 58:**
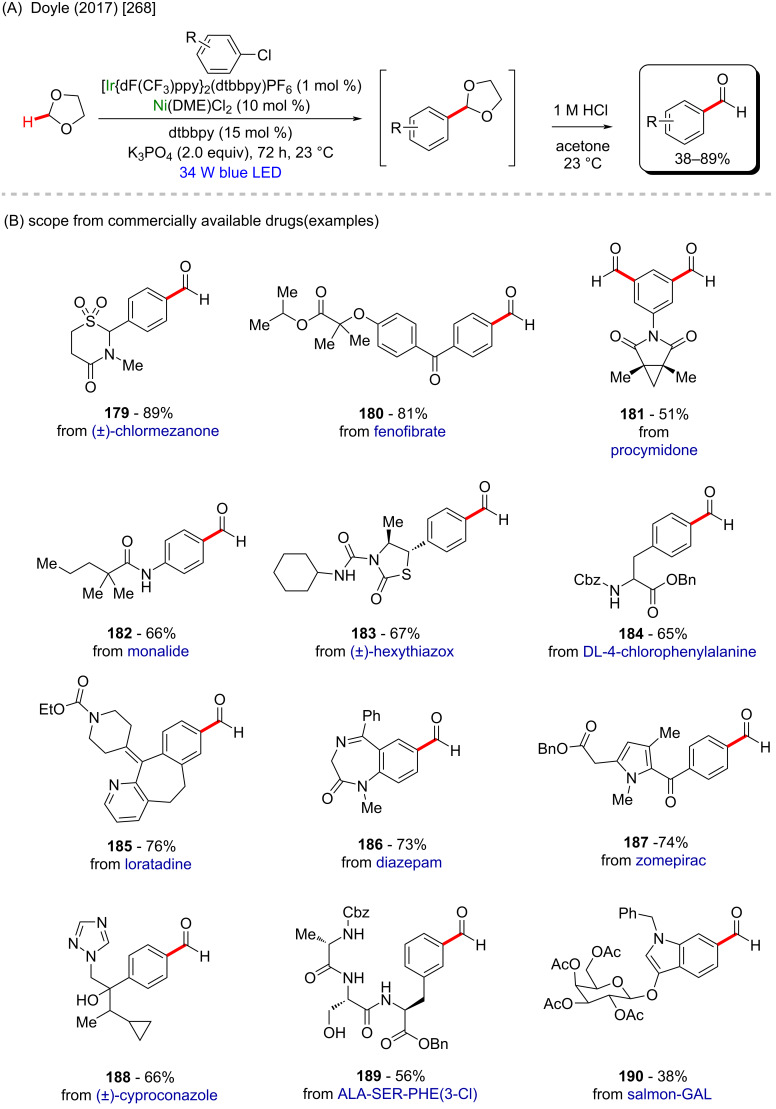
(A) Nickel-catalyzed C(sp^3^)–H arylation of dioxolane; (B) library of products obtained from biologically active aryl chlorides as coupling partners.

One year later, the same group published another notable work describing a nickel/photo-catalyzed C(sp^3^)–H arylation of dioxolane in the presence of several aryl chlorides (Scheme 58A) [268]. In this study, the authors built a library using known commercially available drugs as coupling partners, such as procymidone (**181**) (a known potent fungicide used in agriculture [269]), hexythiazox (**183**) (a known pesticide used in agriculture [270]) and zomepirac (**187**) (withdrawn anti-inflammatory drug [271]), from which the arylated products were obtained in moderate to good yields (Scheme 58B).

**Scheme 59:**
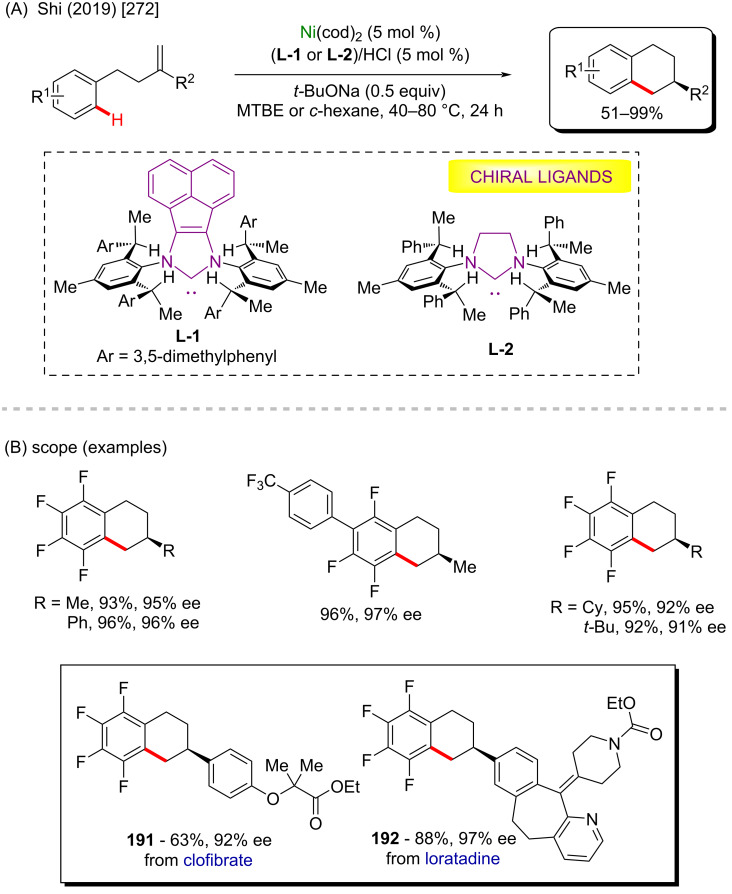
(A) Intramolecular enantioselective nickel-catalyzed C–H cycloalkylation; (B) product examples, including two drug derivatives.

Beyond the examples cited above, another interesting nickel-cod use was described by Shi and co-workers in 2019 [272]. In this work, an enantioselective intramolecular C–H cycloalkylation proceeded in the presence of robust chiral ligands (Scheme 59A). All cyclic products were obtained in good yields (Scheme 59B), and amongst the products, two derived from the known pharmacological substances, **191** from clofibrate (previously mentioned drug used to clean lipid levels in blood [265]) and **192** from loratadine (known antihistamine drug [273]).

**Scheme 60:**
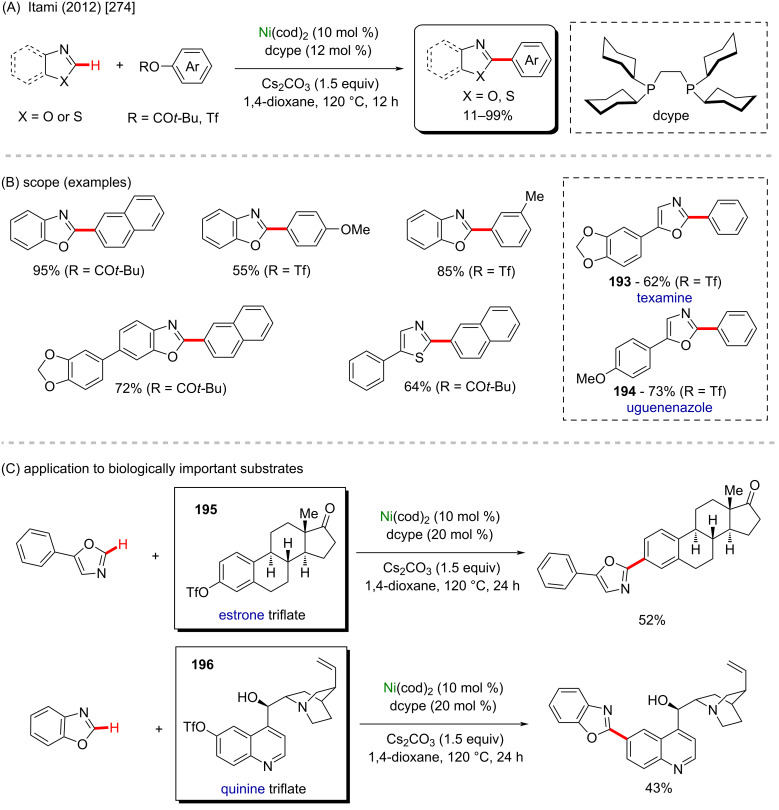
(A and B) Nickel-catalyzed C–H deoxy-arylation of azole derivatives; (C) late-stage functionalization on naturally occurring alkaloids.

In 2012, another interesting nickel-catalyzed C–H activation method was described by Itami and co-workers (Scheme 60A) [274]. In this work, a deoxy-arylation process of azoles led to the formation of several arylated products in moderate to good yields, including the important alkaloids texamine (**193**) [275] and uguenenazole (**194**) [276] (Scheme 60B). The authors applied the same developed methodology in a late-stage study to modify the derivative of the naturally occurring compounds estrone triflate (**195**) [277] and quinine triflate (**196**) [278] (Scheme 60C).

**Scheme 61:**
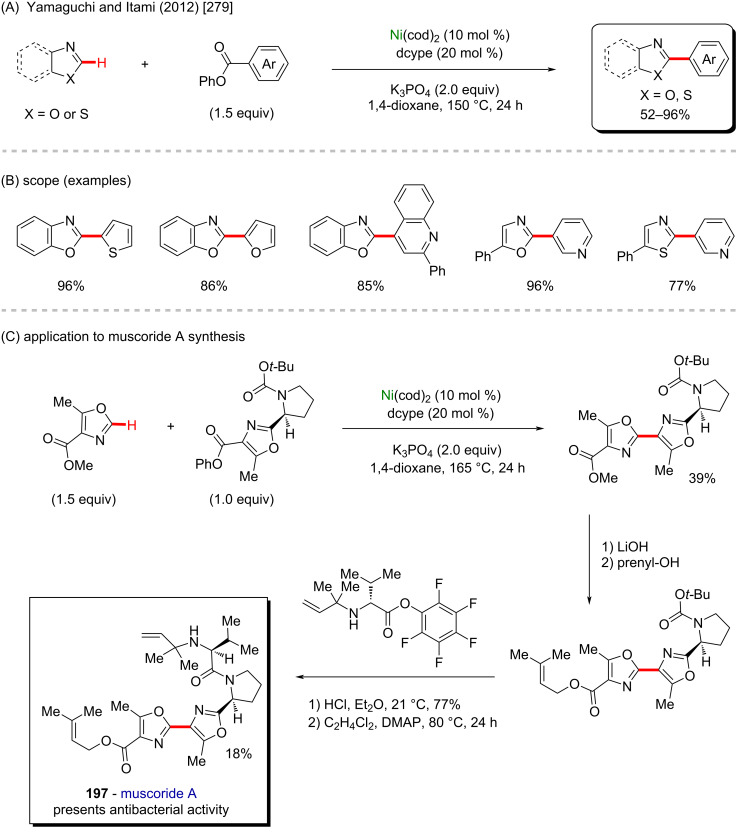
(A and B) Nickel-catalyzed decarbonylative C–H arylation of azole derivatives; (C) application of this method to the synthesis of muscoride A (**197**).

Yamaguchi, Itami and co-workers explored a similar procedure, in another report the same year [279], in which they described a nickel-cod-catalyzed decarbonylative C–H arylation of azole derivatives (Scheme 61A). This method afforded the desired products in good to excellent yields (Scheme 61B) and it was further applied to the total synthesis of muscoride A (**197**, Scheme 61C), a compound known to present antibacterial activities [280].

**Scheme 62:**
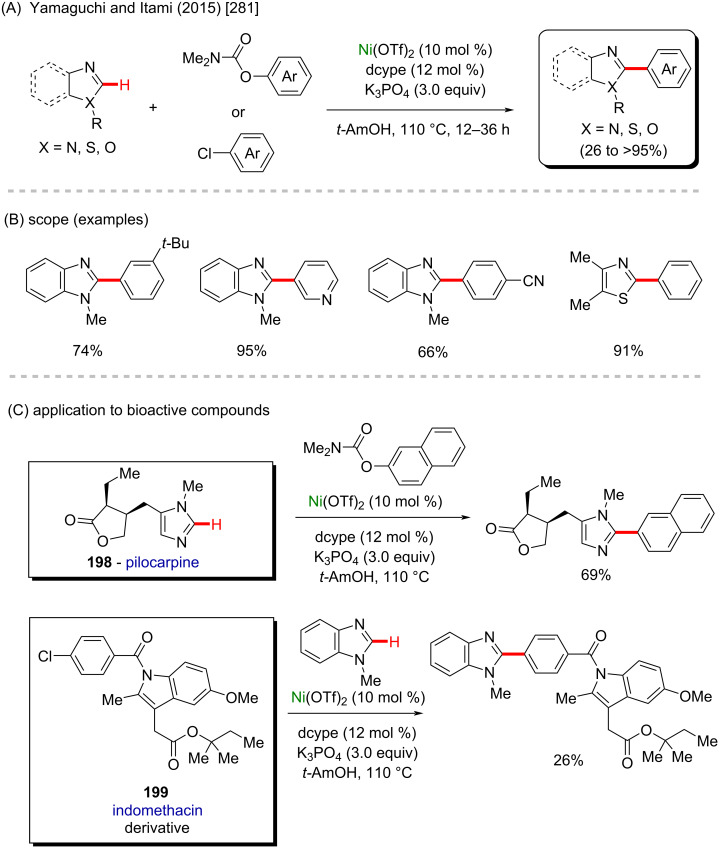
(A and B) Another important example of nickel-catalyzed C–H arylation of azole derivatives; (C) application of the method to bioactive compounds.

Further investigations from these authors of this innovative method led to the observation of a good catalytic activity of another nickel(II) source, as described in another work published in 2015 [281]. This work describes a nickel(II) triflate-catalyzed C–H arylation of azoles, by which several arylated products were successfully obtained in good yields (Scheme 62A and B). Amongst the obtained products, two are worth mentioning here since they derive from two important biologically active substances (Scheme 62C). The first one was obtained in 69% yield derived from pilocarpine (**198**), a known drug used in the treatment of glaucoma [282]. The second one derives from indomethacin (**199**), a known anti-inflammatory drug that, in combination with vitamin D, substantially decreases the frequency of colorectal cancer occurrence [283].

**Scheme 63:**
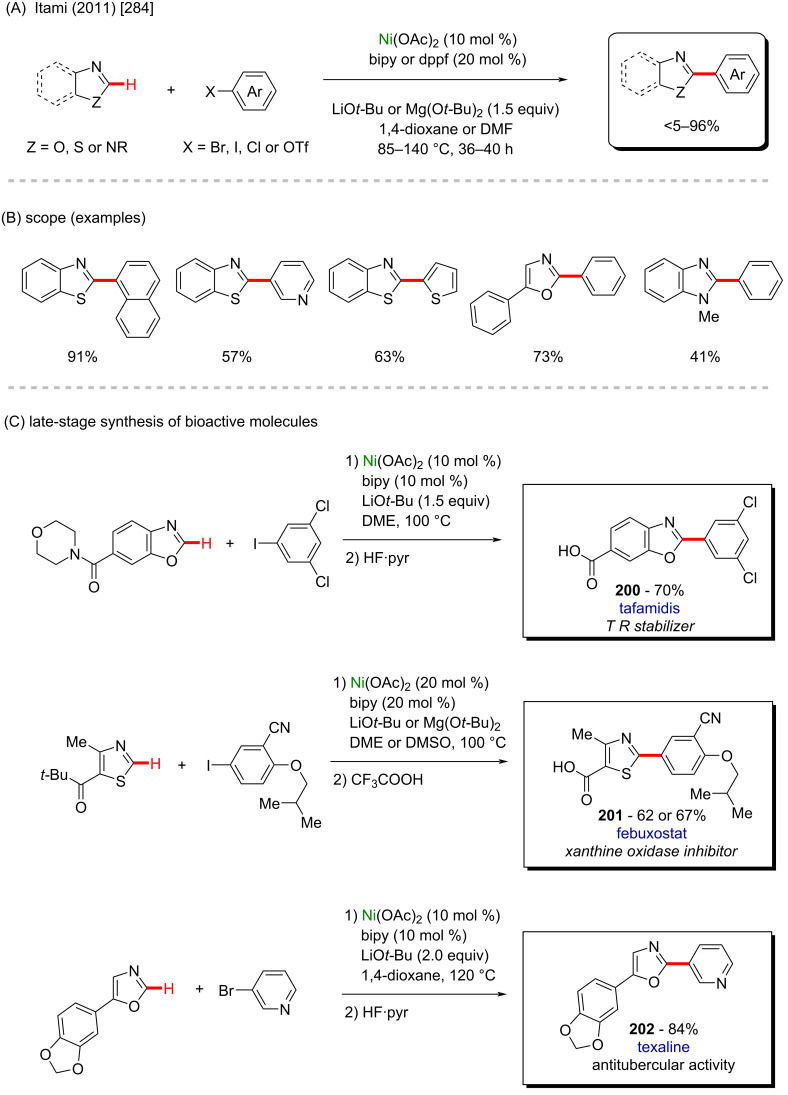
(A and B) Another notable example of a nickel-catalyzed C–H arylation of azole derivatives; (C) late-stage application to the synthesis of potent drugs.

Already in 2011, Itami and co-workers explored the catalytic activity of nickel(II) acetate in a C–H arylation of azole derivatives (Scheme 63A) [284]. Since then, this method led to the formation of several arylated products in good yields (Scheme 63B). The same methodology was applied in a late-stage functionalization towards the synthesis of three important drugs (Scheme 63C): tafamidis (**200**) (used to treat amyloidosis [285]), febuxostat (**201**) (used to treat gout [286]), and texaline (**202**) (presents antitubercular activity [287]).

**Scheme 64:**
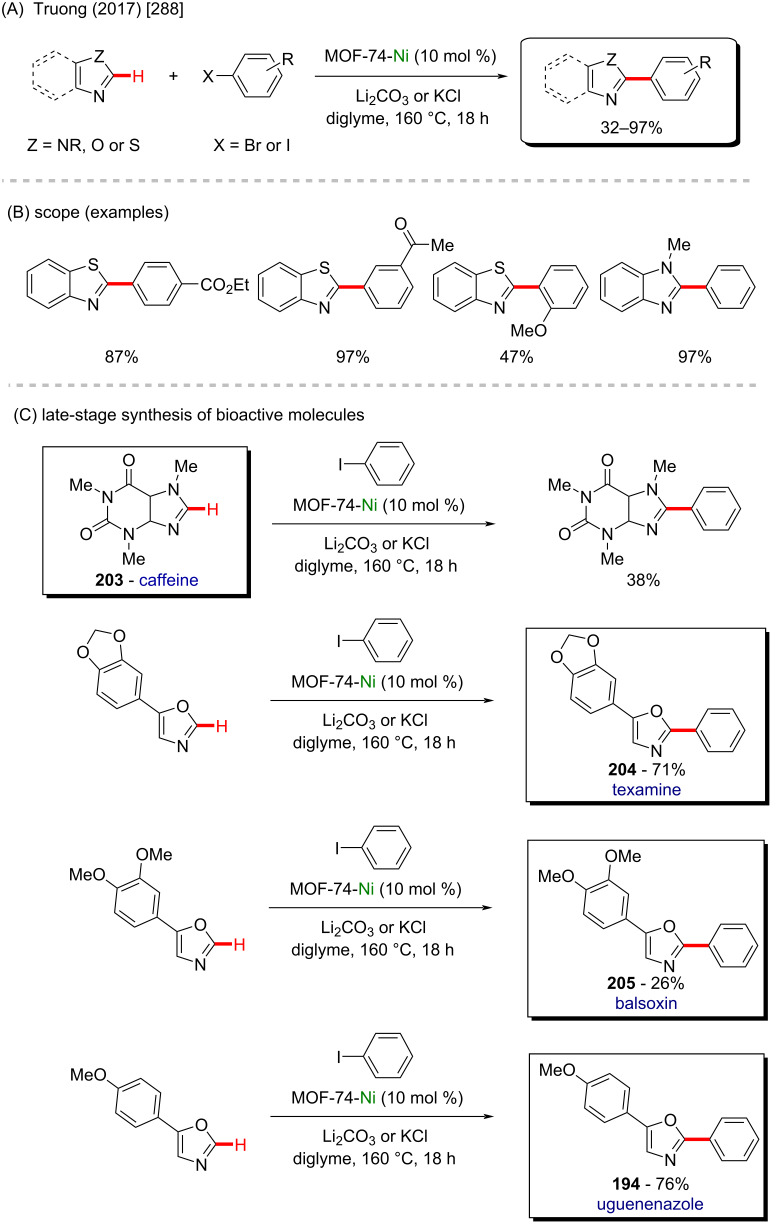
(A and B) Nickel-based metalorganic framework (MOF-74-Ni)-catalyzed C–H arylation of azole derivatives; (C) application to the synthesis of compounds with biological activity.

A similar methodology was explored by Truong and co-workers in 2017 [288]. In this work, a nickel-based metalorganic framework (MOF-74-Ni) mediated a C–H arylation process of several azole derivatives (Scheme 64A). The desired arylated products were obtained in good yields, including compounds known to present important biological activities (Scheme 64B and C) such as the caffeine derivative from **203** [289], texamine (**204**) [290], balsoxin (**205**) [290], and the previously mentioned uguenenazole (**194**) [276].

**Scheme 65:**
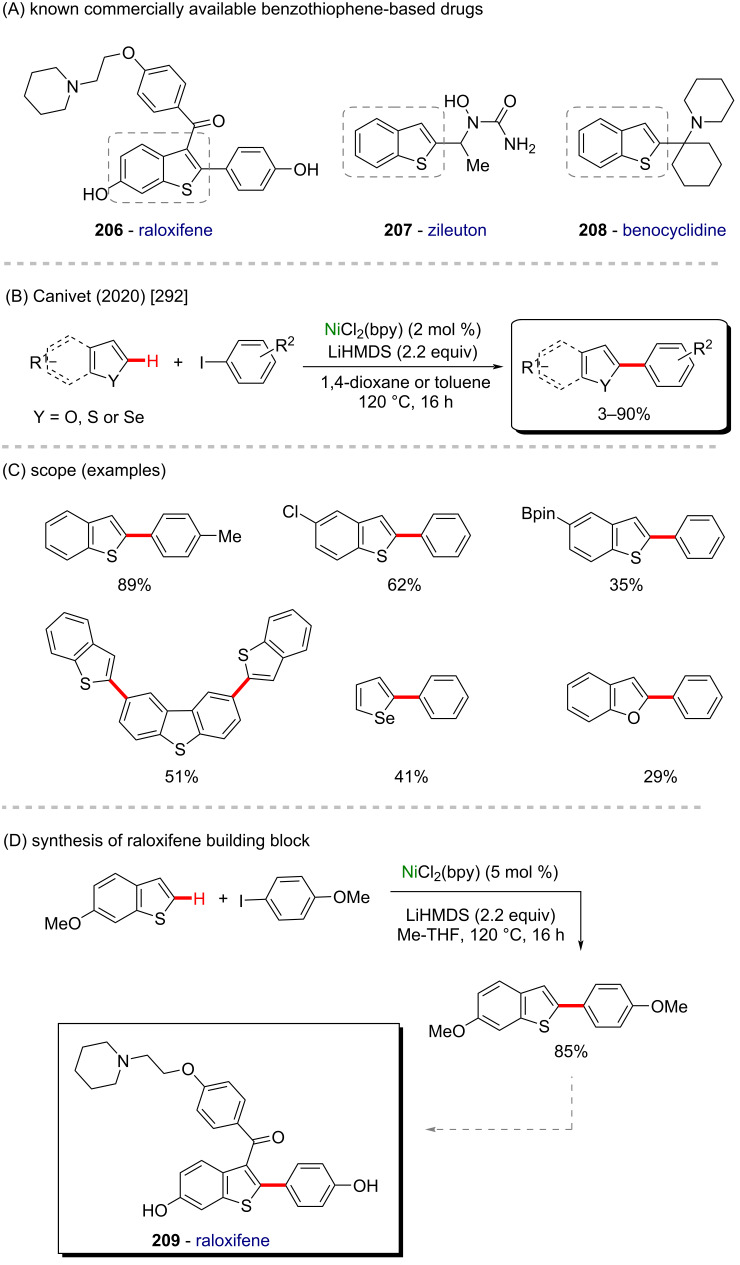
(A) Known commercially available benzothiophene-based drugs; (B and C) nickel-catalyzed C–H arylation of benzothiophene derivatives; (D) synthesis of a raloxifene building block.

Currently, there are several examples of commercially available drugs containing a benzothiophene unit in their structures (**206**–**208**, Scheme 65A) [291]. Following this idea, it is worthy to mention here an important work published by Canivet and co-workers in 2020 [292], in which the authors described a regioselective C–H arylation of benzothiophene derivatives. Through this method, a large variety of arylated products were successfully obtained in plausible yields (Scheme 65B and C). A further study of this methodology led to the synthesis of an essential building block of raloxifene (**209**) (Scheme 65D), a known anticancer drug [293].

**Scheme 66:**
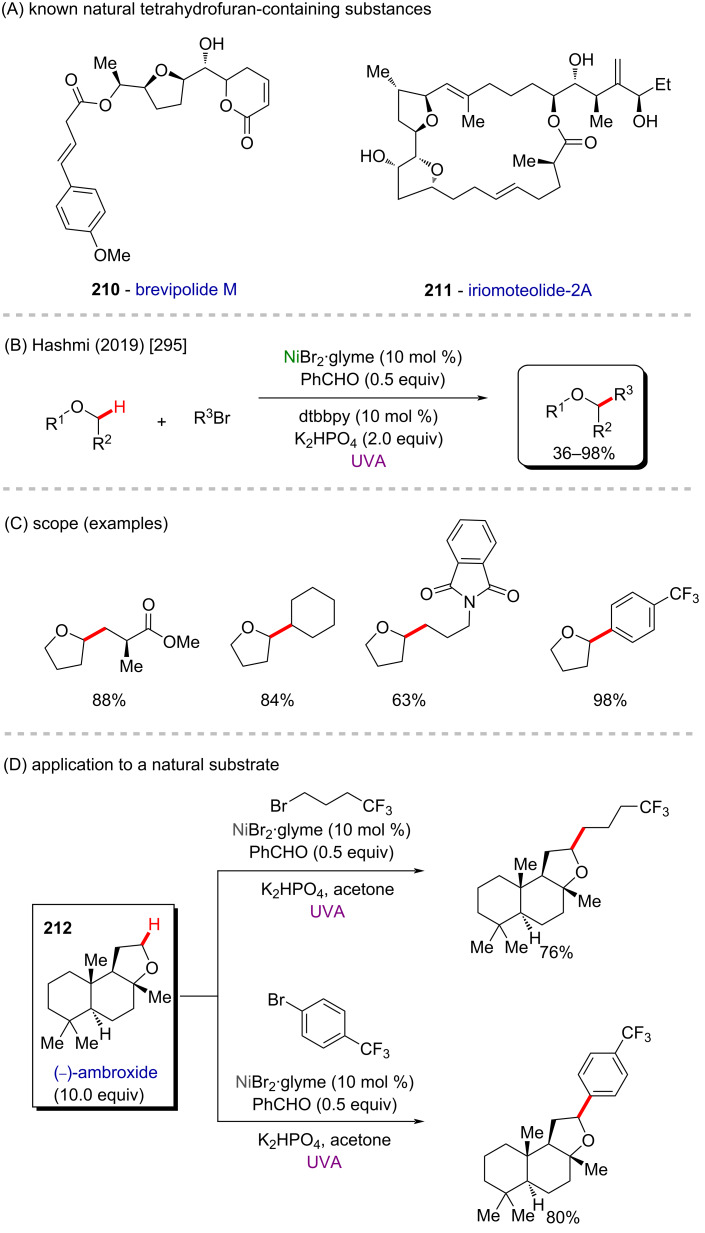
(A) Known natural tetrahydrofuran-containing substances; (B and C) nickel-catalyzed photoredox C(sp^3^)–H alkylation/arylation; (C) late-stage modification of (−)-ambroxide (**212**).

Some important bioactive natural molecules (**210** and **211**) present at least one tetrahydrofuran unit in their structure, so reactions that promote a structural modification of this organic class of molecules are extremely valuable (Scheme 66A) [294]. Hashmi and co-workers published in 2019 a notable work in which they described a nickel-catalyzed photoredox C(sp^3^)–H alkylation/arylation process [295]. In this work, NiBr_2_·glyme was used as a nickel source in the presence of benzaldehyde as a photosensitizer, and 4,4′-di-*tert*-butyl-2,2′-dipyridyl (dtbbpy) as the ligand under UVA light irradiation (Scheme 66B). The desired tetrahydrofuran-based products were obtained in good yields (Scheme 66C). Subsequently, the developed procedure was successfully applied as a late-stage modification of the natural substance (−)-ambroxide (**212**) (Scheme 66D), a compound excreted by the sperm whale (*Physeter catodon*) with a direct effect on the odor sensibility of women during the menstrual cycle [296].

**Scheme 67:**
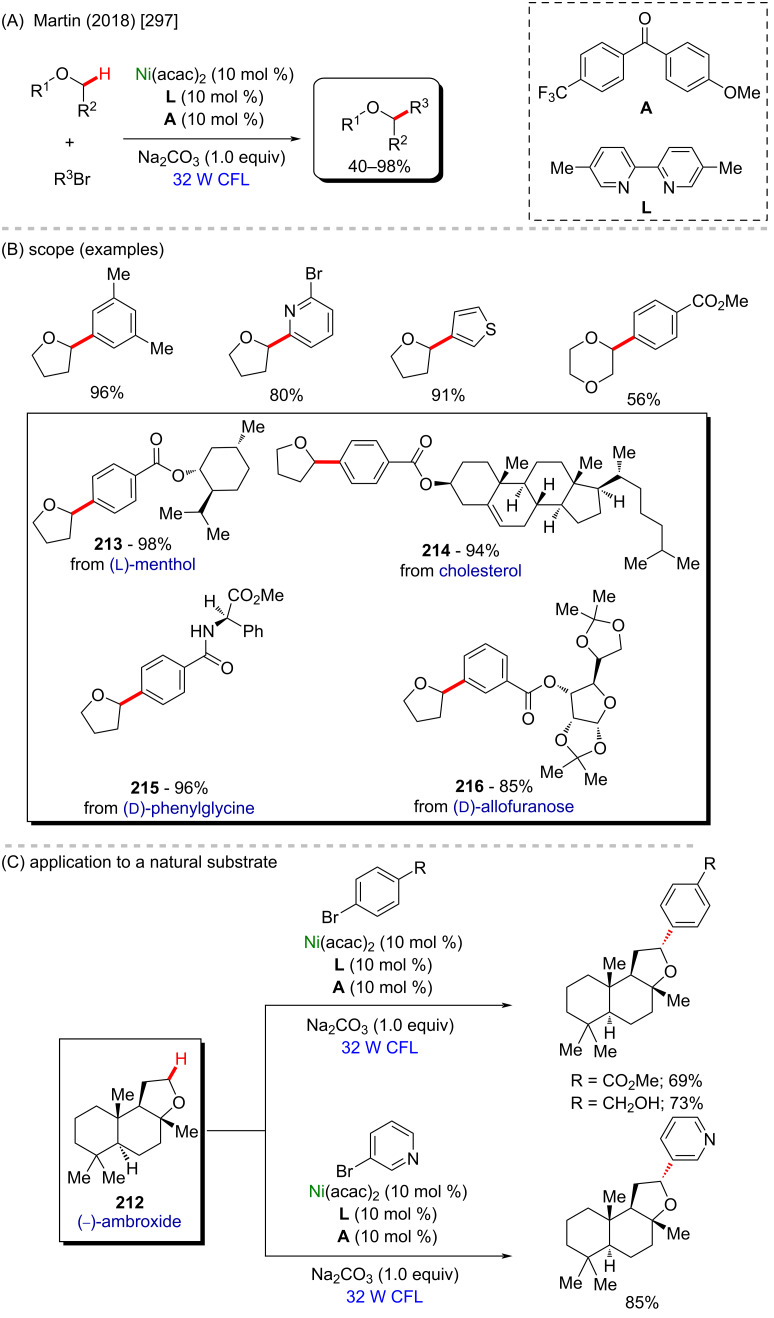
(A and B) Another notable example of a nickel-catalyzed photoredox C(sp^3^)–H alkylation/arylation; (C) late-stage application to (−)-ambroxide (**212**).

Using a similar methodology, Martin and co-workers described in 2018 another nickel/photocatalyzed C–H arylation/alkylation [297] but using Ni(acac)_2_ as a catalyst, in the presence of an asymmetric benzophenone derivative (**A**) and a bipyridine ligand (**L**) (Scheme 67A). The authors described the successful synthesis of many activated products (**213**–**216**), including examples of bromides derived from bioactive compounds, that afforded the final products in excellent yields (Scheme 67B). Similar to what was discussed in the previous example, the authors also performed a late-stage functionalization of (−)-ambroxide (**212**), that led to three arylated products with potential biological activities (Scheme 67C).

**Scheme 68:**
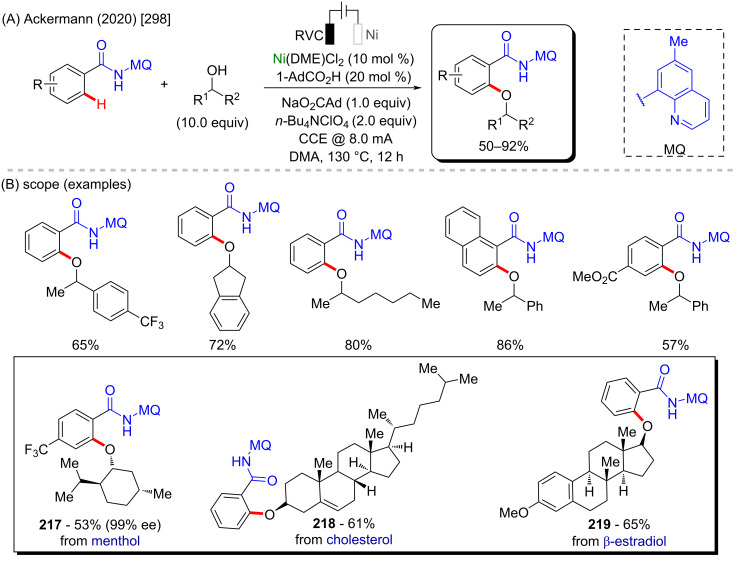
(A) Electrochemical/nickel-catalyzed C–H alkoxylation; (B) achieved scope, including three using natural compounds as coupling partners.

Another nickel-catalyzed C–H activation was described by Ackermann and co-workers in 2020 [298], in which an *ortho*-directed C–H alkoxylation was mediated in an electrochemical environment (Scheme 68A). Using this methodology, the authors achieved several interesting alkoxylated products in moderate to good yields. Amongst the obtained substances, it is worth to mention the successful late-stage functionalization using natural alcohols, such as menthol, cholesterol, and β-estradiol, from which the final products **217**–**219** were obtained in 53%, 61%, and 65% yields, respectively, without compromising the chiral centers already present in the original natural substances (Scheme 68B).

**Scheme 69:**
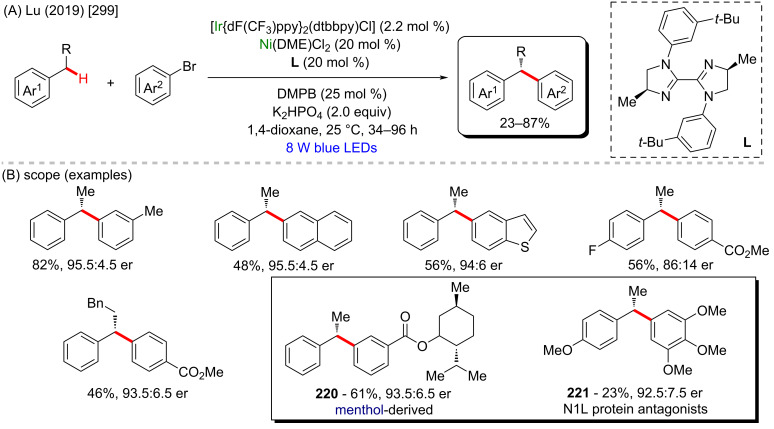
(A) Enantioselective photoredox/nickel catalyzed C(sp^3^)–H arylation; (B) achieved scope, including two potential bioactive compounds.

In 2019, Lu and co-workers reported a unique enantioselective photoredox/nickel-catalyzed C(sp^3^)–H arylation method in the presence of a chiral ligand (Scheme 69A) [299]. By this method, several chiral 1,1-diarylethane products were obtained in good enantioselectivities. The applicability of this method was defended by the authors by the synthesis of a menthol-derived product **220**, and another compound (**221**) already known to present antagonist activity against the N1L protein (Scheme 69B) [300], therefore a potential anti-variola agent.

**Scheme 70:**
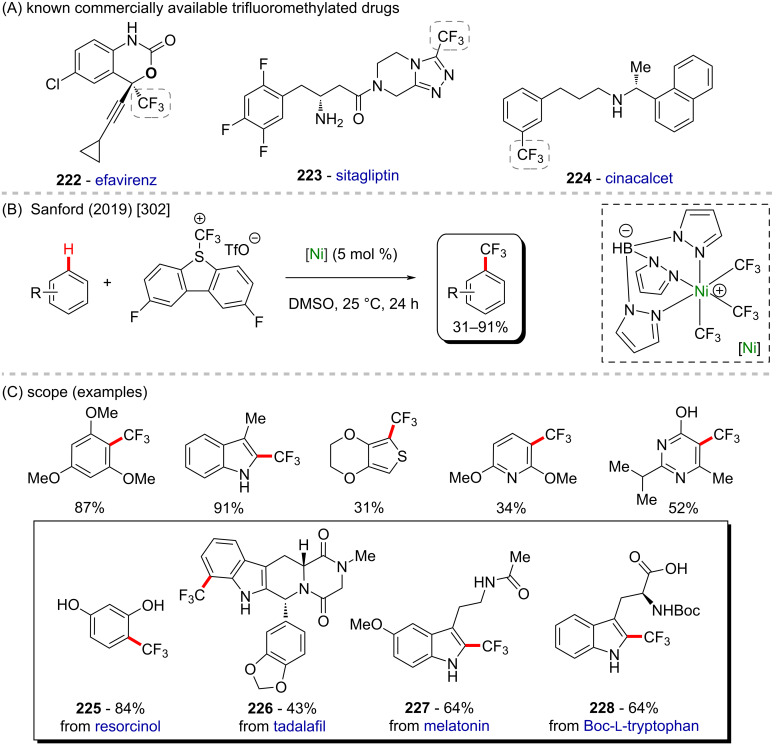
(A) Known commercially available trifluoromethylated drugs; (B and C) nickel-catalyzed C–H trifluoromethylation.

Some important commercially available drugs have a trifluoromethane unit, such as efavirenz (**222**), sitagliptin (**223**), and cinacalcet (**224**) (Scheme 70A) [301]. Nickel catalysis can also be used to mediate a C–H trifluoromethylation, as was well-exemplified in the work published by Sanford and co-workers in 2019 [302]. In this work, the authors used 2,8-difluoro-5-(trifluoromethyl)-5*H*-dibenzo-[*b*,*d*]thiophen-5-ium trifluoromethanesulfonate as a trifluoromethane source in combination with a robust nickel complex as catalyst (Scheme 70B). In the scope study, the authors not only described the successful synthesis of several activated compounds (**225**–**228**), but they also used known biologically active substances as substrates for this methodology (Scheme 70C).

**Scheme 71:**
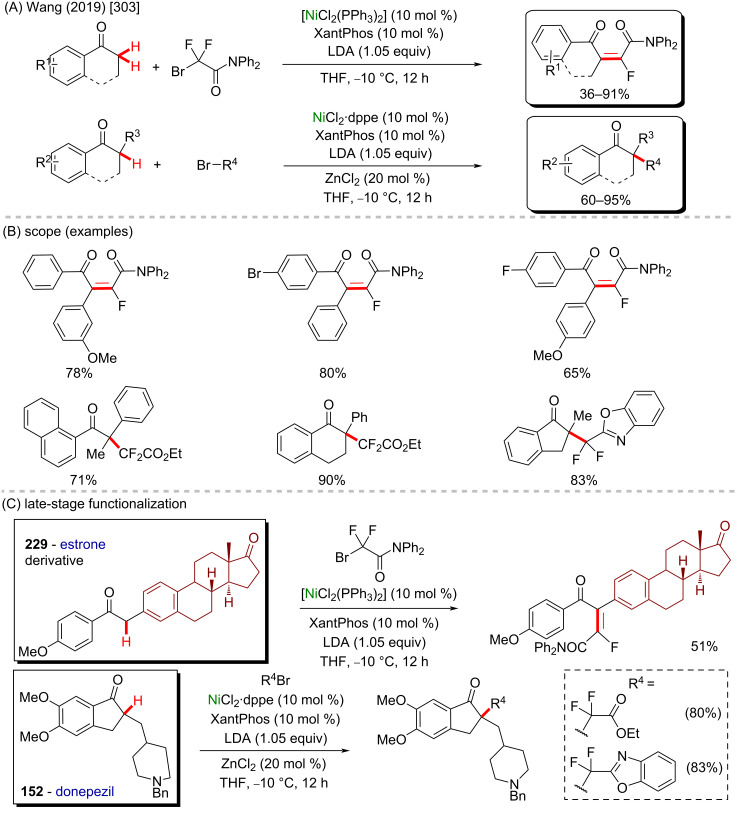
(A and B) Stereoselective nickel-catalyzed C–H difluoroalkylation; (C) late-stage functionalization of bioactive molecules.

In the same year, Wang and co-workers published a work describing a stereoselective nickel-catalyzed C–H difluoroalkylation for the formation of both tetrasubstituted alkenes and quaternary difluoroalkylated products [303]. The distinction of which reaction takes place relies not only on the structure of the coupling partner, but also on the structure of the reactant itself since there are two removable hydrogen atoms necessary for the tetrasubstituted alkene to be formed (Scheme 71A and B). The method was also applied in a late-stage functionalization of bioactive molecules such as an estrone derivative (**229**) and donepezil (**152**) [219]. In both cases, the final products were successfully formed in good yields (Scheme 71C).

The versatility of nickel metal has made it also a considerably emerging metal. From the previous examples, it is clear that this metal plays an important role not only in usual C–H processes, but even further, for the synthesis of bioactive molecules. Various important C–H functionalizations can be performed, including alkoxylations and trifluoromethylations.

### Copper-catalyzed C–H activation

Copper is an abundant, non-expensive, and relatively nontoxic transition metal, and therefore copper-mediated reactions for direct functionalization of C–H bonds have emerged as promising tools for development of more sustainable methods for the synthesis of fine chemicals. Reactions involving copper-mediated C–H activations allow for a direct insertion of functional groups in unreactive C–H bonds and the formation of carbon–carbon bonds without the requirement of prefunctionalized substrates, which allow for shorter synthetic routes or late-stage modifications of structurally complex compounds [304–312]. However, the development of efficient methods can be challenging due to the requirement of directing groups and control of selectivity. In the case of reactions for applications in the synthesis of bioactive compounds, an additional challenge is the frequent presence of heterocycles containing metal-coordinating atoms, such as nitrogen and sulfur, that can compete with ligands and directing groups for coordination with the metal, leading to poor selectivity [313]. On the other hand, such heterocyclic moieties present in bioactive compounds, or their synthetic intermediates may eventually play the role of a directing group, thus providing an opportunity for convenient and straightforward transformations based on metal-mediated C–H activation [314]. Many methods potentially useful for the synthesis or modification of bioactive compounds based on copper-promoted activation of C–H bonds in (hetero)arenes have been reported in the last years, including methods for the formation of C–C [304–312], C–X [315–319], C–N [305,320–324], C–O [325–330], and C–S [324,331–332] bonds. Some of these methods are highlighted herein. It is worth mentioning that the covered examples were selected based on evidence for reaction mechanisms involving a metal-mediated C–H activation through the formation of cyclometalated species and applications in medicinal chemistry. The field of copper-mediated functionalization of C–H bonds is much broader and has been covered by excellent reviews [333–336].

Nitrogen-containing motifs, especially heteroaromatic rings, amines, and amides, are usually encountered in bioactive compounds, such as natural products, particularly alkaloids, and marketed drugs. For this reason, convenient methods for C–N bond formation reactions are particularly useful in medicinal chemistry and drug development and cost-effective and sustainable methods are required for industrial applications. Amination reactions involving a copper-mediated C(sp^2^)–H bond activation were first achieved in 2006 by Yu and co-workers [315] and have proven useful for the synthesis of a wide range of anilines and heteroanilines, including drug intermediates.

**Scheme 72:**
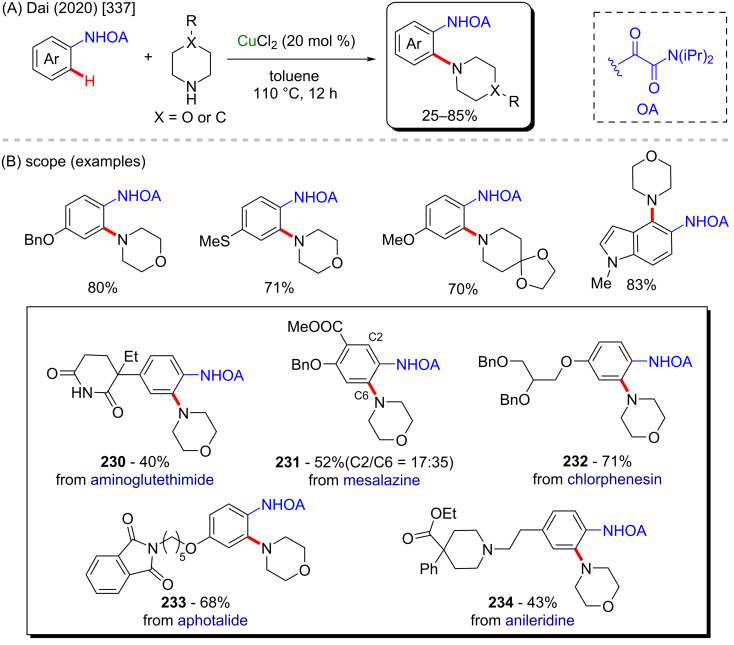
(A) Cu-mediated *ortho*-amination of oxalamides; (B) achieved scope, including derivatives obtained from bioactive compounds.

A method for direct *ortho*-coupling of amines with anilines using a removable directing group and molecular oxygen as oxidant was recently reported by Wu and co-workers [337]. In this work, the introduction of cyclic secondary amines in the *ortho*-position through Cu-mediated C–H activation was achieved by using oxalamide as a weakly coordinating directing group (Scheme 72A), which can be removed after the reaction under basic conditions to deliver the corresponding *o*-aminoanilines. The amination reaction is performed with Cu(I), which is oxidized by oxygen to Cu(II). The mechanism was suggested to involve a single-electron transfer step since radical scavengers completely inhibited the reaction. The method was showed to be suitable for a wide range of oxalamides as substrates whereas secondary amines, such as morpholine and piperazine, were used. Notably, the mild reaction conditions were compatible with many functional groups and with substrates bearing various heteroaromatic rings, which are common motifs in bioactive compounds. These features allowed the reaction to be applied for a late diversification of drugs (compounds **230**–**234**) (Scheme 72B).

**Scheme 73:**
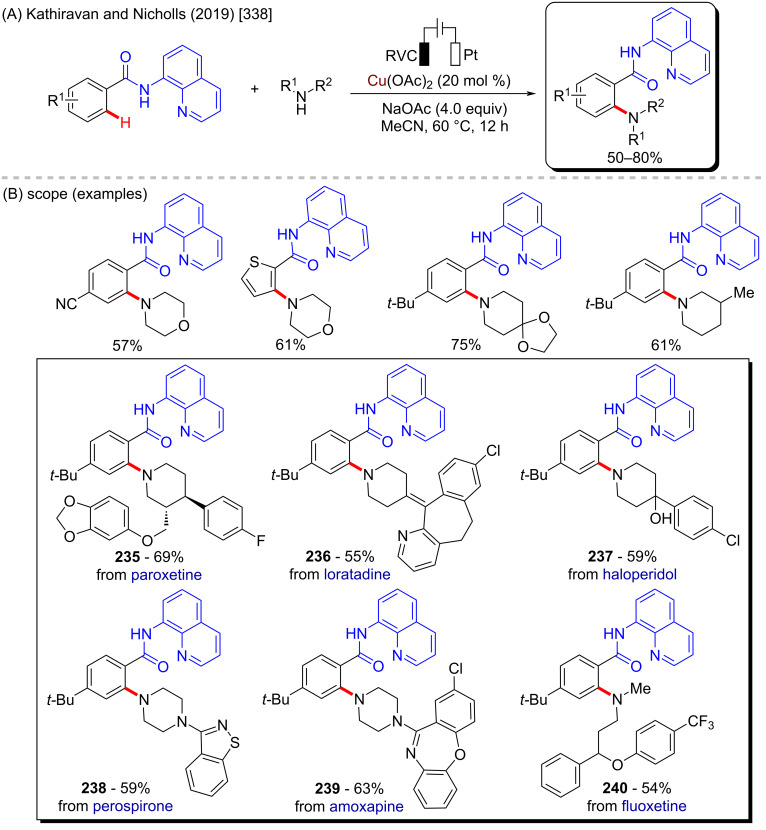
(A) Electro-oxidative copper-mediated amination of 8-aminoquinoline-derived amides; (B) achieved scope, including derivatives obtained from bioactive compounds.

A method for the C(sp^2^)–N bond formation through a copper-mediated C(sp^2^)–H bond activation without the requirement of an external oxidant was developed by Kathiravan and Nicholl’s group [338]. Using a Pt plate and RVC electrodes and Cu(II) salts, a Cu-mediated electro-oxidative C–H/N–H cross coupling of 8-aminoquinoline-derived aryl amides and secondary amines could be performed (Scheme 73A). The reaction takes place under mild conditions and solely molecular hydrogen is released as a byproduct. This is in contrast to undesired metal products formed in reactions using stoichiometric external oxidants. The method was found feasible with several 8-aminoquinoline-derived amides and several secondary amines and the broad scope and chemoselectivity were corroborated by applying drugs (compounds **235**–**240**) as the amine counterparts (Scheme 73B).

**Scheme 74:**
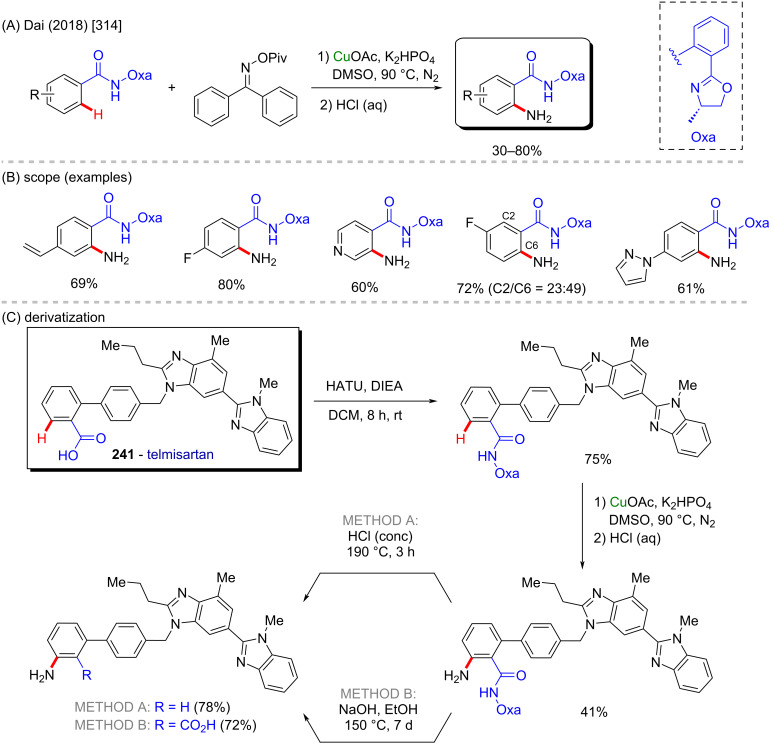
(A and B) Cu(I)-mediated C–H amination with oximes; (C) derivatization using telmisartan (**241**) as starting material.

Primary anilines are useful building blocks in medicinal chemistry since they can be used as intermediates in the synthesis of heterocycles and are often a structural motif in bioactive compounds [69–71]. For this reason, the direct introduction of a free amino group into an arene moiety through C(sp^2^)–H amination to give primary anilines is a valuable transformation. However, selectivity issues and the strength of the metal–NH_2_ bond make the development of methods for such reactions a challenging task. For this purpose, the use of oximes as amino group sources and Cu(I) salts were reported to promote the *ortho*-amination of amides via metal-mediated C(sp^2^)–H activation (Scheme 74A and B) [314]. The reaction was found to work well with an amide oxazoline group as a directing group, CuOAc as Cu(I) salt, and K_2_HPO_4_ as an additive giving aryl imines, which could then be hydrolyzed in situ under acid conditions to give the corresponding primary anilines. Because oximes also acted as oxidants, no additional external oxidant was required. The method was found to present a broad scope and displayed high chemoselectivity, being feasible for the *ortho*-amination of a wide variety of aryl amides bearing various functional groups. It is noteworthy that the reaction also could be performed with heteroaryl amides featuring *N*-containing heteroaromatic rings to give the products in moderate to good yields, since these are challenging substrates due to the possibility of undesired coordination to the metal. The oxazoline amide group could also be further converted to a carboxyl group by treatment with a base or completely removed through decarboxylation under acid conditions at high temperature. The usefulness of the method was demonstrated by the introduction of an amino group into the drug telmisartan (**241**). Taking advantage of a carboxyl group in the molecule, the directing group could be introduced through an amidation reaction. The Cu(I)-mediated C–H amination was then performed and followed either by amide hydrolysis to give the original carboxyl group or by a complete removal of the directing group (Scheme 74C).

**Scheme 75:**
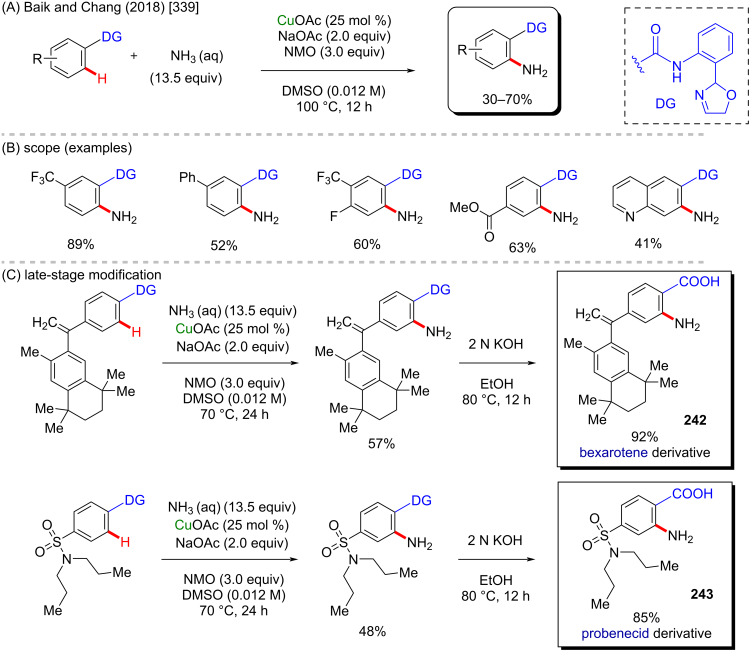
(A and B) Cu-mediated amination of aryl amides using ammonia; (C) late-stage modification of probenecid (**242**) and bexarotene (**243**).

Although ammonia strongly coordinates to metals, leading to catalyst poisoning, the introduction of an amino group into the *ortho*-position of oxazoline-derived aryl amides via C–H activation using aqueous ammonia was accomplished by aid of a soft, low-valent Cu(I) species (Scheme 75A and B) [339]. Preliminary studies suggested that the reaction mechanism involves disproportionation of the Cu(I) species leading to the in situ formation of a Cu(III) species, which results in an increased acidity of a copper–amido intermediate, thus allowing for the deprotonation of copper-bound ammonia and a reductive elimination step to give the product. Noteworthy, attempts to perform the reaction using other transition metals, such as Pd, Pt, Co, Rh, and Ir were unsuccessful. The reaction was optimized with the use of acetate as the base to assist deprotonation and NMO as an oxidant, so that CuOAc could be used as the Cu(I) source in a substoichiometric amount. The method was found to present a broad scope and selectivity and was proven useful for the late-stage modification of drugs, such as probenecid (**242**) and bexarotene (**243**) (Scheme 75C).

**Scheme 76:**
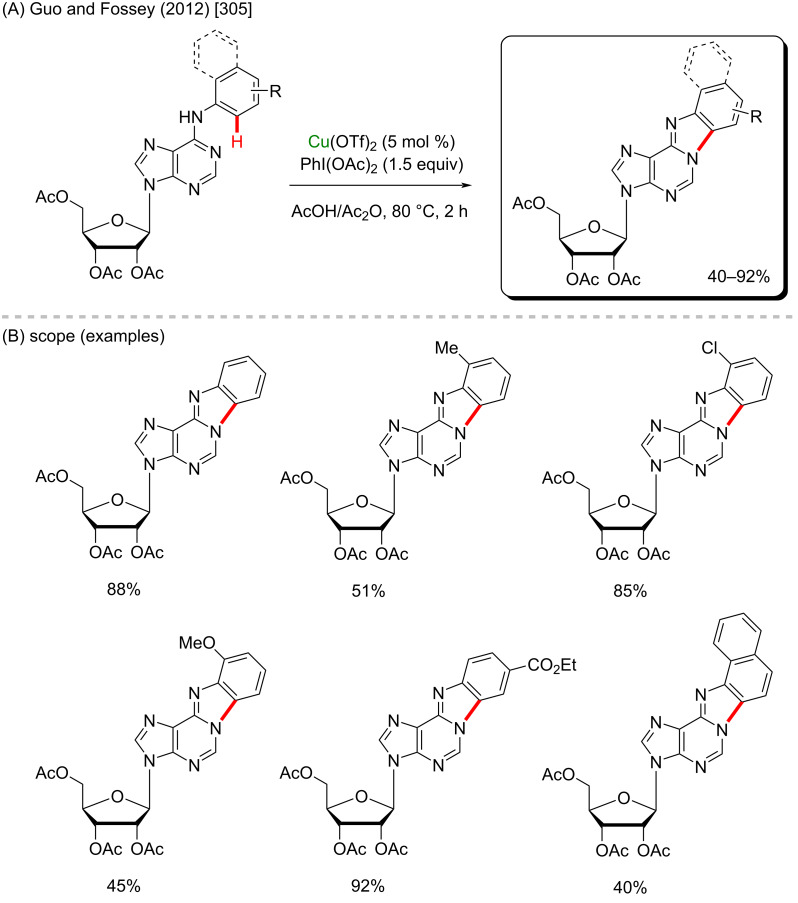
(A and B) Synthesis of purine nucleoside analogues using copper-mediated C(sp^2^)–H activation.

The copper-mediated C–H activation for C–N bond formation is useful in the synthesis of *N*-containing heterocycles commonly encountered in bioactive compounds, such as pyrido[1,2-*a*]benzimidazoles [320], 1*H*-indazoles and 1*H*-pyrazoles [322], among others. For instance, a method for the modification of 6-anilinopurine nucleosides through copper-mediated C(sp^2^)–H activation and intramolecular amination was reported to synthesize modified nucleosides, which are useful scaffolds in the design of antiviral drugs. The reaction could be performed with 6-anilinopurine nucleosides taking advantage of a purine ring as directing group without cleaving the fragile purine–glycoside bond (Scheme 76) [305].

**Scheme 77:**
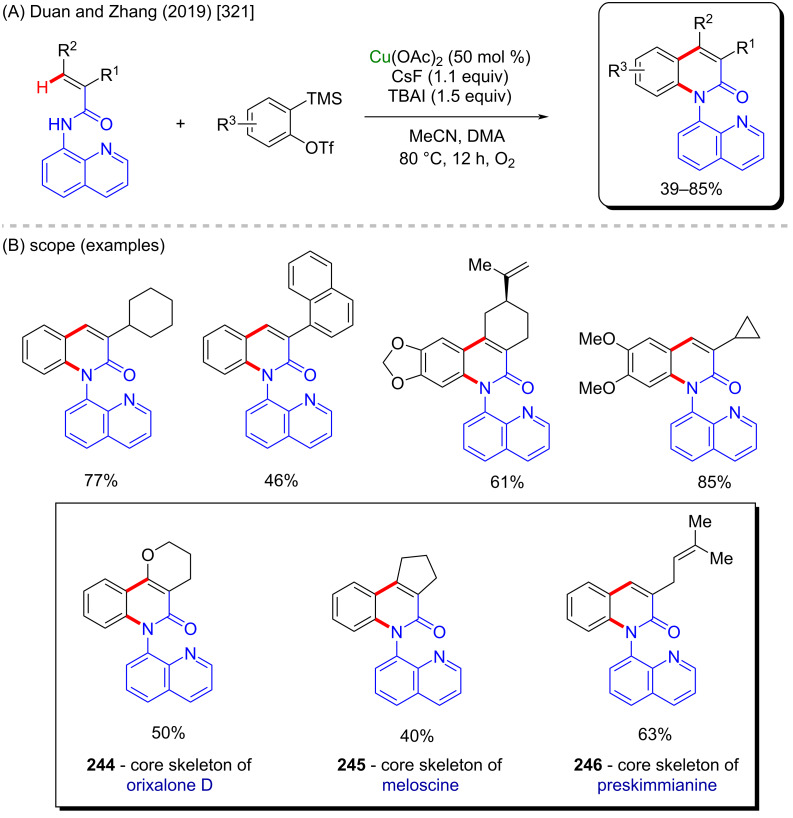
(A) Copper-mediated annulation of acrylamide; (B) achieved scope, including the synthesis of the core skeletons of three bioactive molecules.

Another example of the usefulness of a copper-mediated C–H activation reaction was reported by Duan, Zhang and co-workers in the synthesis of polycyclic 2-quinolinones, which are scaffolds present in bioactive alkaloids, such as orixalone D, mertinellic acid and meloscine (Scheme 77A) [321]. By using Cu(OAc)_2_ in substoichiometric amount, O_2_ as oxidant, and Kobayashi aryne precursors, the coupling of acrylamides derived from 8-aminoquinoline and arynes could be achieved with moderate to good yields, including three core skeletons of three important bioactive molecules (compounds **244**–**246**, Scheme 77B).

**Scheme 78:**
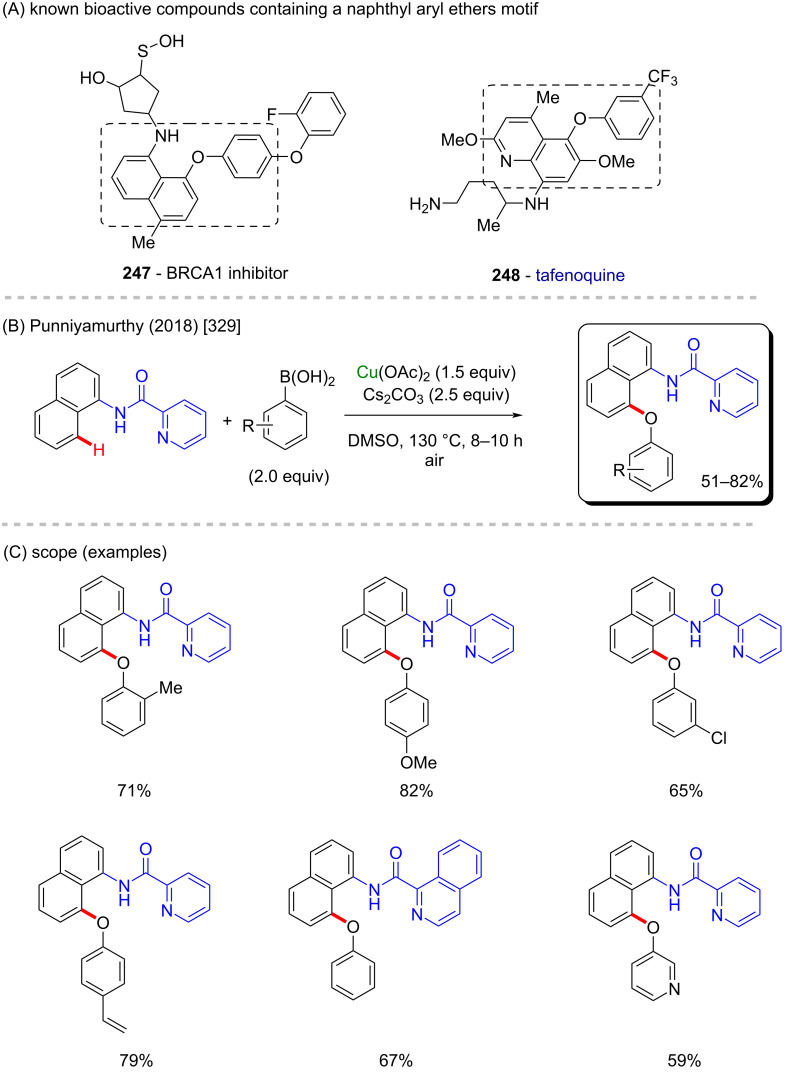
(A) Known bioactive compounds containing a naphthyl aryl ether motif; (B and C) copper-mediated etherification of naphthylamides through C–H bond activation.

Copper has been reported to be useful in C–O bond-formation reactions involving C(sp^2^)–H activation for the introduction of tosyl-, hydroxy-, acyloxy-, alkoxy-, and aryloxy groups into arenes [324–328,330]. A copper-based method was reported by Punniyamurthy and co-workers who applied a copper-based method to the synthesis of naphthyl aryl ethers, which are motifs featured by bioactive compounds, such as the antimalarial drug tafenoquine (**248**) (Scheme 78A) [329]. The naphthyl aryl ethers were obtained from picolinic acid-derived naphthylamides, arylboronic acids, and water as an oxygen source. Picolinamide was found to be a proper directing group, which was ascribed to its ability to act as *N*,*N*-bidentate ligand with copper and the relative acidity of its NH group (Scheme 78B and C). Mechanistic studies confirmed water, which is released into the reaction medium by reaction of the base with acetic acid, as the oxygen source. They suggested the reaction to involve an intramolecular C–H activation through cyclometalation and the formation of a Cu(III) species. The method was shown to present a broad scope and chemo- and regioselectivity. Also, the directing group could be removed by hydrolysis with an ethanolic solution of NaOH under reflux.

**Scheme 79:**
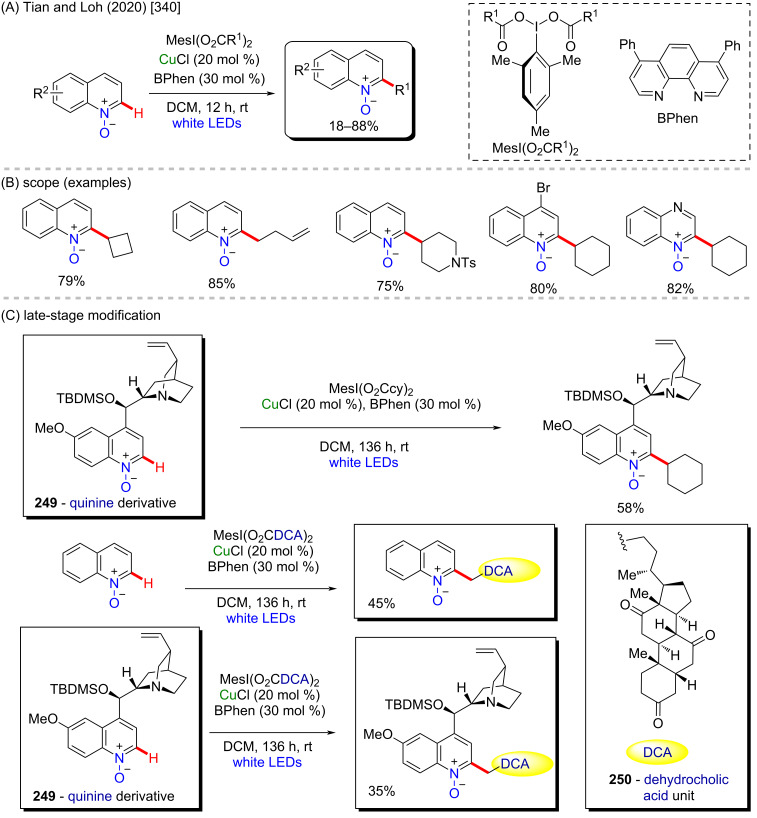
(A and B) Cu-mediated alkylation of *N*-oxide-heteroarenes; (C) late-stage modification.

Reactions for C–C-bond formation involving copper-mediated C–H bond activation have been reported and some of them have shown to be useful in medicinal chemistry. For instance, Tian, Loh and co-workers reported a method for the direct and *ortho*-selective alkylation of *N*-oxide arenes under mild conditions with a copper-complex as a photocatalyst (Scheme 79A and B) [340]. In this method, several hypervalent iodine carboxylates prepared from non-expensive raw materials were used as alkylating agents. The reaction took place under visible light irradiation and was proposed to involve the photocatalytic production of an alkyl radical and metallization of position C-2 of the *N*-oxide heteroarene. The reaction could be carried out with diverse *N*-oxide arenes and alkylating agents affording the products in moderate to good yields. The usefulness and chemoselectivity of the method were demonstrated by the coupling of a quinine derivative **249** and dehydrocholic acid (**250**) (Scheme 79C).

**Scheme 80:**
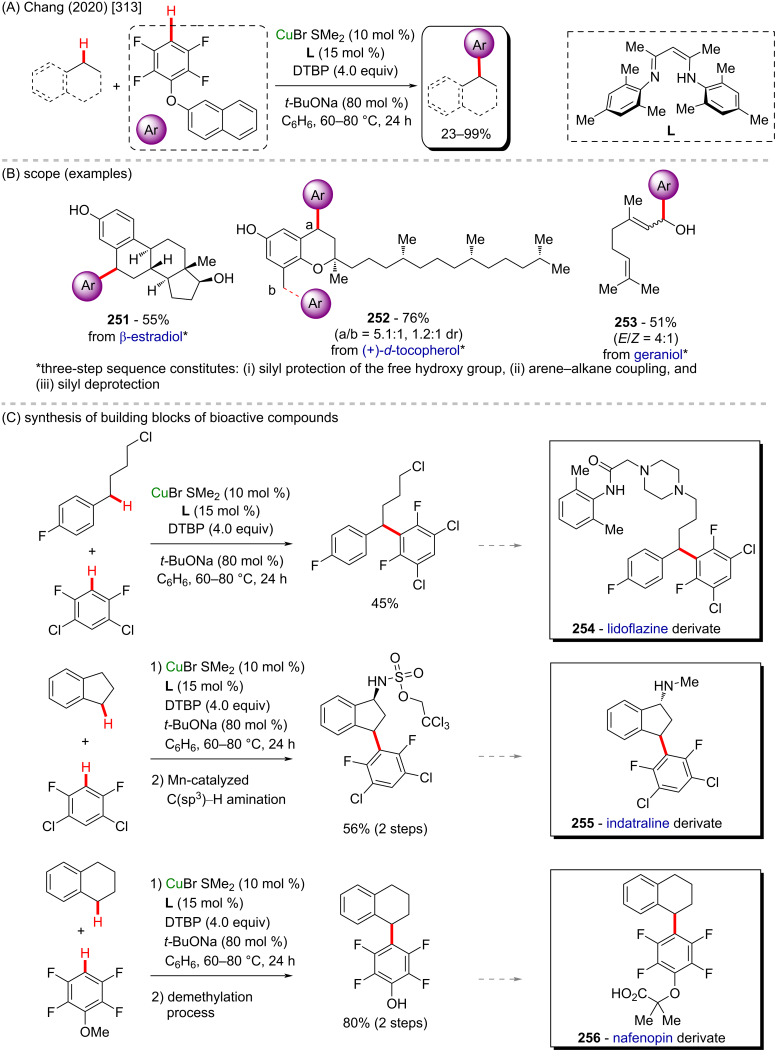
(A) Cu-mediated cross-dehydrogenative coupling of polyfluoroarenes and alkanes; (B) scope from known bioactive substrates; (C) synthesis of building blocks for bioactive compounds.

A method for the direct alkylation of polyfluoroarenes using non-functionalized alkanes was recently reported. The reaction requires the activation of both the C(sp^2^)–H bond of the fluoroarene and the C(sp^3^)–H bond of the alkane, thus it is challenging due to the possibility of homocoupling and overall alkylation. A cross-dehydrogenative coupling of polyfluoroarenes and alkanes was achieved by the use of a Cu(I) salt and a β-ketimide ligand in the presence of di-*tert*-butyl peroxide as the oxidant (Scheme 80A) [313]. The choice of a proper ligand was suggested to be a crucial issue to achieve selectivity and facilitate the reaction due to interactions with the arene substrates. The reaction was found to present a broad scope regarding the obtained drug derivatives and the synthesis of the fluorinated drug precursors **251**–**256** (Scheme 80B and C). In addition, the method was carried out in a decagram scale to furnish the polyfluorinated biaryl product with 80% yield from the coupling of 2,3,5,6-tetrafluoroanisole and ethylbenzene, which could be further converted to different products through hydrodefluorination by nucleophilic aromatic substitution of fluorine atoms.

**Scheme 81:**
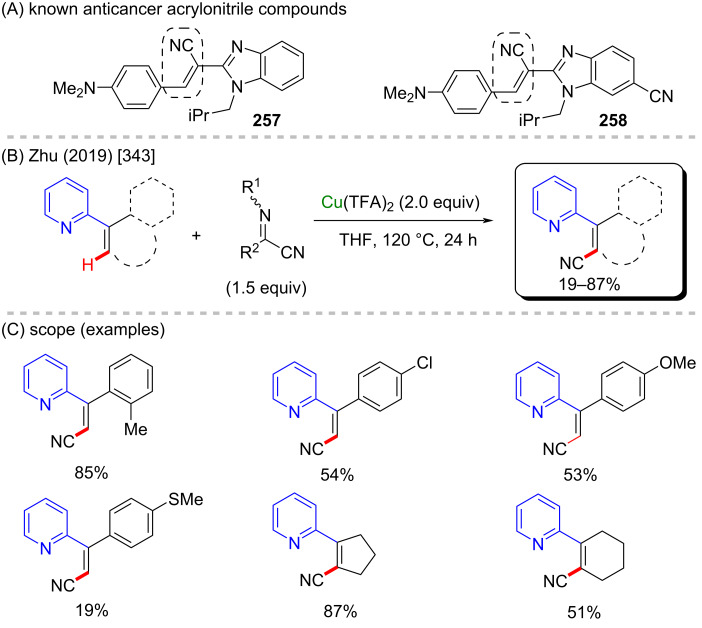
(A) Known anticancer acrylonitrile compounds; (B and C) Copper-mediated cyanation of unactivated alkenes.

The acrylonitrile products **257** and **258** have shown potential anticancer activity against eight different cell lines (Scheme 81A) [341]. The direct cyanation of alkenes and (hetero)aromatic compounds are useful reactions that have been reported to be accomplished through copper-mediated activation of C(sp^2^)–H bonds [342–344]. Such cyanation reactions are attractive for medicinal chemistry purposes, since nitrile groups are present in drugs, such as nivapidine and entacapone, and can also give access to other medicinally relevant functional groups, such as amines, amides, carboxylic acids, and *N*-containing heterocycles. A method for obtaining acrylonitriles through copper-mediated activation of alkene C–H bonds was recently reported for the first time by Zhu and co-workers (Scheme 81B and C) [343]. The reaction could be performed by using iminonitrile as a nitrile source and a pyridine moiety as a directing group to give the cyanated compounds with moderate to good yields.

**Scheme 82:**
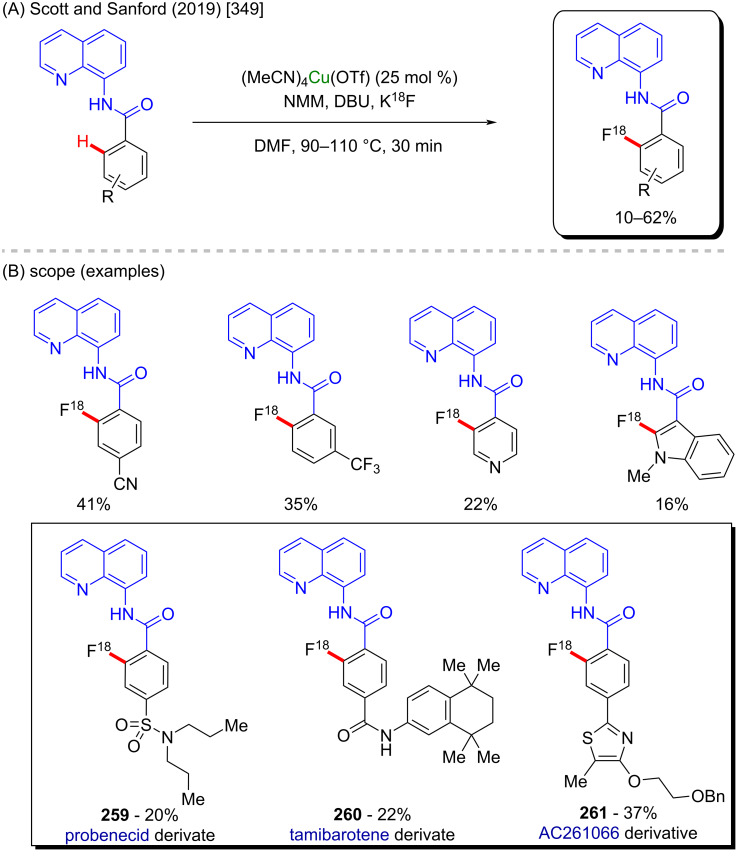
(A) Cu-mediated radiofluorination of 8-aminoquinoline-derived aryl amides; (B) achieved scope, including three derivatives of bioactive molecules.

Methods for introducing halogens into organic compounds are useful in organic synthesis, since halogen atoms can be further replaced by other functional groups, so that halogenated compounds are often required as synthetic intermediates [345–347]. Moreover, many bioactive compounds feature halogen atoms [348]. The halogenation of arenes has been accomplished through the Cu-mediated activation of C(sp^2^)–H bonds. An example of the usefulness of this transformation for drug development was provided by Scott and Sanford, who developed a method for radiofluorination of arenes mediated by Cu(I) salts and K^18^F as a source of nucleophilic radioactive fluorine (Scheme 82A) [349]. The reaction was accomplished using NMM, DBU as the base additive and with 8-aminoquinoline as a directing group, so that an *ortho*-selective radiofluorination of 8-aminoquinoline-derived arylamide could be achieved. The chemoselectivity and broad scope allowed the reactions to be employed for late-stage radiofluorination of the bioactive compounds **259** and **260**, thus furnishing ^18^F-containing compounds which are useful for positron emission tomography (PET) imaging (Scheme 82B). The method could be adapted for automated synthesis and further amide hydrolysis to remove the directing group. The developed protocol was found to be useful for the ^18^F-labeling of AC261066 (**261**), an agonist of the retinoic acid receptor β (RAR-β).

The cited examples make is easy to understand why copper is one of the most applicable metals in C–H activation reactions. Its versatility makes it possible to develop not only C–H amination procedures, but also to achieve C–H arylation, and fluorination, among others. Using these different methodologies, several different bioactive motifs can be directly achieved and evaluated. Amongst with iron, nickel, and cobalt, copper represents one of the most promising metals for future progress in organic synthesis.

### Zinc-catalyzed C–H activation

**Scheme 83:**
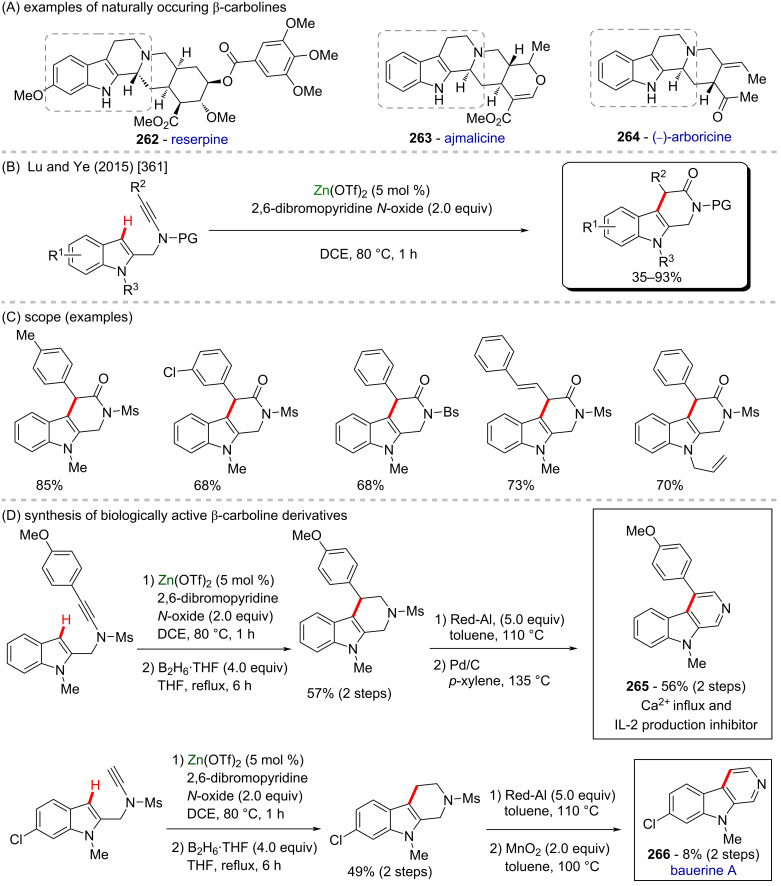
(A) Examples of natural β-carbolines; (B and C) an example of a zinc-catalyzed C–H functionalization; (D) synthesis of biologically active β-carbolines.

Zinc is the last 3d-metal in the periodic table, and it is extremely useful due to its numerous applications in different fields such as animal [350] and human diets [351], galvanizing solutions [352], cosmetics [353], and potential pharmaceutics [354]. Due to its relatively low costs it has been widely studied as a metallic source of several catalysts, either in its metallic [355], or in its ionic form [356]. Zinc-based catalysts have been successfully applied to the hydroamination of several ynamides [357], in the synthesis of chiral 2-arylpyrrolidines of pharmacological importance such as compounds **262**–**264**) [358], and in C–H-bond activation reactions [359–360]. Its use in the synthesis of biologically active compounds via C–H-bond activation remains a challenge, with only few examples being reported in the literature so far. One good example is presented in a work published by Lu, Ye and co-workers in 2015 [361]. In this work, the authors described an intramolecular zinc-catalyzed C–H functionalization towards the formation of several β-carboline derivatives (Scheme 83B). This scaffold is present in several important natural compounds with valuable biological activities (Scheme 83A) [362]. Using this singular method, it was possible to obtain many cyclic benzyl- and indole-lactams in good to excellent yields (Scheme 83C). Following the same procedure, it was possible to synthesize the β-carboline **265** that acts as Ca^2+^ influx and IL-2 production inhibitor, and the natural β-carboline bauerine A (**266**) (Scheme 83D).

**Scheme 84:**
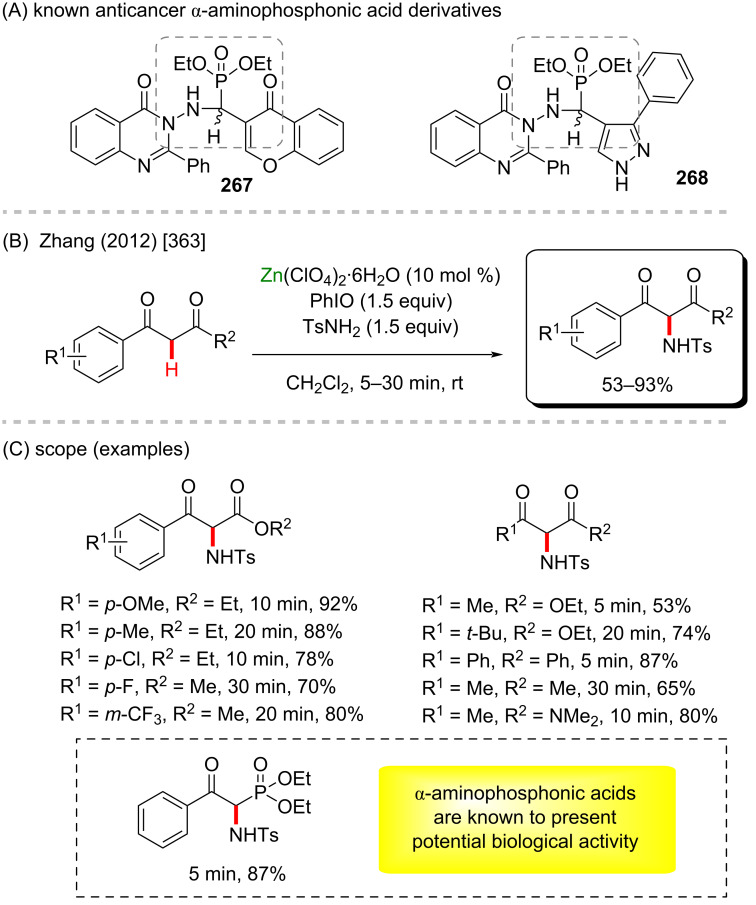
(A) Examples of anticancer α-aminophosphonic acid derivatives; (B and C) an example of a zinc-catalyzed C–H amination.

In 2012, Zhang and co-workers described a C–H amination of β-carbonylated compounds and one example of a β-ketophosphonate compound, in the presence of tosylamine and iodosobenzene [363]. The in situ-generated PhI=NTs, along with zinc perchlorate led to the formation of several aminated products in moderate to excellent yields (Scheme 84B and C), including α-aminophosphoric acid derivatives which resemble structures of known previously studied anticancer compounds **267** and **268** (Scheme 84A) [364].

In contrast to the previously discussed metals, zinc is lacking examples of its use as catalyst for the synthesis of bioactive compounds via C–H activation processes. It is also a cheap and low-toxic metal, and therefore it might have more to offer in this particular field. Therefore, deeper studies are worthy to be developed in order to make it easier and cheaper to obtain some complex but valuable potent bioactive substances using zinc catalysis.

## Conclusion

This review visualizes not only the versatile applicability of the 3d metals as powerful catalysts in C–H functionalization methods, but in a deeper analysis, it allows to perceive that these accessible catalysts enable an easier formation of important biologically active substances. The access to crucial medicines that save thousands of lives every day can be difficult in several places due to the costs involved in their production and, of course, the subsequent cost to the final consumers. Therefore, it is highly desirable to support and develop innovative and cheaper synthetic methods for the production of already known drugs and to discover new ones. The combination of less expensive 3d metals and C–H activation processes towards the synthesis of biologically active molecules could enable this essential goal to be achieved.
